# Identification and analysis of candidate fungal tRNA 3'-end processing endonucleases tRNase Zs, homologs of the putative prostate cancer susceptibility protein ELAC2

**DOI:** 10.1186/1471-2148-10-272

**Published:** 2010-09-06

**Authors:** Wei Zhao, Haiyan Yu, Shuzhen Li, Ying Huang

**Affiliations:** 1 Nanjing Engineering and Technology Research Center for Microbiology, Jiangsu Key Laboratory for Biodiversity and Biotechnology, School of Life Sciences, Nanjing Normal University, Nanjing 210046, China

## Abstract

**Background:**

tRNase Z is the endonuclease that is responsible for the 3'-end processing of tRNA precursors, a process essential for tRNA 3'-CCA addition and subsequent tRNA aminoacylation. Based on their sizes, tRNase Zs can be divided into the long (tRNase Z^L^) and short (tRNase Z^S^) forms. tRNase Z^L ^is thought to have arisen from a tandem gene duplication of tRNase Z^S ^with further sequence divergence. The species distribution of tRNase Z is complex. Fungi represent an evolutionarily diverse group of eukaryotes. The recent proliferation of fungal genome sequences provides an opportunity to explore the structural and functional diversity of eukaryotic tRNase Zs.

**Results:**

We report a survey and analysis of candidate tRNase Zs in 84 completed fungal genomes, spanning a broad diversity of fungi. We find that tRNase Z^L ^is present in all fungi we have examined, whereas tRNase Z^S ^exists only in the fungal phyla Basidiomycota, Chytridiomycota and Zygomycota. Furthermore, we find that unlike the Pezizomycotina and Saccharomycotina, which contain a single tRNase Z^L^, *Schizosaccharomyces *fission yeasts (Taphrinomycotina) contain two tRNase Z^L^s encoded by two different tRNase Z^L ^genes. These two tRNase Z^L^s are most likely localized to the nucleus and mitochondria, respectively, suggesting partitioning of tRNase Z function between two different tRNase Z^L^s in fission yeasts. The fungal tRNase Z phylogeny suggests that tRNase Z^S^s are ancestral to tRNase Z^L^s. Additionally, the evolutionary relationship of fungal tRNase Z^L^s is generally consistent with known phylogenetic relationships among the fungal species and supports tRNase Z^L ^gene duplication in certain fungal taxa, including *Schizosaccharomyces *fission yeasts. Analysis of tRNase Z protein sequences reveals putative atypical substrate binding domains in most fungal tRNase Z^S^s and in a subset of fungal tRNase Z^L^s. Finally, we demonstrate the presence of pseudo-substrate recognition and catalytic motifs at the N-terminal halves of tRNase Z^L^s.

**Conclusions:**

This study describes the first comprehensive identification and sequence analysis of candidate fungal tRNase Zs. Our results support the proposal that tRNase Z^L ^has evolved as a result of duplication and diversification of the tRNase Z^S ^gene.

## Background

The endonuclease tRNase Z (also called RNase Z or 3'-tRNase) participates in maturation of tRNA 3'-end by removing the 3'-trailer sequence from tRNA precursors (pre-tRNAs, for reviews, see [1-4]). It belongs to the metallo-*β*-lactamase (MBL) superfamily, the members of which have diverse functions from hydrolysis and inactivation of *β*-lactam antibiotics to processing of RNA precursors [5-9]. Other nucleases in the MBL superfamily that act on nuclei acids include members of the *β*-CASP (MBL-associated CISF Artemis SNM1/PSO2) family: the cleavage and polyadenylation specificity factor 73 kDa subunit (CPSF-73) [10] and the Integrator complex subunit 11 (Int11) [11], which are involved in eukaryotic mRNA and small nuclear RNA (snRNA) 3'-end formation, respectively; RNase J, which functions in rRNA maturation and mRNA stability in bacteria [12] and the eukaryotic Pso2/Snm1/Artemis proteins, which play a role in DNA repair [7]. Although displaying distinct substrate specificity defined by their specific domains, these proteins appear to have a similar catalytic mechanism since their active sites are composed of highly conserved motifs including the histidine motif (HxHxDH, where x is any hydrophobic residues).

There are two forms of tRNase Z. The long form (tRNase Z^L^) with 800-900 aa (amino acids) is about twice the size of the short form (tRNase Z^S^) with 300-400 aa [9]. Sequence analysis suggests that tRNase Z^L ^has evolved by gene duplication from tRNase Z^S ^followed by sequence divergence [9]. The species distribution of tRNase Z is not homogenous. tRNase Z^S ^exists in all three domains of life (i.e. Bacteria, Archaea, and Eukarya) whereas tRNase Z^L ^has been found only in eukaryotes so far. The number of tRNase Zs varies among different organisms. The largest number of tRNase Zs was detected in the plant *Arabidopsis thaliana *(two tRNase Z^S^s and two tRNase Z^L^s) [13]. The fission yeast *Schizosaccharomyces pombe *contains two tRNase Z^L^s. Unexpectedly, the human genome encodes one tRNase Z^S ^and one tRNase Z^L^. Human tRNase Z^L ^gene (also termed *ELAC2*) was originally identified as the first prostate cancer susceptibility gene by positional cloning [9]. However, the mechanism by which specific mutations in human tRNase Z^L ^lead to an increased risk of prostate cancer remains unknown. In contrast, the budding yeast *Saccharomyces cerevisiae*, the fruit fly *Drosophila melanogaster *and the nematode worm *Caenorhabditis elegans *have just one tRNase Z^L^.

An intriguing question is why species have evolved to have more than one tRNase Z. One explanation is that additional tRNase Zs are targeted to organelles such as mitochondria and chloroplasts in which organelle-encoded pre-tRNAs must also be processed. Indeed, one of two *S. pombe *tRNase Z^L^s is targeted to the mitochondria, and has been suggested to play a role in mitochondrial-encoded pre-tRNA processing [14]. In *A. thaliana*, three of four tRNase Z^L^s are targeted to organelles [13]. However, it appears that the majority of tRNase Z^L^s identified thus far are imported both into the nucleus and mitochondria. Another explanation is that additional tRNase Zs may provide a back-up mechanism for nuclear tRNA 3'-end processing. The third explanation is that additional tRNase Zs may have different functions.

Recently, tRNase Z^L ^itself has been either demonstrated or suggested to have additional functions other than tRNA 3'-end processing. For example, human tRNase Z^L ^has been shown to play a role in generation of non-tRNA noncoding RNAs [15,16] and viral microRNAs (miRNAs) [17]. Moreover, human tRNase Z^L ^has been proposed to cleave a subset of miRNAs in the cytoplasm [18]. In *S. cerevisiae*, tRNase Z^L ^has been suggested to have additional functions including rRNA biogenesis, mRNA splicing and mitochondrial maintenance [19]. Similarly, our previous study also suggested that the nuclear-localized tRNase Z^L ^in *S. pombe *may play a role beyond tRNA 3'-end processing [14].

Our current understanding of tRNase Z evolution is limited since there has been only one comprehensive survey on tRNase Z^S^s from prokaryotes [3]. Of eukaryotes, the Fungi is a large and diverse kingdom encompassing roughly 1.5 million species and spanning one billion years of evolution [20]. Sequence-based phylogenies show that the Chytridiomycota is the most basal phylum (group) among the Fungi, followed by the Zygomycota, with the Ascomycota and Basidiomycota as two largest phyla that together comprise the subkingdom Dikarya (also referred to as the "Higher Fungi") [21-24]. The Ascomycota (also known as sac fungi, yeasts or ascomycetes) is the largest and most diverse phylum in the Fungi, accounting for approximately 75% of all known fungi. Many popular model organisms such as *S. cerevisiae*, *S. pombe*, *Neurospora crassa*, *Aspergillus nidulans *and *Candida albicans *are classified in this phylum. This phylum is further divided into three major monophyletic subphyla (subgroups): Pezizomycotina, Saccharomycotina and Taphrinomycotina [25]. The Pezizomycotina (also known as euascomyces) is the largest subphyla and contains over 90% of total Ascomycota species. They are multicellular filamentous fungi and grow by hyphal extension and branching. In contrast, the Saccharomycotina (also known as true yeasts) comprises the majority of unicellular species. The Taphrinomycotina is thought to be the earliest diverging group sister to the Saccharomycotina and Pezizomycotina. It constitutes a diverse group of organisms including unicellular yeast (for example, *Schizosaccharomyces*), multicellular filamentous fungi, and dimorphic fungi that can switch between yeast and hyphal growth forms. Like the Pezizomycotina, the Basidiomycota consists of primarily filamentous fungi.

Currently, most of eukaryotic species with sequenced genomes belong to the kingdom Fungi. The public fungal genome databases cover a broad range of fungal taxonomic groups with the majority coming from the Ascomycota and Basidiomycota. The availability of a large number of fungal genome sequences, together with the vast diversity of fungal morphology and lifestyle, provides an opportunity to identify tRNase Zs in the kingdom Fungi and to study evolution of eukaryotic tRNase Z.

In the present study, we performed a comprehensive survey of candidate tRNase Zs from 84 publicly available fungal genomes. To explore the evolutionary relationship among fungal tRNase Zs, we conducted a phylogenetic analysis of predicted fungal tRNase Zs. Finally, we examined their domain architectures. Our results support the view that tRNase Z^L ^comes from tRNase Z^S^.

## Results

### Identification of putative fungal tRNase Zs

As part of our efforts to better understand functional and structural diversity of tRNase Zs, we conducted extensive BLAST and PSI-BLAST homology searches against the publicly available fungal genome databases. Currently, the majority of sequenced species belong to the Dikarya, with a much higher proportion of Ascomycota species. Since other fungal phyla are poorly represented in public databases (three Zygomycota, three Microspordia, and three Chytridiomycota species), it is difficult to assess the true diversity of tRNase Z in these basal groups of fungi.

The initial candidates from the BLAST and PSI-BLAST were verified by multiple sequence alignment and reciprocal searches against the GenBank. Protein sequence alignment revealed a number of incorrectly predicted candidates, most likely due to misprediction of exon/intron boundaries or existence of gaps in the genome sequence. For example, the sequence (Broad accession no. CC1G_14814.2) annotated as the candidate Basidiomycete *Coprinopsis cinerea *tRNase Z in the fungal genome database at the Broad Institute has mispredicted exon/intron junctions. This 946-aa-long candidate is devoid of a histidine motif, which is a signature motif for the MBL superfamily, indicating that the exon encoding the histidine motif was likely mispredicted. After re-evaluating intron splicing pattern of the gene sequence, we were able to predict the exon encoding the histidine motif. The correctly predicted protein has 967 aa, and has the histidine motif. The sequence annotated as the candidate Pezizomycotina *Botrytis cinerea *(also named *Botryotinia fuckeliana*) tRNase Z (Broad accession no. BC1G_03733.1) is an example of misprediction due to the presence of sequence gaps in the genome. This sequence has 444 aa. However, examination of the genomic sequence revealed that its 5'-coding sequence contains gaps. Thus, this sequence was excluded. In back-searches, no candidate that shows homology to metallo-*β*-lactamase was found, but a limited number of candidates were found to show homology to the yeast homolog of CPSF-73 (Ysh1). Such candidates were also excluded from our final list. However, we cannot rule out the possibility that certain candidates may not be correctly predicted despite our efforts devoted to verification of these candidates.

We identified a total of 90 candidate tRNase Z^L^s and 19 candidate tRNase Z^S^s proteins from 84 fungal species including 67 Ascomycota, 14 Basidiomycota and 3 Chytridiomycota (Additional file 1). Candidate tRNase Zs from two taxonomic groups, the Zygomycota and Microspordia, were not listed since their full-length protein sequences could not be correctly predicted. Of the proteins identified here, only tRNase Z^L^s from *S. cerevisiae *and *S. pombe *have been experimentally characterized [14,19,25-27].

All species of the Ascomycota we have examined lack tRNase Z^S^. The Pezizomycotina and Saccharomycotina species have a single tRNase Z^L^. Surprisingly, in contrast to the Pezizomycotina and Saccharomycotina species, all four sequenced *Schizosaccharomyces *species (*S. pombe*, *Schizosaccharomyces octosporus*, *Schizosaccharomyces japonicus *and a recently described *Schizosaccharomyces cryophobus*) in the Taphrinomycotina have two tRNase Z^L^s, which we term tRNase Z^L1 ^and tRNase Z^L2^, respectively. tRNase Z^L1^s and tRNase Z^L2^s have been either shown or predicted to localize to the nucleus and mitochondria, respectively (Additional file 2 and data not shown) [28]. Since in the current databases, all sequenced Taphrinomycotina species come from only *Schizosaccharomyces*, it would be interesting to see whether species in other genera also contain two tRNase Z^L^s.

Like Ascomycota species, all sequenced Basidiomycota species (except for *Agaricus bisporus*) have a single tRNase Z^L^. However, unlike the situation in the Ascomycota, tRNase Z^S ^was found in all sequenced Basidiomycota species. While the majority of Basidiomycota species have a single tRNase Z^S^, four Basidiomycota species (*A. bisporus*, *C. cinerea*, *Laccaria bicolor *and *Postia placenta*) have two tRNase Z^S^s. Among the Basidiomycota species examined, *A. bisporus *has the largest number of tRNase Zs (two tRNase Z^L^s and two tRNase Z^S^s). The number of tRNase Z seems to be variable in the three sequenced chytrid species. *Allomyces macrogynus *and *Spizellomyces punctatus *have two tRNase Z^L^s whereas *Batrachochytrium dendrobatidis *appears to have one tRNase Z^L^. Moreover, tRNase Z^S ^was only identified in *S. punctatus*. Although we could not correctly predict the full-length tRNase Zs in three sequenced Zygomycota species (*Rhizopus oryzae*, *Mucor circinellodes *and *Phycomyces blakesleeanus*) and in three sequenced Microspordian species (*Encephalitozoon cuniculi*, *Enterocytozoon bieneusi *and *Nosema ceranae*), it is important to note that tRNase Z^S ^appears to exist in all sequenced Zygomycota fungi but not in sequenced Microspordian fungi known for extreme genome reduction and compaction [29].

### Phylogenetic analysis

**Figure 1:**
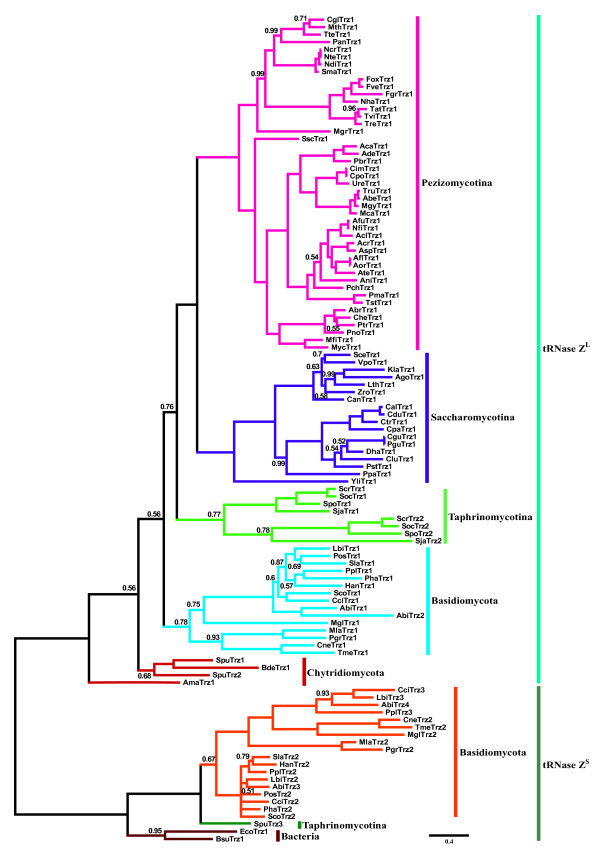
**A phylogenetic tree inferred using Bayesian analysis of fungal tRNase Zs**. Markov chain Monte Carlo (MCMC) algorithm was used to assess the reliability of nodes in the phylogeny. Numbers above or below branches represent posterior probabilities for each node which were generated by using Bayesian MCMC sampling. Only Bayesian posterior probability values less than 1.00 are indicated. The scale bar indicates 0.4 nucleotide substitutions per site. The accession number for fungal tRNase Zs can be found in Additional file 1. Taxonomic designations are indicated on the right side of the tree.

To explore evolutionary relationships among fungal tRNase Zs, we performed a phylogenetic analysis of the amino acid sequences of all tRNase Zs predicted from the fungal genome databases. Figure 1 shows the phylogenetic tree for 109 fungal tRNase Zs. In addition to the fungi species, tRNase Z^S^s from *B. subtilis *and *E. coli *were included as reference. It is seen that tRNase Zs are clearly separated into a small cluster containing both fungal and bacterial tRNase Z^S^s and a large cluster containing fungal tRNase Z^L^s. Moreover, within the tRNase Z^L ^cluster, tRNase Z^L^s can be grouped according to their taxonomic classification (Figure 1), although some Bayesian posterior probability values for grouping are not strong. It appears that the phylogenetic relationships among fungal tRNase Z^L^s is basically congruent with the currently accepted fungi phylogenies based on cladistic analyses of RNA and/or protein sequences [21-24]. Notably, tRNase Z^L2^s from four fission yeasts together form a group sister to a group formed by tRNase Z^L1^s from the same fission yeasts, albeit with a posterior probability of only 0.77. Likewise, the two tRNase Z^L^s in the Basidiomycete *Agaricus bisporus *(AbiTrz1 and AbiTrz2) are sister to each other with a posterior probability value of 1.

### Analysis of fungal tRNase Z^L^s

The sizes of predicted tRNase Z^L^s vary considerably among fungal species, ranging from 648 to 1140 aa with an average size of ~924 aa. The variation in protein size is due to a high degree of length and sequence variation of N-terminal and C-terminal extensions and many insertions and/or deletions. Remarkably, tRNase Z^L^s in *Sordaria macrospora *and three *Neurospora *species (*N. crassa*, *Neurospora discreta *and *Neurospora discreta*) have a very long N-terminal extension (~200 residues). This feature appears to be family-specific since all these species belong to the family *Sordariaceae*.

A number of fungal tRNase Z^L^s have a variable length N-terminal extension predicted to contain a canonical MTS (Additional file 2). In addition, tRNase Z^L2^s from four *Schizosaccharomyces *species we have examined also contain a putative MTS in their N-terminal extensions. The N-terminal extensions found in fungal tRNase Z^L^s may serve as transit sequences for directing the proteins to the mitochondria. It is interesting to note that tRNase Z^L^s from *D. melanogaster *and humans also contain a canonical MTS.

**Figure 2:**
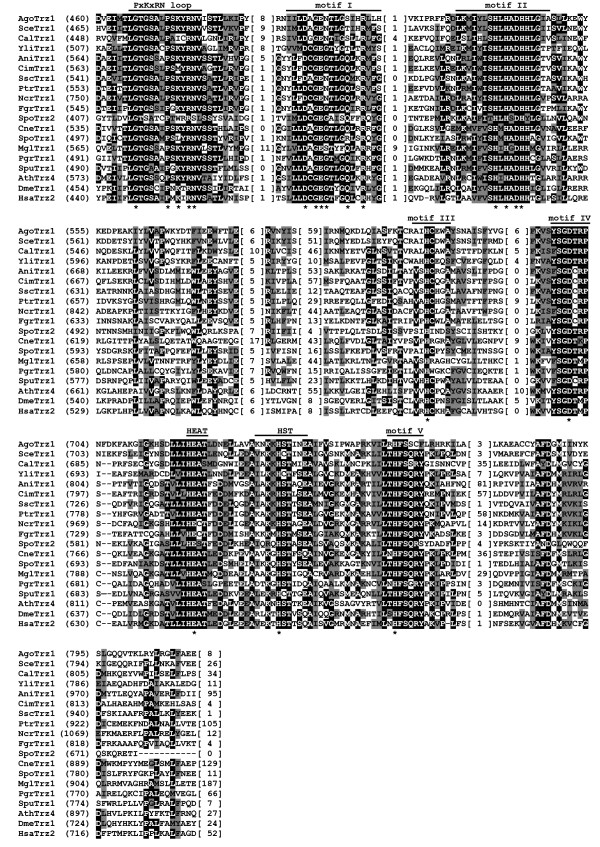
**Sequence conservation between the C-terminal halves of fungal and non-fungal eukaryotic tRNase Z^L^s**. Multiple sequence alignment of the C-terminal halves of representative fungal and non-fungal eukaryotic tRNase Z^L^s. Fungal tRNase Z^L^s are from *A. gossypii *(AgoTrz1), *S. cerevisiae *(SceTrz1) [19], *C. albicans *(CalTrz1), *Yarrowia lipolytica *(YliTrz1), *A. nidulans *(AniTrz1), *Coccidioides immitis *(CimTrz1), *Sclerotinia sclerotiorum *(SscTrz1), *Pyrenophora tritici-repentis *(Ptrtrz1), *N. crassa *(NcrTrz1), *Fusarium graminearum *(FgrTrz1), *S. pombe *(SpoTrz1 and SpoTrz2) [14], *Cryptococcus neoformans *(CneTrz1), *M. globosa *(MglTrz1), *Puccinia graminis *(Pgr Trz1) and *S. punctatus *(SpuTrz1). Non-fungal tRNase Z^L^s are from *A. thaliana *(AthTrz4) [13], *D. melanogaster *(DmeTrz1) [54] and *Homo sapiens *(HsaTrz2) [9]. Protein accession numbers are described in Table 1. The alignment was constructed using the program Clustal W [49]. Identical residues are on a black background and conserved residues shaded in gray. Also indicated above the sequence alignment are the conserved motifs involved in substrate binding and catalysis. The conserved motifs are labeled according to references [30,31,44]. Numbers in parentheses are the positions of the amino acid sequences. The numbers in brackets indicate the length of the region in tRNase Z, which are species-specific and could not be correctly aligned. Hyphens represent gaps introduced into sequences for maximum alignment. The positions of amino acid residues of the *D. melanogaster *tRNase Z^L ^critical for catalytic efficiency [30,31] are indicated by asterisk.

**Figure 3:**
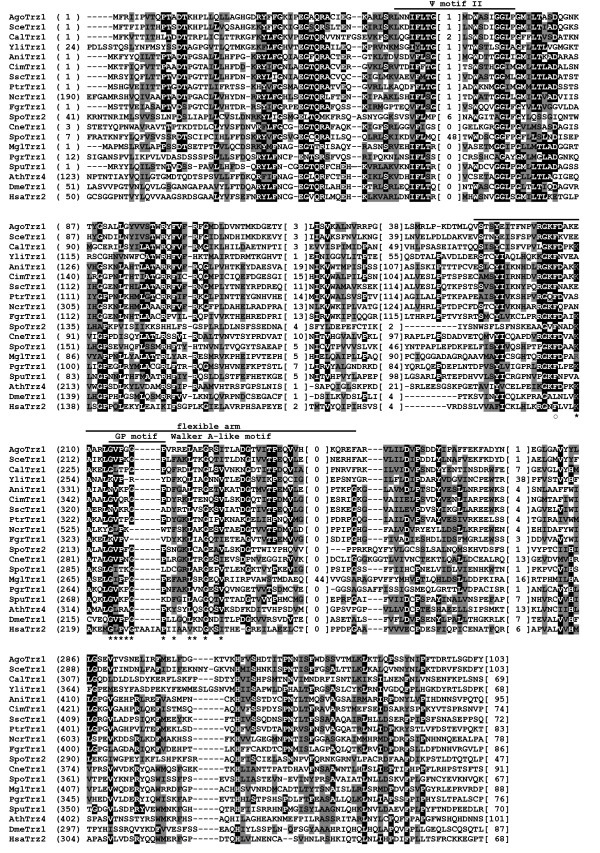
**Sequence conservation between the N-terminal halves of fungal and non-fungal eukaryotic tRNase Z^L^s**. Multiple sequence alignment of the N-terminal halves of representative fungal and non-fungal eukaryotic tRNase Z^L^s according to the same legend as in Figure 2. The positions of amino acid residues of the *D. melanogaster *tRNase Z^L ^that contribute to substrate binding are marked with an asterisk [44]. Open circle denotes the residue in the *D. Melanogaster *tRNase Z^L ^that makes the largest contribution to substrate binding [44].

To assess the extent of sequence and structural conservation among fungal tRNase Z^L^s, we aligned sixteen tRNase Z^L ^protein sequences from fifteen taxonomically diverse fungal species including eleven species of the Ascomycota, three species of the Basidiomycota, and one species of the Chytridiomycota (Table 1). Since the amino acid sequence of tRNase Z^L ^can be divided into an N-terminal half, which contains a substrate binding site, and a C-terminal half, which contains a catalytic center and most of the conserved motifs, we aligned the N- and C-terminal halves of tRNase Z^L^s separately, and first examined the C-terminal half. For comparison, we also included non-fungal eukaryotic tRNase Z^L^s from *D. melanogaster*, *A. thaliana *and humans. Figures 2 and 3 show sequence comparison of representative fungal and non-fungal eukaryotic tRNase Z^L^s (For a full list of all aligned fungal tRNase Z^L^s, see Additional file 3).

**Table 1:** Representatives of candidate fungal tRNase Zs used in sequence alignment

Species^#^	Taxonomy	Protein name	Form	Accession Number	Database	No. aa^+^
Ashbya gossypii (Ago)	Ascomycota	AgoTrz1	tRNase Z^L^	NP_984308	NCBI	821
Aspergillus nidulans (Ani)	Ascomycota	AniTrz1	tRNase Z^L^	ANID_11892.1	Broad	1083
Candida albicans (Cal)	Ascomycota	CalTrz1	tRNase Z^L^	XP_717703	NCBI	857
Coccidioides immitis (Cim)	Ascomycota	CimTrz1	tRNase Z^L^	CIHG_00067.1	Broad	962*
Fusarium graminearum (Fgr)	Ascomycota	FgrTrz1	tRNase Z^L^	FGSG_06635.3	Broad	840
Neurospora crassa (Ncr)	Ascomycota	NcrTrz1	tRNase Z^L^	NCU00232.4	Broad	1099
Pyrenophora tritici-repentis (Ptr)	Ascomycota	PtrTrz1	tRNase Z^L^	XP_001940780	NCBI	988*
Saccharomyces cerevisiae (Sce)	Ascomycota	SceTrz1	tRNase Z^L^	NP_013005	NCBI	838
Schizosaccharomyces pombe (Spo)	Ascomycota	SpoTrz1	tRNase Z^L1^	SPAC1D4.10	Broad	809
Schizosaccharomyces pombe (Spo)	Ascomycota	SpoTrz2	tRNase Z^L2^	SPBC3D6.03C	Broad	678
Sclerotinia sclerotiorum (Ssc)	Ascomycota	SscTrz1	tRNase Z^L^	XP_001586541	NCBI	832
Yarrowia lipolytica (Yli)	Ascomycota	YliTrz1	tRNase Z^L^	XP_500027	NCBI	815
Cryptococcus neoformans (Cne)	Basidiomycota	CneTrz1	tRNase Z^L^	CNBG_1589.2	Broad	1035
Malassezia globosa (Mgl)	Basidiomycota	MglTrz1	tRNase Z^L^	XP_001729151	NCBI	1109
Puccinia graminis (Pgr)	Basidiomycota	PgrTrz1	tRNase Z^L^	PGTG_11198.2	Broad	854
Spizellomyces punctatus (Spu)	Chytridiomycota	SpuTrz2	tRNase Z^L^	SPPG_00513.2	Broad	799*
Agaricus bisporus (Abi)	Basidiomycota	AbiTrz3	tRNase Z^S^	106812	JGI	376
Agaricus bisporus (Abi)	Basidiomycota	AbiTrz4	tRNase Z^S^	123256	JGI	469
Coprinopsis cinerea (Cci)	Basidiomycota	CciTrz2	tRNase Z^S^	CC1G_14603.2	Broad	390
Coprinopsis cinerea (Cci)	Basidiomycota	CciTrz3	tRNase Z^S^	CC1G_03806.2	Broad	544
Laccaria bicolor (Lbi)	Basidiomycota	LbiTrz2	tRNase Z^S^	XP_001876619	NCBI	406
Laccaria bicolor (Lbi)	Basidiomycota	LbiTrz3	tRNase Z^S^	XP_001874963	NCBI	476
Malassezia globosa (Mgl)	Basidiomycota	MglTrz2	tRNase Z^S^	EDP42443	NCBI	484*
Melampsora laricis-populina (Mla)	Basidiomycota	MlaTrz2	tRNase Z^S^	111950	JGI	473*
Postia placenta (Ppl)	Basidiomycota	PplTrz2	tRNase Z^S^	94043	JGI	386
Postia placenta (Ppl)	Basidiomycota	PplTrz3	tRNase Z^S^	92595	JGI	483*
Puccinia graminis (Pgr)	Basidiomycota	PgrTrz2	tRNase Z^S^	PGTG_13150.2	Broad	498
Spizellomyces punctatus (Spu)	Chytridiomycota	SpuTrz3	tRNase Z^S^	SPPG_06028.2	Broad	395

^#^Species abbreviations used in this study are shown in parentheses.

^+ ^The number of amino acids in fungal tRNase Zs

* Indicates that mispredicted sequences obtained from the databases have been corrected.

Although tRNase Z^L^s from closely related species share the high degree of sequence similarity, the sequence similarity among fungal tRNase Z^L^s is low. Overall, sequence conservation of fungal tRNase Z^L^s is largely confined to highly conserved Motifs I-V (Motif II is also called the histidine motif) and the PxKxRN, HEAT and HST loop motifs at the C-terminal halves of the proteins (Figure 2). Except for the PxKxRN loop and Motif I, which were found to play a role in pre-tRNA acceptor stem binding and CCA anti-determination [30], all other motifs are involved in zinc binding and catalysis [31-33]. Motifs I-V contain invariant histidine and/or aspartate residues essential for the tRNase Z activity. In particular, the histidine and aspartate residues in the histidine motif, together with the histidine residues in Motifs III and V and the aspartate residue in Motif IV are involved in the coordination of the two zinc ions at the catalytic center [1,3,4].

All characterized tRNase Zs contain a characteristic domain of 30~50 aa residues, termed a flexible arm (or an exosite), which is important for substrate binding [34,35]. Based on sequence comparison, three types of flexible arms, termed the zinc-dependent phosphodiesterase (ZiPD)-, ELAC2- and *Thermotoga maritima *(TM)-type flexible arms, have been found in tRNase Zs. The ZiPD- and ELAC2-type flexible arms were typical for bacterial tRNase Z^S^s and eukaryotic tRNase Z^L^s, respectively, whereas the TM-type flexible arm is an atypical one found only in tRNase Z^S^s from *T. maritima *and *A. thaliana*. Interestingly, tRNase Z^S ^in *T. maritima *itself is an atypical enzyme as it cleaves CCA-containing pre-tRNAs after CCA. Although having sequence and length variations, both ZiPD- and ELAC2-type flexible arms comprise a GP motif rich in glycine and proline residues [35], followed by a Walker A-like motif [9]. However, unlike the ZiPD- and ELAC2-type flexible arms, the TM-type flexible arm is short and lacks the GP motif. Instead it contains a short stretch of mainly basic amino acids [35].

**Figure 4:**
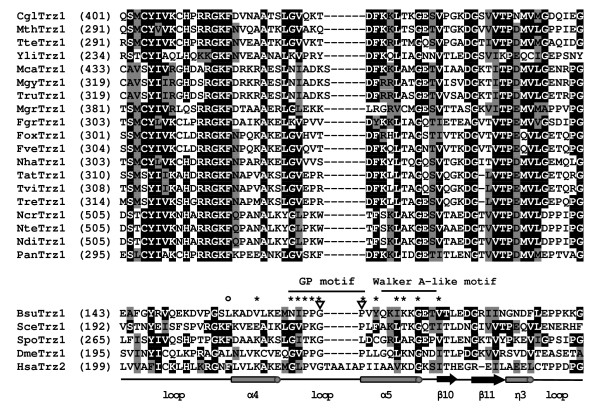
**Amino acid alignments of putative atypical flexible arms in fungal tRNase Z^L^s**. The top panel alignment shows putative atypical flexible arms found in certain fungal tRNase Z^L^s from *Chaetomium globosum *(CglTrz1), *Myceliophthora thermophila *(MthTrz1), *Thielavia terrestris *(TteTrz1), *Y. lipolytica *(YliTrz1), *Microsporum canis *(McaTrz1), *Microsporum gypseum *(MgyTrz1), *Trichophyton rubrum *(TruTrz1), *Magnaporthe grisea *(MgrTrz1), *F. graminearum *(FgrTrz1), *Fusarium oxysporum *(FoxTrz1), *Fusarium verticillioides *(FveTrz1), *Nectria haematococca *(NhaTrz1), *Trichoderma atroviride *(TatTrz1), *Trichoderma virens *(TviTrz1), *Trichoderma reesei *(TreTrz1), *N. crassa *(NcrTrz1), *N. tetrasperma *(NteTrz1), *N. discreta *(NdiTrz1), *Podospora anserina *(PanTrz1). The bottom panel alignment shows typical flexible arms found in tRNase Zs from *B. subtilis *(BsuTrz1) [55], *S. cerevisiae *(SceTrz1) [19], *S. pombe *(SpoTrz1) [14], *D. melanogaster *(DmeTrz1) [54] and *H. sapiens *(HsaTrz2) [9]. The predicted tRNase Z^L^s are defined in Additional file 1. The GP and Walker A-like motifs are indicated. Invariant and conserved amino acids are highlighted in Black and gray, respectively. The Asterisk indicates key residues in the *D. Melanogaster *tRNase Z^L ^that contribute to substrate binding [44]. The open circle denotes the residue in the *D. Melanogaster *tRNase Z^L ^that makes the largest contribution to substrate binding. Inverted triangles indicate the conserved glycine and proline residues in the GP motif, which are no longer conserved in certain fungal tRNase Z^L^s. Dark shading indicates identical residues, and gray shading designates conserved residues. Secondary structure assignment is based on the structure of the *B. subtilis *tRNase Z^S ^[34]. The secondary structures for *α*-helices, *β*-strands and 3_10_-helix are indicated with Greek letters. Asterisks indicate key residues in the *D. Melanogaster *tRNase Z^L ^that contribute to substrate binding [44].

As anticipated, ELAC2-type flexible arm containing both the GP and Walker A-like motifs was found in the majority of the N-terminal halves of fungal tRNase Z^L^s (Figure 3 and Additional file 3). Unexpectedly, a subset of fungal tRNase Z^L^s appear to have an atypical ELAC2-type flexible arm which either lacks or contains an incomplete GP-motif (Figure 4). Moreover, unlike the TM-type flexible arm, this atypical flexible arm does not encompass a short cluster of basic amino acids.

**Figure 5:**
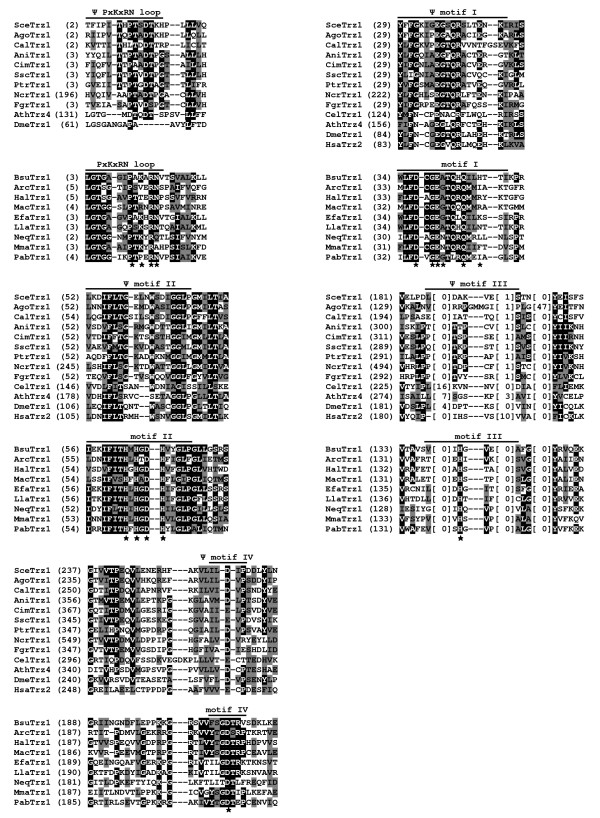
**Pseudo-motifs in representative candidate fungal tRNase Z^L^s**. The top panel shows alignment for pseudo substrate recognition and catalytic motifs found in tRNase Z^L^s from *S. cerevisiae *(SceTrz1) [19], *A. gossypii *(AgoTrz1), *C. albicans *(CalTrz1), *A. nidulans *(AniTrz1), *C. immitis *(CimTrz1), *S. sclerotiorum *(SscTrz1), *P. tritici-repentis *(PtrTrz1), *N. crassa *(NcrTrz1), *F. graminearum *(FgrTrz1), *C. elegans *(CelTrz1)[56], *D. melanogaster *(DmeTrz1) [54], *A. thaliana *(AthTrz4) [13] and humans (HsaTrz2) [9]. The bottom panel shows alignment for the corresponding conserved motifs in tRNase Zs from *B. subtilis *(BsuTrz1) [55], *Archaeoglobus fulgidus *(ArcTrz1; accession no. NP_069772), *Halobacterium salinarum *(HalTrz1; accession no. NP_280881), *Methanosarcina acetivorans *(MacTrz1; accession no. NP_617924), *Enterococcus faecalis *(EfaTrz1; accession no.NP_815399), *Lactococcus lactis subsp *(LlaTrz1; accession no. NP_266786), *Nanoarchaeum equitans *(NeqTrz1; accession no. NP_963358), *Methanococcus maripaludis *(MmaTrz1; accession no. NP_988026) and *Pyrococcus abyssi *(PabTrz1; accession no. NP_126781). The pseudo-PxKxRN loops of *C. elegans *and human tRNase Z^L^s are not included since they are indiscernible. The candidate fungal tRNase Z^L^s are defined in Additional file 1. GenBank accession numbers that are not listed in Additional file 1 are indicated. The alignment was extended to include surrounding regions to illustrate the further homology revealed by searching the Conserved Domain database (CDD), which could aid in the identification of pseudo-motifs. Similar or identical amino acid residues are shaded as described in the legend to Figure 2. The positions of amino acid residues of the *D. melanogaster *tRNase Z^L ^critical for catalytic efficiency [30,31] are indicated by asterisk.

Besides the flexible arm, the conserved domain search against the NCBI Conserved Domain Database (CDD) http://www.ncbi.nlm.nih.gov/Structure/cdd/cdd.shtml combined with manual evaluation revealed regions of sequences that match the Motifs I-IV and the PxKxRN loop in the N-terminal halves of tRNase Z^L^s. However, they appear to be nonfunctional as they differ from their original patterns in many positions including the key residues critical for tRNase Z functions. We collectively termed these sequences pseudo-motifs. Figure 5 shows pseudo-motifs in representative candidate fungal tRNase Z^L^s. For comparison, we also include four metazoan tRNase Z^L^s from *C. elegans*, *D. melanogaster*, *A. thaliana *and humans. Since the pseudo-PxKxRN loops of *C. elegans *and human tRNase Z^L^s are indiscernible from their protein sequences, they are not included. Except for the pseudo-histidine motif [36], these pseudo-motifs have not been reported previously, which may reflect the difficulty in identifying these sequences. It appears that only the pseudo-histidine motif is widespread; the distributions of other pseudo-motifs are highly variable among fungal tRNase Z^L^s. Moreover, pseudo-Motif V could not be identified. It is likely that some of pseudo-motifs may have diverged too far and thus are no longer similar enough to the conserved motifs for homology to be detected by the NCBI conserved domain search. It is also notable that these pseudo-motifs were in the same relative order as their original motifs in tRNase Z^S^s and in the C-terminal halves of tRNase Z^L^s.

### Analysis of fungal tRNase Z^S^s

**Figure 6:**
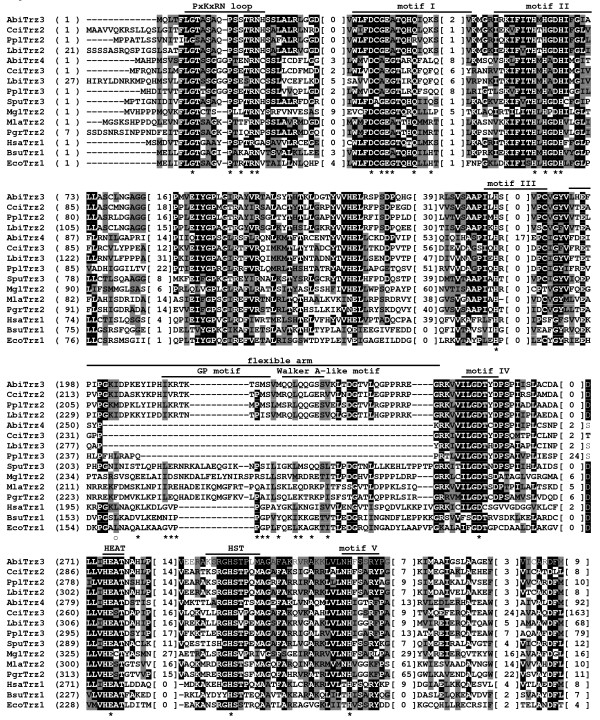
**Multiple alignment fungal and non-fungal eukaryotic tRNase Z^S^s**. tRNase Z^S^s are from *A. bisporus *(AbiTrz3 and AbiTrz4), *C. cinerea *(CciTrz2 and CciTrz3), *P. placenta *(PplTrz2 and PplTrz3), *L. bicolor *(LbiTrz2 and LbiTrz3), *S. punctatus *(SpuTrz3), *M. globosa *(MglTrz2), *Melampsora laricis-populina *(MlaTrz2) and *P. graminis *(PgrTrz2), as well as in *H. sapiens *(HsaTrz1) [9], *B. subtilis *(BsuTrz1) [55] and *E. coli *(EcoTrz1) [57]. See Table 1 for protein description. Alignment of eukaryotic tRNase Z^S^s is as described in the legend to Figure 2.

Like fungal tRNase Z^L^s, the lengths of predicted fungal tRNase Z^S^s are variable, ranging from 376 to 554 aa with an average size of ~442 aa. Sequence alignment of 15 selected representatives of fungal and non-fungal tRNase Z^S^s is presented in Figure 6. A list of all aligned fungal tRNase Z^S^s is provided in Additional file 4. Alignment revealed that like *B. subtilis *and human tRNase Z^S^s, fungal tRNase Z^S^s contain Motifs I-V and the PxKxRN, HEAT and HST loops (Figure 6). However, they display flexible arm diversity. A candidate flexible arm was also found in most of fungal tRNase Z^S^s. However, they exhibit considerable variation in amino acid sequence. Fungal tRNase Z^S^s can be grouped according to the presence or absence and the nature of sequences of the flexible arm. One group containing candidates from Basidiomycota species *A. bisporus *(AbiTrz4), *C. cinerea *(CciTrz3), *L. bicolor *(LbiTrz3) and *P. placenta *(PplTrz3) does not seem to contain the flexible arm, which is located between Motif III and Motif IV. Interestingly, all these species have two tRNase Z^S^s. The second group including candidates from Basidiomycota species *Agaricus bisporus *(AbiTrz3), *Coprinopsis cinerea *(CciTrz2), *Postia placenta *(PplTrz2) and *Laccaria bicolor *(LbiTrz2) lacks a recognizable GP motif but retain the Walker A-like motif. The third group including Basidiomycota species *Malassezia globosa *(MglTrz2), *Melampsora laricis-populina *(MlaTrz2) and *Puccinia graminis *(PgrTrz2), and chytrid species *Spizellomyces punctatus *(SpuTrz3) lacks both recognizable GP and Walker A-like motifs and is considerably longer than those of the second group. Moreover, the sequence similarity between the flexible arms of fungal tRNase Z^S^s and the ZiPD-type flexible arm is mostly confined to their C-terminal sequences.

## Discussion

### The phylogenetic distribution of tRNase Z in fungi

tRNase Z^L ^is widespread in fungi and the majority of fungal species appear to have a single tRNase Z^L^. This latter finding is somewhat unexpected given striking differences in the genome size, life cycle and morphology of species of fungi. The Pezizomycotina and Saccharomycotina belong to later diverging fungi. Their genomes vary considerably in size due to gene gain and loss events including tandem gene duplication, whole-genome duplication and extensive gene loss [37,38]. The Saccharomycotina genome sizes vary from ~9 (*Pichia pastoris*) to ~24 Mb (*Candida parapsilosis*), whereas the genome sizes of the Pezizomycotina fungi range from 23 (*Microsporum canis*) to 43 Mb (*N. crassa*). Furthermore, Pezizomycotina and Saccharomycotina fungi range in complexity from unicellular yeasts to filamentous molds. However, despite their remarkable differences in genome size, life cycle and morphology, the Pezizomycotina and Saccharomycotina fungi tend to contain only one tRNase Z^L^. These results indicate that the diversity of tRNase Z in fungi is not directly proportional to either the difference in genome size or the complexity of the life cycle and the morphology.

In contrast to tRNase Z^L^, tRNase Z^S ^has a limited phylogenetic distribution. The apparent lack of the tRNase Z^S ^gene in the genomes of Ascomycota species suggests that it has been deleted from the genomes of Ascomycota fungi. It is possible that tRNase Z^S ^existed before the divergence of the Ascomycota from the Basidiomycota, and was subsequently lost after the appearance of a novel structure (tRNase Z^L^). This is supported by the finding that tRNase Z^S ^is retained in the genomes of Basidiomycota, Chytridiomycota and Zygomycota fungi.

Most of Basidiomycota species examined contain one tRNase Z^S^. The fungal tRNase Z^S^s appear to be unique among all known tRNase Z^S^s in either lacking the flexible arm or having an atypical flexible arm (see discussion below). One explanation of existence of tRNase Z^S ^genes in Basidiomycota species is that these genes may represent pseudo-tRNase Z^S ^genes. Another explanation, which we favor, is that fungal tRNase Z^S^, at least some of them, may play a back-up or different role. Support for this hypothesis comes from recent studies of tRNase Z^S ^in *A. thaliana *and humans. In *A. thaliana*, one tRNase Z^S ^may represent a back-up for the nuclear tRNA 3'-end processing in case of dysfunction of nuclear-localized tRNase Z^L^, whereas the other plays a role in chloroplasts [13]. In human cells, tRNase Z^S ^is located in the cytosol and likely have substrates other than pre-tRNA [39].

### The flexible arms of candidate fungi tRNase Zs display remarkable diversity

An unexpected and striking result of this analysis is the diversity of the flexible arms within candidate fungal tRNase Zs, particularly tRNase Z^S^s. The most characteristic features of the typical flexible arm found in tRNase Zs are the GP- and Walker-A like motifs. A subset of fungal tRNase Z^L^s and all fungal tRNase Z^S^s appear to lack the GP-motif, and some fungal tRNase Z^S^s do not seem to have the Walker A-like motif. In the most extreme case, the flexible arm is missing in fungal tRNase Z^S^s. It is not yet understood why the flexible arms of fungal tRNase Zs display diversity in the primary sequence.

It is interesting to note that four Basidiomycota species contain two candidate tRNase Z^S^s, one of which lacks the flexible arm. It is likely that these two tRNase Z^S^s form heterodimers that would look like tRNase Z^L^, where only the N-terminal half has a flexible arm.

The apparent lack of the GP-motif in some fungal tRNase Z^L^s and all fungal tRNase Z^S^s that we have examined raises the question of whether this motif is absolutely required for substrate binding. Structural and biochemical evidence suggests that the GP-motif may not be essential for pre-tRNA binding. To date, the three-dimensional structures of tRNase Z^S^s from *B. subtilis*, *E. coli *and *T. maritima *have been solved by X-ray crystallography [34,40-43]. Remarkably, the flexible arms of *T. maritima *tRNase Z^S ^lacking the GP motif and the other two tRNase Z^S^s harboring the GP motif have very similar structures, composed of a compact globular domain and an extended two-stranded stalk, which extrude from the tRNase Z^S ^core. However, they have different lengths and globular domains. The globular domains at the end of the flexible arms of *B. subtilis *and *E. coli *tRNase Z^S^s are composed of two *α*-helices, two *β*-strands and one 3_10_-helix, whereas the counterpart in *T. maritima *tRNase Z^S ^consists of one very short *α*-helix, one long helix and one 3_10_-helix. The conserved GP-motif, particularly the proline residues, appears to add rigidity to two flexible arm helices since it is localized between them [42]. It would be interesting to know how the flexible arm lacking the GP-motif participates in substrate binding.

Recent biochemical studies have also suggested that the GP-motif may not be essential for substrate binding. Single alanine substitutions across the GP motif in *D. melanogaster *tRNase Z^L ^only moderately affect substrate binding. In contrast, substitution of a conserved leucine residue at the boundary of the globular domain and stalk with alanine almost completely abolishes substrate binding as the globular domain deletion [44]. Similarly, deletion of the GP motif in *B. subtilis *tRNase Z^S ^does not eliminate pre-tRNA binding but alters the cleavage specificity of the enzyme [45]. These results suggest that the GP motif may be important but not essential for substrate binding.

### The evolutionary relationship between tRNase Z^S ^and tRNase Z^L^

In eukaryotes, tRNase Z^L ^appears to take over tRNase Z^S ^in endonucleolytic 3'-end processing of pre-tRNAs, which raises the question of how it evolves. The protein sequence of tRNase Z^S ^is much more similar to the C-terminal half of tRNase Z^L ^than to the N-terminal half of tRNase Z^L^. Furthermore, the C-terminal half of tRNase Z^L ^retains all conserved motifs for proper catalytic function but has lost the flexible arm involved in substrate binding, whereas the N-terminal half has lost all active motifs but contains the flexible arm. These observations led to the proposal that tRNase Z^L ^has evolved from tRNase Z^S ^by gene duplication and subsequent sequence divergence [9]. To assess whether phylogenetic evidence exists that is consistent with this notion, we estimated phylogenetic relationships among fungal tRNase Zs by using a Bayesian phylogenetic method. The clustering of all fungal tRNase Z^S^s with representative bacterial tRNase Z^S^s support the notion that tRNase Z^L ^comes to eukaryotes through duplication of tRNase Z^S ^gene. Further evidence that the N-terminal half of tRNase Z^L ^is derived from an ancient tRNase Z^S ^comes from our findings that the N-terminal half of fungal tRNase Z^L ^contains candidate pseudo-motifs and that these pseudo-motifs are present in the same relative order as their original motifs appeared in tRNase Z^S^. These pseudo-motifs likely represent relics of original tRNase Z^S ^motifs that were inactivated during diversification of the eukaryotic tRNase Z^L ^gene.

The reason for the adoption of tRNase Z^L ^over tRNase Z^S ^in eukaryotes is unknown. One possibility is that eukaryotic cells may require more efficient tRNase Z enzymes. Support for this proposal comes from biochemical characterization of human tRNase Zs. In vitro characterization of recombinant human tRNase Zs have shown that tRNase Z^L ^cleaves pre-tRNA significantly more efficiently compared to tRNase Z^S ^[46]. Although strong structural evidence to support that tRNase Z^L ^evolved into a more efficient enzyme than tRNase Z^S ^is still lacking, it is interesting to note that tRNase Z^S ^and tRNase Z^L ^may have different processing center numbers which would make much difference in the efficiency of pre-tRNA 3'-end processing. Three-dimensional structures of three bacterial tRNase Z^S^s have revealed that the proteins form homodimers [34,40-43]. In particular, the crystal structure of *B. subtilis *tRNase Z^S ^in complex with tRNA shows that the dimer has two identical processing centers with two substrate binding and catalytic sites. In contrast, a molecular modeling study has suggested that both the N-terminal and C-terminal halves of human tRNase Z^L ^can fold into two distinct MBL domains with one domain containing a fully functionally catalytic site and the other containing a candidate substrate binding domain [3].

### Schizosaccharomyces fission yeasts have two tRNase Z^L^s, most likely targeted to the nucleus and mitochondria, respectively

*Schizosaccharomyces *fission yeasts including *S. pombe *appear to be unique among the Ascomycota in having two tRNase Z^L^s (tRNase Z^L1 ^and tRNase Z^L2^) that appear to be localized to the nucleus and mitochondria, respectively, as suggested in our previous study of tRNase Z^L ^in *S. pombe *[28]. Our fungal tRNase Z phylogeny shows that the two tRNase Z^L^s in fission yeasts may have arisen through gene duplication (Figure 1). Although the whole genome duplication found in the Saccharomycotina yeasts does not seem to occur in fission yeasts such as *S. pombe *[47], the tRNase Z^L ^gene could be duplicated by other mechanisms such as tandem and segmental gene duplication. The two tRNase Z^L^s in each *Schizosaccharomyces *species all have very limited homology with each other (20-23% identity and 31-33% similarity, see Additional file 5), indicating that these two proteins have diverged considerably from each other since their duplication. It is interesting to note that the tRNase Z^L ^gene could also be duplicated in non-fungal eukaryotic species *A. thaliana*. However, the two plant tRNase Z^L^s are highly related to each other (69% identity and 72% similarity).

Why do *Schizosaccharomyces *fission yeasts have two tRNase Z^L^s? In our previous study, it was found that the nuclear-targeted tRNase Z^L1 ^(SpoTrz1) is involved in nuclear pre-tRNA 3'-end processing in *S. pombe *[14]. Furthermore, its function can be compensated by either *S. cerevisia*e or human tRNase Z^L^. Although the role of mitochondrial-targeted tRNase Z^L2 ^(SpoTrz2) remains to be determined, it is likely that this protein plays an essential role in mitochondrial RNA processing [14]. Based on these results, it is possibly that the presence of two tRNase Z^L^s reflects that the nuclear and mitochondrial tRNA processing activities are associated with two different tRNase Z^L^s in *Schizosaccharomyces *fission yeasts. This may also hold true for wheat and potato since in these plants, enzymes involved in nuclear and mitochondrial tRNA 3'-end processing appear to be different [48]. However, it is important to note that the nuclear and organelle tRNase Z activities in the majority of organisms described to date seem to reside in the same enzyme.

## Conclusions

A survey of fungal databases shows that tRNase Z^L ^appears to be universally present in fungi, whereas the presence of tRNase Z^S ^is restricted to certain fungal phyla, indicative of the fundamental role of tRNase Z^L ^in eukaryotic tRNA biogenesis. The apparent lack of tRNase Z^S ^in the Ascomycota suggests that tRNase Z^S ^may have lost before divergence of the Ascomycota and the Basidiomycota. A striking aspect of the tRNase Z^L ^distribution is that there are two different tRNase Z^L^s in *Schizosaccharomyces *fission yeasts. These two tRNase Z^L^s are likely present in different cellular compartments, suggesting functional partitioning between these two proteins. Phylogenetic analysis suggests that tRNase Z^S ^is ancestral to tRNase Z^L ^and that tRNase Z^L ^gene duplications may have occurred in certain fungal taxa, including *Schizosaccharomyces *fission yeasts. Sequence analysis reveals that the domain architecture of tRNase Z^L^s is highly conserved among fungi and metazoa. A surprising result of sequence analysis is the sequence diversity in the putative flexible arm of candidate fungal tRNase Zs. Our analysis also reveals pseudo-motifs at the N-terminal halves of tRNase Z^L^s. These findings support the view that tRNase Z^L ^evolved through duplication and divergence of the tRNase Z^S ^gene.

## Methods

### Fungal genome database search and protein sequence analysis

To identify candidate tRNase Zs, we conducted BLAST and PSI-BLAST searches using the known tRNase Z protein sequences as queries against fungal genomes databases including the National Center for Biotechnology Information (NCBI; http://www.ncbi.nlm.nih.gov/sutils/genom_table.cgi?organism=fungi), the Broad Institute http://www.broadinstitute.org/science/data, the Joint Genome Institute http://genome.jgi-psf.org/pages/fungi/home.jsf, the Genome Center at Washington University http://genome.wustl.edu/ and the Universal Protein Resource http://www.uniprot.org. All candidate sequences were obtained by using the cut-off E-value of 0.01. All candidate proteins were subjected to validation, which was carried out by using a variety tests that evaluate the likelihood of annotation errors and the amino acid sequence conservation within and among taxonomic groups. First, confirmation of true candidate tRNase Zs was done by back-searching individual candidate protein sequence against the GenBank database. Second, the gene sequences for the predicted tRNase Zs were manually checked for possible sequence gaps. Third, multiple protein sequence alignment was used to identify candidate proteins that were discordant due to possible genomic sequencing errors and/or intron misprediction. In most cases, we changed the splicing pattern of candidate tRNase Z either using gene prediction programs Fgenesh http://linux1.softberry.com/berry.phtml?topic=fgenesh&group=programs&subgroup=gfind and Geneid http://genome.crg.es/geneid.html or manually to restore the high degree of sequence conservation. Multiple sequence alignment was performed by using Clustal W [49], and the resulting alignment was further manually examined and adjusted to improve the detection of conserved regions. The putative subcellular localization signals of tRNase Zs were predicted by using the programs MitoProt http://ihg2.helmholtz-muenchen.de/ihg/mitoprot.html and PSORT II http://psort.hgc.jp/.

### Phylogenetic analysis of fungal tRNase Zs

Full-length amino acid sequences of tRNase Zs from fungi and two bacteria, *B. subtilis *and *E. coli *were aligned by using Clustal W implemented in Mega 4.0 [50]. Conserved regions were selected and ambiguous aligned regions were removed by using the program Gblocks 0.91b [51]. tRNase Z^S^s from *B. subtilis *and *E. coli *were chosen as reference. The phylogenies were estimated by Bayesian inference with MrBayes 3.1.2 [52] using a mixture of the fixed amino acid models and the gamma distribution. Statistical confidence was assessed by using Markov Chain Monte Carlo (MCMC) sampling approaches. Four simultaneous Markov chains were run for one million generations sampling every 1,000 generation in two replicate runs. The first 250 trees were discarded as burn-in and the convergence of the chains was evaluated using AWTY implemented in MrBayes 3.1.2 [53].

## List of abbreviations

pre-tRNA: tRNA precursor; tRNase Z: tRNA 3' endonuclease; tRNase Z^S^: the short form of tRNase Z; tRNase Z^L^: the long form of tRNase Z; aa: amino acid; MBL: metallo-*β*-lactamase; NLS: nuclear localization signal; MTS: mitochondrial targeting signal; no: number; kDa: kiloDaltons

## Authors' contributions

WZ conducted online database searches and carried out the sequence alignment. SL constructed the phylogenetic tree. WZ, HY and YH analyzed the data. YH wrote the manuscript. All authors read and approved the final manuscript.

## Supplementary Material

Additional file 1 **Distribution of candidate fungal tRNase Zs**. ^a^Abbreviations for species names are indicated in the parentheses. ^b^The number of amino acids in fungal tRNase Zs. ^c^also known as *Histoplasma capsulatum*. ^d^also known as *Blastomyces dermatitidis*. ^e^also known as *Gibberella zeae *^f^also known as *Sporotrichum thermophile *^g^also known as *Fusarium solani *^h^also known as *Stagonospora nodorum *^i^also known as *Filobasidiella neoformans *ND denotes the sequence could not be predicted correctly likely due to sequencing errors. *Indicates that mispredicted sequences obtained from the databases have been corrected. Click here for file

Additional file 2 **Putative N-terminal mitochondrial targeting signals in candidate fungal tRNase Zs**. The accession numbers for the proteins are listed in Additional file 1. The numbers refer to amino acid position starting from the N-terminus. ^#^SpoTrz2 (SPBC3D6.03C) is localized to the mitochondria [28]. Click here for file

Additional file 3 **Alignment of candidate fungal tRNase Z^L^s**. Similar or identical amino acid residues are shaded as described in the legend to Figure 2. The conserved motifs are labeled according to references [30,31,44]. Click here for file

Additional file 4 **Alignment of candidate fungal tRNase Z^S^s**. Similar or identical amino acid residues are shaded as described in the legend to Figure 2. The conserved motifs are labeled according to references [30,31,44]. Click here for file

Additional file 5 **Pairwise sequence comparisons of tRNase Z^L^s from *Schizosaccharomyces *species**. The accession numbers for proteins are listed in Additional file 1. The pairwise percent identity (I) and percent similarity (S) between tRNase Z^L^s from *Schizosaccharomyces *species were calculated using the Clustal W program [49]. Click here for file
