# Supplementary material for: Identification and analysis of candidate fungal tRNA 3'-end processing endonucleases tRNase Zs, homologs of the putative prostate cancer susceptibility protein ELAC2
Source: BMC Evol Biol. 2010 Sep 6;10:272. doi: 10.1186/1471-2148-10-272 (PMC2942849; doi:10.1186/1471-2148-10-272)
Supplement: Additional file 1 — Distribution of candidate fungal tRNase Zs. aAbbreviations for species names are indicated in the parentheses. bThe number of amino acids in fungal tRNase Zs. calso known as Histoplasma capsulatum. dalso known as Blastomyces dermatitidis. ealso known as Gibberella zeae falso known as Sporotrichum thermophile galso known as Fusarium solani halso known as Stagonospora nodorum ialso known as Filobasidiella neoformans ND denotes the sequence could not be predicted correctly likely due to sequencing errors. *Indicates that mispredicted sequences obtained from the databases have been corrected. [file 1471-2148-10-272-S1.DOC]

| Species^a^ | Protein name | Form | Accession number/Locus | Database | No. aa^b^ |
| --- | --- | --- | --- | --- | --- |
| Ascomycota (Pezizomycotina) |  |  |  |  |  |
| Ajellomyces capsulatus (Aca)^c^ | AcaTrz1 | tRNase Z^L^ | HCAG_02192.1 | Broad | 1131 |
| Ajellomyces dermatitidis (Ade)^d^ | AdeTrz1 | tRNase Z^L^ | BDBG_07583.1 | Broad | 1140* |
| Alternaria brassicicola (Abr) | AbrTrz1 | tRNase Z^L^ | 1559 | JGI | 1010 |
| Aspergillus carbonarius (Acr) | AcrTrz1 | tRNase Z^L^ | 10545 | JGI | 1062 |
| Aspergillus clavatus (Acl) | AclTrz1 | tRNase Z^L^ | ACLA_019120 | Broad | 1080 |
| Aspergillus flavus (Afl) | AflTrz1 | tRNase Z^L^ | AFL2G_01018.2 | Broad | 1080 |
| Aspergillus fumigatus (Afu) | AfuTrz1 | tRNase Z^L^ | Afu1g16470 | Broad | 1081 |
| Aspergillus nidulans (Ani) | AniTrz1 | tRNase Z^L^ | ANID_11892.1 | Broad | 1083 |
| Aspergillus niger (Asp) | AspTrz1 | tRNase Z^L^ | 35699 | JGI | 1066 |
| Aspergillus terreus (Ate) | AteTrz1 | tRNase Z^L^ | ATEG_05160.1 | Broad | 1076 |
| Aspergillus oryzae (Aor) | AorTrz1 | tRNase Z^L^ | AO090005001063 | Broad | 1080* |
| Arthroderma benhamiae (Abe) | AbeTrz1 | tRNase Z^L^ | EFE30974 | NCBI | 980* |
| Chaetomium globosum (Cgl) | CglTrz1 | tRNase Z^L^ | CHGG_03393.1 | Broad | 957 |
| Coccidioides posadasii (Cpo) | CpoTrz1 | tRNase Z^L^ | EER26908 | NCBI | 963 |
| Coccidioides immitis (Cim) | CimTrz1 | tRNase Z^L^ | CIHG_00067.1 | Broad | 962* |
| Cochliobolus heterostrophus (Che) | CheTrz1 | tRNase Z^L^ | 29905 | JGI | 1010 |
| Fusarium graminearum (Fgr)^e^ | FgrTrz1 | tRNase Z^L^ | FGSG_06635.3 | Broad | 840 |
| Fusarium oxysporum (Fox) | FoxTrz1 | tRNase Z^L^ | FOXG_02309.2 | Broad | 836 |
| Fusarium verticillioides (Fve) | FveTrz1 | tRNase Z^L^ | FVEG_05485.3 | Broad | 839 |
| Magnaporthe grisea (Mgr) | MgrTrz1 | tRNase Z^L^ | MGG_05303.6 | Broad | 959 |
| Microsporum canis (Mca) | McaTrz1 | tRNase Z^L^ | MCYG_00015.1 | Broad | 982* |
| Microsporum gypseum (Mgy) | MgyTrz1 | tRNase Z^L^ | MGYG_07210.1 | Broad | 975* |
| Myceliophthora thermophila (Mth)^f^ | MthTrz1 | tRNase Z^L^ | 48907 | JGI | 864 |
| Mycosphaerella fijiensis (Mfi) | MfiTrz1 | tRNase Z^L^ | 42998 | JGI | 976 |
| Mycosphaerella graminicola (Myc) | MycTrz1 | tRNase Z^L^ | 55565 | JGI | 930 |
| Nectria haematococca (Nha)^g^ | NhaTrz1 | tRNase Z^L^ | EEU46659 | NCBI | 836 |
| Neosartorya fischeri (Nfi) | NfiTrz1 | tRNase Z^L^ | NFIA_008970 | Broad | 1077 |
| Neurospora crassa (Ncr) | NcrTrz1 | tRNase Z^L^ | NCU00232.4 | Broad | 1099 |
| Neurospora discreta (Ndi) | NdiTrz1 | tRNase Z^L^ | 125997 | JGI | 1099 |
| Neurospora tetrasperma (Nte) | NteTrz1 | tRNase Z^L^ | 113795 | JGI | 1099 |
| Paracoccidioides brasiliensis (Pbr) | PbrTrz1 | tRNase Z^L^ | PABG_06100.1 | Broad | 1099 |
| Penicillium chrysogenum (Pch) | PchTrz1 | tRNase Z^L^ | XP_002569270 | NCBI | 1042 |
| Penicillium marneffei (Pma) | PmaTrz1 | tRNase Z^L^ | XP_002146096 | NCBI | 1010* |
| Phaeosphaeria nodorum (Pno)^h^ | PnoTrz1 | tRNase Z^L^ | SNOG_02316.1 | Broad | 1008* |
| Podospora anserina (Pan) | PanTrz1 | tRNase Z^L^ | XP_001907744 | NCBI | 927* |
| Pyrenophora tritici-repentis (Ptr) | PtrTrz1 | tRNase Z^L^ | XP_001940780 | NCBI | 988* |
| Sclerotinia sclerotiorum (Ssc) | SscTrz1 | tRNase Z^L^ | XP_001586541 | NCBI | 832 |
| Sordaria macrospora (Sma) | SmaTrz1 | tRNase Z^L^ | CBI57280 | NCBI | 1110 |
| Talaromyces stipitatus (Tst) | TstTrz1 | tRNase Z^L^ | XP_002478395 | NCBI | 1016* |
| Thielavia terrestris (Tte) | TteTrz1 | tRNase Z^L^ | 59748 | JGI | 852 |
| Trichoderma atroviride (Tat) | TatTrz1 | tRNase Z^L^ | 147574 | JGI | 847 |
| Trichoderma reesei (Tre) | TreTrz1 | tRNase Z^L^ | 61701 | JGI | 855 |
| Trichoderma virens (Tvi) | TviTrz1 | tRNase Z^L^ | 38340 | JGI | 845 |
| Trichophyton rubrum (Tru) | TruTrz1 | tRNase Z^L^ | TERG_08138.2 | Broad | 978 |
| Uncinocarpus reesii (Ure) | UreTrz1 | tRNase Z^L^ | EEP78058 | NCBI | 966 |
| Ascomycota (Saccharomycotina) |  |  |  |  |  |
| Ashbya gossypii (Ago) | AgoTrz1 | tRNase Z^L^ | NP_984308 | NCBI | 821 |
| Candida albicans (Cal) | CalTrz1 | tRNase Z^L^ | XP_717703 | NCBI | 857 |
| Candida dubliniensis (Cdu) | CduTrz1 | tRNase Z^L^ | XP_002418986 | NCBI | 857 |
| Candida glabrata (Can) | CanTrz1 | tRNase Z^L^ | XP_446456 | NCBI | 826 |
| Candida guilliermondii (Cgu) | CguTrz1 | tRNase Z^L^ | PGUG_02613.1 | Broad | 870 |
| Candida parapsilosis (Cpa) | CpaTrz1 | tRNase Z^L^ | CPAG_03878 | Broad | 836 |
| Candida tropicalis (Ctr) | CtrTrz1 | tRNase Z^L^ | EER30146 | NCBI | 858 |
| Clavispora lusitaniae (Clu) | CluTrz1 | tRNase Z^L^ | EEQ36475 | NCBI | 860 |
| Debaryomyces hansenii (Dha) | DhaTrz1 | tRNase Z^L^ | XP_459554 | NCBI | 875 |
| Kluyveromyces lactis (Kla) | KlaTrz1 | tRNase Z^L^ | XP_455829 | NCBI | 822 |
| Lachancea thermotolerans (Lth) | LthTrz1 | tRNase Z^L^ | XP_002554954 | NCBI | 815 |
| Pichia guilliermondii (Pgu) | PguTrz1 | tRNase Z^L^ | EDK38515 | NCBI | 870 |
| Pichia pastoris (Ppa) | PpaTrz1 | tRNase Z^L^ | XP_002493212 | NCBI | 816 |
| Pichia stipitis (Pst) | PstTrz1 | tRNase Z^L^ | XP_001382363 | NCBI | 871 |
| Saccharomyces cerevisiae (Sce) | SceTrz1 | tRNase Z^L^ | NP_013005 | NCBI | 838 |
| Vanderwaltozyma polyspora (Vpo) | VpoTrz1 | tRNase Z^L^ | XP_001643490 | NCBI | 826 |
| Yarrowia lipolytica (Yli) | YliTrz1 | tRNase Z^L^ | XP_500027 | NCBI | 815 |
| Zygosaccharomyces rouxii (Zro) | ZroTrz1 | tRNase Z^L^ | XP_002494867 | NCBI | 812 |
| Ascomycota (Taphrinomycotina ) |  |  |  |  |  |
| Schizosaccharomyces japonicus (Sja) | SjaTrz1 | tRNase Z^L1^ | SJAG_00997.2 | Broad | 755 |
| Schizosaccharomyces japonicus (Sja) | SjaTrz2 | tRNase Z^L2^ | SJAG_04885.2 | Broad | 648 |
| Schizosaccharomyces octosporus (Soc) | SocTrz1 | tRNase Z^L1^ | SOCG_01925.3 | Broad | 788 |
| Schizosaccharomyces octosporus (Soc) | SocTrz2 | tRNase Z^L2^ | SOCG_03403.3 | Broad | 678 |
| Schizosaccharomyces pombe (Spo) | SpoTrz1 | tRNase Z^L1^ | SPAC1D4.10 | Broad | 809 |
| Schizosaccharomyces pombe (Spo) | SpoTrz2 | tRNase Z^L2^ | SPBC3D6.03C | Broad | 678 |
| Schizosaccharomyces cryophobus (Scr) | ScrTrz1 | tRNase Z^L1^ | SPOG_04087.2 | Broad | 789 |
| Schizosaccharomyces cryophobus (Scr) | ScrTrz2 | tRNase Z^L2^ | SPOG_04337.2 | Broad | 680 |
| Basidiomycota (Agaricomycotina) |  |  |  |  |  |
| Agaricus bisporus (Abi) | AbiTrz1 | tRNase Z^L^ | 149626 | JGI | 899 |
| Agaricus bisporus (Abi) | AbiTrz2 | tRNase Z^L^ | 128068 | JGI | 947* |
| Agaricus bisporus (Abi) | AbiTrz3 | tRNase Z^S^ | 106812 | JGI | 376 |
| Agaricus bisporus (Abi) | AbiTrz4 | tRNase Z^S^ | 115453 | JGI | 469 |
| Coprinopsis cinerea (Cci) | CciTrz1 | tRNase Z^L^ | CC1G_14814.2 | Broad | 967* |
| Coprinopsis cinerea (Cci) | CciTrz2 | tRNase Z^S^ | CC1G_14603.2 | Broad | 390 |
| Coprinopsis cinerea (Cci) | CciTrz3 | tRNase Z^S^ | CC1G_03806.2 | Broad | 544 |
| Cryptococcus neoformans (Cne)^i^ | CneTrz1 | tRNase Z^L^ | CNBG_1589.2 | Broad | 1035 |
| Cryptococcus neoformans (Cne)^i^ | CneTrz2 | tRNase Z^S^ | CNBG_5350.2 | Broad | 554* |
| Heterobasidion annosum (Han) | HanTrz1 | tRNase Z^L^ | 66321 | JGI | 901 |
| Heterobasidion annosum (Han) | HanTrz2 | tRNase Z^S^ | 123561 | JGI | 382 |
| Laccaria bicolor (Lbi) | LbiTrz1 | tRNase Z^L^ | XP_001875096 | NCBI | 888 |
| Laccaria bicolor (Lbi) | LbiTrz2 | tRNase Z^S^ | XP_001876619 | NCBI | 406 |
| Laccaria bicolor (Lbi) | LbiTrz3 | tRNase Z^S^ | [XP_001874963](http://www.ncbi.nlm.nih.gov/entrez/query.fcgi?cmd=Retrieve&db=Protein&list_uids=170087480&dopt=GenPept&RID=PRACBNTF01S&log$=protalign&blast_rank=2) | NCBI | 476 |
| Phanerochaete chrysosporium (Pha) | PhaTrz1 | tRNase Z^L^ | 3652 | JGI | 992* |
| Phanerochaete chrysosporium (Pha) | PhaTrz2 | tRNase Z^S^ | 1441 | JGI | 386* |
| Pleurotus ostreatus (Pos) | PosTrz1 | tRNase Z^L^ | 62050 | JGI | 903 |
| Pleurotus ostreatus (Pos) | PosTrz2 | tRNase Z^S^ | 50330 | JGI | 388* |
| Postia placenta (Ppl) | PplTrz1 | tRNase Z^L^ | 127047 | JGI | 1035* |
| Postia placenta (Ppl) | PplTrz2 | tRNase Z^S^ | 94043 | JGI | 386 |
| Postia placenta (Ppl) | PplTrz3 | tRNase Z^S^ | 92595 | JGI | 483* |
| Schizophyllum commune (Sco) | ScoTrz1 | tRNase Z^L^ | 76260 | JGI | 897 |
| Schizophyllum commune (Sco) | ScoTrz2 | tRNase Z^S^ | 12586 | JGI | 382 |
| Serpula lacrymans (Sla) | SlaTrz1 | tRNase Z^L^ | 412876 | JGI | 907 |
| Serpula lacrymans (Sla) | SlaTrz2 | tRNase Z^S^ | 360421 | JGI | 394 |
| Tremella mesenterica (Tme) | TmeTrz1 | tRNase Z^L^ | 70321 | JGI | 1016 |
| Tremella mesenterica (Tme) | TmeTrz2 | tRNase Z^S^ | 74522 | JGI | 541* |
| Basidiomycota (Puccciniomycotina) |  |  |  |  |  |
| Melampsora laricis-populina (Mla) | MlaTrz1 | tRNase Z^L^ | 90551 | JGI | 910* |
| Melampsora laricis-populina (Mla) | MlaTrz2 | tRNase Z^S^ | 111950 | JGI | 473* |
| Puccinia graminis (Pgr) | PgrTrz1 | tRNase Z^L^ | PGTG_11198.2 | Broad | 854 |
| Puccinia graminis (Pgr) | PgrTrz2 | tRNase Z^S^ | PGTG_13150.2 | Broad | 498 |
| Basidiomycota (Ustilaginomycotina) |  |  |  |  |  |
| Malassezia globosa (Mgl) | MglTrz1 | tRNase Z^L^ | XP_001729151 | NCBI | 1109 |
| Malassezia globosa (Mgl) | MglTrz2 | tRNase Z^S^ | EDP42443 | NCBI | 484* |
| Chytridiomycota |  |  |  |  |  |
| Allomyces macrogynus (Ama) | AmaTrz1 | tRNase Z^L^ | AMAG_05512.1 | Broad | 911* |
| Allomyces macrogynus (Ama) | AmaTrz2 | tRNase Z^L^ | AMAG_01321.1 | Broad | ND |
| Batrachochytrium dendrobatidis (Bde) | BdeTrz1 | tRNase Z^L^ | BDEG_00488.1 | Broad | 749 |
| Spizellomyces punctatus (Spu) | SpuTrz1 | tRNase Z^L^ | SPPG_00513.2 | Broad | 799* |
| Spizellomyces punctatus (Spu) | SpuTrz2 | tRNase Z^L^ | SPPG_04657.2 | Broad | 920 |
| Spizellomyces punctatus (Spu) | SpuTrz3 | tRNase Z^S^ | SPPG_06028.2 | Broad | 395 |
