# Supplementary material for: Identification and analysis of candidate fungal tRNA 3'-end processing endonucleases tRNase Zs, homologs of the putative prostate cancer susceptibility protein ELAC2
Source: BMC Evol Biol. 2010 Sep 6;10:272. doi: 10.1186/1471-2148-10-272 (PMC2942849; doi:10.1186/1471-2148-10-272)
Supplement: Additional file 2 — Putative N-terminal mitochondrial targeting signals in candidate fungal tRNase Zs. The accession numbers for the proteins are listed in Additional file 1. The numbers refer to amino acid position starting from the N-terminus. #SpoTrz2 (SPBC3D6.03C) is localized to the mitochondria [28]. [file 1471-2148-10-272-S2.DOC]

Additional data file 2. Putative N-terminal mitochondrial targeting signals in candidate fungal tRNase Zs

| Protein name | Predicted MTS | Probability |
| --- | --- | --- |
| SjaTrz2 | MFSVSRRQLLHSFKCLRLALRNY (23) | 0.98 |
| CciTrz2 | MAAVVQKRSLLQSLGITFLGTASAQPSSTRN (31) | 0.87 |
| PosTrz2 | MSRPPITSASITFLGTASAQPSSTRNHSSLALRL (34) | 0.76 |
| LbiTrz3 | MLFLPQRILRIRPINSCRRPPRRASSHIRYLDNRK (35) | 0.99 |
| SlaTrz2 | MSAGRRVFSSLNITFLGTASAQPSSTRNHSSLALRL (36) | 0.90 |
| ScrTrz2 | MKNLIYFKLFPLRGKNPITKLGHPLLNKLGTTVQLRR (37) | 0.97 |
| SocTrz2 | MKNIIHNKLCTLYKKTPKIMLNHALPSKVGTFVQLRW (37) | 0.71 |
| SpoTrz2^#^ | MKASLLVPRRALLFGQLLPPKYSWYSVKRWQSQLTFRN (38) | 0.99 |
| PbrTrz1 | MAISSLNPVSPHHTLHPLKSPSPFFILLRPSQCNTLSGFRT (41) | 0.91 |
| UmaTrz1 | MWWLSPTIVKLFCSSSGTTPLSSVCQLRVSSRAFATSAALS (41) | 0.96 |
| MgrTrz1 | MNIAQRTVHWVTGALKSPPRVQRLSIGNKHLLQIRYYKVYRRQ (43) | 0.98 |
| NcrTrz1 | MHYKKLAVAAASKRIRTVLPTRISTTTSSFLSLRRCLSTRDFTRSPESS (49) | 0.94 |
| NdiTrz1 | MHYKKLAVAAASKRIRTVLPTRITTTTSSLPSLSRCLSTRDFTASPDSS (49) | 0.96 |
| NteTrz1 | MHYKKLAVAAASKRIRTVLPTRIFTTTSSLLSLRRCLSTRDFTGSPESS (49) | 0.95 |
| LbiTrz2 | MTCYRRLIQIANVFGRRRTMSSSSASRQSPIGSLSATFLGTASAQPSSTRNHSALALRV(59) | 1.00 |
| SmaTrz1 | MHYKKLAVAAASKRIRTVLPTRISTLTTTTTSSLLSLRRCLSTRGFTPSSNSSADPSTFAKLRPRRVPNSASNPPSNPRA (81) | 0.94 |
| CglTrz1 | MYATAKANIVRVSCKLQKVPTPSLPLHLPSSPPAFFLFQSRSFRTFAKLQATNPSPQLCQSTAAIFPKSLFPGSTPRPIESSRPRAPRFVLVPSRRFQGPHPAITILSRP (110) | 0.99 |
