# Supplementary material for: Identification and analysis of candidate fungal tRNA 3'-end processing endonucleases tRNase Zs, homologs of the putative prostate cancer susceptibility protein ELAC2
Source: BMC Evol Biol. 2010 Sep 6;10:272. doi: 10.1186/1471-2148-10-272 (PMC2942849; doi:10.1186/1471-2148-10-272)
Supplement: Additional file 3 — Alignment of candidate fungal tRNase ZLs. Similar or identical amino acid residues are shaded as described in the legend to Figure 2. The conserved motifs are labeled according to references [30,31,44]. [file 1471-2148-10-272-S3.DOC]

**Additional data file 3. Alignment of candidate fungal tRNase Z^L^s**

**AbeTrz1 (1) --------------------------------------------------------------------------------------------------------------**

**TruTrz1 (1) --------------------------------------------------------------------------------------------------------------**

**MgyTrz1 (1) --------------------------------------------------------------------------------------------------------------**

**McaTrz1 (1) --------------------------------------------------------------------------------------------------------------**

**CimTrz1 (1) --------------------------------------------------------------------------------------------------------------**

**CpoTrz1 (1) --------------------------------------------------------------------------------------------------------------**

**UreTrz1 (1) --------------------------------------------------------------------------------------------------------------**

**AcaTrz1 (1) --------------------------------------------------------------------------------------------------------------**

**AdeTrz1 (1) --------------------------------------------------------------------------------------------------------------**

**PbrTrz1 (1) --------------------------------------------------------------------------------------------------------------**

**AclTrz1 (1) --------------------------------------------------------------------------------------------------------------**

**AfuTrz1 (1) --------------------------------------------------------------------------------------------------------------**

**NfiTrz1 (1) --------------------------------------------------------------------------------------------------------------**

**AcrTrz1 (1) --------------------------------------------------------------------------------------------------------------**

**AspTrz1 (1) --------------------------------------------------------------------------------------------------------------**

**AflTrz1 (1) --------------------------------------------------------------------------------------------------------------**

**AorTrz1 (1) --------------------------------------------------------------------------------------------------------------**

**AteTrz1 (1) --------------------------------------------------------------------------------------------------------------**

**PchTrz1 (1) --------------------------------------------------------------------------------------------------------------**

**AniTrz1 (1) --------------------------------------------------------------------------------------------------------------**

**PmaTrz1 (1) --------------------------------------------------------------------------------------------------------------**

**TstTrz1 (1) --------------------------------------------------------------------------------------------------------------**

**AbrTrz1 (1) --------------------------------------------------------------------------------------------------------------**

**CheTrz1 (1) --------------------------------------------------------------------------------------------------------------**

**PtrTrz1 (1) --------------------------------------------------------------------------------------------------------------**

**PnoTrz1 (1) --------------------------------------------------------------------------------------------------------------**

**MfiTrz1 (1) --------------------------------------------------------------------------------------------------------------**

**MycTrz1 (1) --------------------------------------------------------------------------------------------------------------**

**SscTrz1 (1) --------------------------------------------------------------------------------------------------------------**

**CglTrz1 (1) ---------------------------------------------------------------------------------------------MYATAKANIVRVSCKLQ**

**MthTrz1 (1) --------------------------------------------------------------------------------------------------------------**

**TteTrz1 (1) --------------------------------------------------------------------------------------------------------------**

**PanTrz1 (1) --------------------------------------------------------------------------------------------------------------**

**NcrTrz1 (1) MHYKKLAVAAASKRIRTVLPTRIS----TTTSSFLSLRRCLSTRDFTRSPESSVDPSVFTSSRIRRVPDSVSKPRTTPFRNL--------RPAKPTPRLAVKPPSKDRPP**

**NteTrz1 (1) MHYKKLAVAAASKRIRTVLPTRIF----TTTSSLLSLRRCLSTRDFTGSPESSVDPSVFTSSRIRRVPDSVSNPRTTPSRNL--------RPAKPTTRLAVKPPSKDRPS**

**NdiTrz1 (1) MHYKKLAVAAASKRIRTVLPTRIT----TTTSSLPSLSRCLSTRDFTASPDSSTDPAVFTSSRIRRVPDSASNPKATPFRNP--------RPAKPTTRLDVKPPSKDRPS**

**SmaTrz1 (1) MHYKKLAVAAASKRIRTVLPTRISTLTTTTTSSLLSLRRCLSTRGFTPSSNSSADPSTFAKLRPRRVPNSASNPPSNPRASPSRDPRPYPRPAKPTPSLVVDPPKKSGSS**

**MgrTrz1 (1) ---------------------------------------------------------------------------------------------------------MNIAQ**

**FgrTrz1 (1) --------------------------------------------------------------------------------------------------------------**

**FoxTrz1 (1) --------------------------------------------------------------------------------------------------------------**

**FveTrz1 (1) --------------------------------------------------------------------------------------------------------------**

**NhaTrz1 (1) --------------------------------------------------------------------------------------------------------------**

**TatTrz1 (1) --------------------------------------------------------------------------------------------------------------**

**TviTrz1 (1) --------------------------------------------------------------------------------------------------------------**

**TreTrz1 (1) --------------------------------------------------------------------------------------------------------------**

**AgoTrz1 (1) --------------------------------------------------------------------------------------------------------------**

**CanTrz1 (1) --------------------------------------------------------------------------------------------------------------**

**SceTrz1 (1) --------------------------------------------------------------------------------------------------------------**

**ZroTrz1 (1) --------------------------------------------------------------------------------------------------------------**

**VpoTrz1 (1) --------------------------------------------------------------------------------------------------------------**

**LthTrz1 (1) --------------------------------------------------------------------------------------------------------------**

**KlaTrz1 (1) --------------------------------------------------------------------------------------------------------------**

**PpaTrz1 (1) --------------------------------------------------------------------------------------------------------------**

**CalTrz1 (1) --------------------------------------------------------------------------------------------------------------**

**CduTrz1 (1) --------------------------------------------------------------------------------------------------------------**

**CtrTrz1 (1) --------------------------------------------------------------------------------------------------------------**

**CpaTrz1 (1) --------------------------------------------------------------------------------------------------------------**

**CguTrz1 (1) --------------------------------------------------------------------------------------------------------------**

**PguTrz1 (1) --------------------------------------------------------------------------------------------------------------**

**DhaTrz1 (1) --------------------------------------------------------------------------------------------------------------**

**PstTrz1 (1) --------------------------------------------------------------------------------------------------------------**

**CluTrz1 (1) --------------------------------------------------------------------------------------------------------------**

**YliTrz1 (1) --------------------------------------------------------------------------------------------------------------**

**AmaTrz1 (1) --------------------------------------------------------------------------------------------------------------**

**BdeTrz1 (1) --------------------------------------------------------------------------------------------------------------**

**SpuTrz2 (1) --------------------------------------------------------------------------------------------------------------**

**SpuTrz1 (1) --------------------------------------------------------------------------------------------------------------**

**ScrTrz1 (1) --------------------------------------------------------------------------------------------------------------**

**SocTrz1 (1) --------------------------------------------------------------------------------------------------------------**

**SpoTrz1 (1) --------------------------------------------------------------------------------------------------------------**

**SjaTrz1 (1) --------------------------------------------------------------------------------------------------------------**

**ScrTrz2 (1) --------------------------------------------------------------------------------------------------------------**

**SocTrz2 (1) --------------------------------------------------------------------------------------------------------------**

**SpoTrz2 (1) --------------------------------------------------------------------------------------------------------------**

**SjaTrz2 (1) --------------------------------------------------------------------------------------------------------------**

**CneTrz1 (1) --------------------------------------------------------------------------------------------------------------**

**TmeTrz1 (1) --------------------------------------------------------------------------------------------------------------**

**AbiTrz1 (1) --------------------------------------------------------------------------------------------------------------**

**CciTrz1 (1) --------------------------------------------------------------------------------------------------------------**

**ScoTrz1 (1) --------------------------------------------------------------------------------------------------------------**

**LbiTrz1 (1) --------------------------------------------------------------------------------------------------------------**

**PosTrz1 (1) --------------------------------------------------------------------------------------------------------------**

**SlaTrz1 (1) --------------------------------------------------------------------------------------------------------------**

**HanTrz1 (1) --------------------------------------------------------------------------------------------------------------**

**PplTrz1 (1) --------------------------------------------------------------------------------------------------------------**

**PhaTrz1 (1) --------------------------------------------------------------------------------------------------------------**

**AbiTrz2 (1) --------------------------------------------------------------------------------------------------------------**

**MglTrz1 (1) --------------------------------------------------------------------------------------------------------------**

**MlaTrz1 (1) --------------------------------------------------------------------------------------------------------------**

**PgrTrz1 (1) --------------------------------------------------------------------------------------------------------------**

**AbeTrz1 (1) -----------------------------------------------------------------------------------------------MKSFIQFVTAPTSDT**

**TruTrz1 (1) -----------------------------------------------------------------------------------------------MKSFIQFVTAPTSDT**

**MgyTrz1 (1) -----------------------------------------------------------------------------------------------MKSFIQFVTAPTSDT**

**McaTrz1 (1) -----------------------------------------------------------------------------------------------MKSFIQFVTAPTSDT**

**CimTrz1 (1) -----------------------------------------------------------------------------------------------MKTFIQFVTTPAADT**

**CpoTrz1 (1) -----------------------------------------------------------------------------------------------MKTFIQFVTTPTADT**

**UreTrz1 (1) -----------------------------------------------------------------------------------------------MKTFIQFITTPTVDT**

**AcaTrz1 (1) -----------------------------------------------------------------------------------------------MKFFFEFITTPTADT**

**AdeTrz1 (1) -----------------------------------------------------------------------------------------------MIFFCEFITTPTADT**

**PbrTrz1 (1) -----------------------------------------------------------------------------------------------MKFYFEFITTPTADT**

**AclTrz1 (1) -----------------------------------------------------------------------------------------------MKFYYQVLTTPTADT**

**AfuTrz1 (1) -----------------------------------------------------------------------------------------------MKFYYQVLTTPTADT**

**NfiTrz1 (1) -----------------------------------------------------------------------------------------------MKFYYQVLTTPTADT**

**AcrTrz1 (1) -----------------------------------------------------------------------------------------------MKSYYEVLTTPTADT**

**AspTrz1 (1) -----------------------------------------------------------------------------------------------MKFYYEILTTPTADT**

**AflTrz1 (1) -----------------------------------------------------------------------------------------------MKFFYQVITTPTADT**

**AorTrz1 (1) -----------------------------------------------------------------------------------------------MKFFYQVITTPTADT**

**AteTrz1 (1) -----------------------------------------------------------------------------------------------MKFYFQVVTTPTADT**

**PchTrz1 (1) -----------------------------------------------------------------------------------------------MKFFYQVITTPTADT**

**AniTrz1 (1) -----------------------------------------------------------------------------------------------MKFYYQILTTPTADT**

**PmaTrz1 (1) -----------------------------------------------------------------------------------------------MKFSYQVLSTPTADS**

**TstTrz1 (1) -----------------------------------------------------------------------------------------------MKFFYQVLSTPTADS**

**AbrTrz1 (1) -----------------------------------------------------------------------------------------------MSCSVEIITTPTADT**

**CheTrz1 (1) -----------------------------------------------------------------------------------------------MSCSVEIITTPTADS**

**PtrTrz1 (1) -----------------------------------------------------------------------------------------------MSHGVEIITTPTGDT**

**PnoTrz1 (1) -----------------------------------------------------------------------------------------------MSSSIEILTTPTSDT**

**MfiTrz1 (1) -----------------------------------------------------------------------------------------------MKSHVQVLTTPTVDS**

**MycTrz1 (1) -----------------------------------------------------------------------------------------------MRSFVQVLTTPTADT**

**SscTrz1 (1) -----------------------------------------------------------------------------------------------MRSYIQFLTTPTVDT**

**CglTrz1 (18) KVPTPSLPLHLPSSPPAFFLFQSRSFRTFAKLQATNPSPQLCQSTAAIFPKSLFPGSTPRPIESSRPRAPRFVLVPSRRFQGPHPAITILSRPTGMLCYVQILSTPTADT**

**MthTrz1 (1) -----------------------------------------------------------------------------------------------MLSYVQIVSTPTADT**

**TteTrz1 (1) -----------------------------------------------------------------------------------------------MLAYVQIVSVPSADT**

**PanTrz1 (1) -----------------------------------------------------------------------------------------------MTSYTQVVTTPTADT**

**NcrTrz1 (99) KSKIPDSVSKTVARELLRNRYLFAEYSLFSPQRAFQKWKNHGLLKHTKIPEDFFSGRIPIKLYPYQVDGPRFILASPAPEQKEAPPLPAKPEFGAMRSHVQIVAAPTADT**

**NteTrz1 (99) KSKIPDSVSKTVARELLRNRYLFSEYSLFSPQRAFQKWKNHGLLKHTKIPEDFFSGRIPIKLYPYQVDGPRFILASPAPEQKEALPPPAKPEFGAMRSHVQIVAAPTADT**

**NdiTrz1 (99) KFKIPDSVSKTVARELRRNKYLFAEYSLFSPHRAFQKWRNHGLLKHTKIPKDFFHGRIPIKLYPYQADGPRFILASPDPEQKEAPPPPAKPEYGAMRSHVQIVAAPTADT**

**SmaTrz1 (111) KYKIPDSVSKTVSKELYRYKYLYYEYSLYAPNRAFRTWKDRGILRHTKVPEDLFPDRIAIKLAPYQIGGARFILTPPP-QDKEAPPGPAKPEHGAMRSHVQIVAAPTADT**

**MgrTrz1 (6) RTVHWVTGALKSPPRVQRLSIGNKHLLQIRYYKVYRRQPPALDDEPEVILAPFKASYAFGDPTEAPSWLAIARVKSKGRSQHKRTQTDNSPYEASMYSKADIVWAPTADT**

**FgrTrz1 (1) -----------------------------------------------------------------------------------------------MSTTVEIASAPTVDS**

**FoxTrz1 (1) -----------------------------------------------------------------------------------------------MATSVEIASAPTVDT**

**FveTrz1 (1) -----------------------------------------------------------------------------------------------MTTTVEIASAPTVDT**

**NhaTrz1 (1) -----------------------------------------------------------------------------------------------MATSVEIAAVPTLDT**

**TatTrz1 (1) -----------------------------------------------------------------------------------------------MTTTVELASVPSADT**

**TviTrz1 (1) -----------------------------------------------------------------------------------------------MTTTVELAAVPSADT**

**TreTrz1 (1) -----------------------------------------------------------------------------------------------MTTTVELACVPSADT**

**AgoTrz1 (1) ------------------------------------------------------------------------------------------------MFRIIPVTQPTADT**

**CanTrz1 (1) ------------------------------------------------------------------------------------------------MFTFTPVAHPTADT**

**SceTrz1 (1) ------------------------------------------------------------------------------------------------MFTFIPITHPTSDT**

**ZroTrz1 (1) ------------------------------------------------------------------------------------------------MFYLTTVAHPTSDT**

**VpoTrz1 (1) ------------------------------------------------------------------------------------------------MFTFSPITHPTNDT**

**LthTrz1 (1) ------------------------------------------------------------------------------------------------MFTITHVTQPTVDT**

**KlaTrz1 (1) ------------------------------------------------------------------------------------------------MYQLIPITHPCLDT**

**PpaTrz1 (1) ------------------------------------------------------------------------------------------------MFEITTVAHPNDDL**

**CalTrz1 (1) ------------------------------------------------------------------------------------------------MFKVTTITHLTDDT**

**CduTrz1 (1) ------------------------------------------------------------------------------------------------MFRVTTITHLTDDT**

**CtrTrz1 (1) ------------------------------------------------------------------------------------------------MFKVTTITHLTDDS**

**CpaTrz1 (1) ------------------------------------------------------------------------------------------------MFKFTTIAHINNDT**

**CguTrz1 (1) ------------------------------------------------------------------------------------------------MFTFTTITHKTSDT**

**PguTrz1 (1) ------------------------------------------------------------------------------------------------MFTFTTITHKTSDT**

**DhaTrz1 (1) ------------------------------------------------------------------------------------------------MFTVTTISHKTSDS**

**PstTrz1 (1) ------------------------------------------------------------------------------------------------MFTISTVSHITADT**

**CluTrz1 (1) ------------------------------------------------------------------------------------------------MFSVSTICHKTSDC**

**YliTrz1 (1) --------------------------------------------------------------------MKAQRQAEQKQKKKAEKKAKGVLPDLSSTQSLYNFMSYSSDT**

**AmaTrz1 (1) -----------------------------------------------------------------------------------------------MEYSVQILGGGAHDM**

**BdeTrz1 (1) -----------------------------------------------------------------------------------------------MKYSLQISGTHSGDC**

**SpuTrz2 (1) -----------------------------------------------------------------------------------------------MRYYLQILSTNTVDS**

**SpuTrz1 (1) -----------------------------------------------------------------------------------------------MKSYIQILGTQTGDT**

**ScrTrz1 (1) -------------------------------------------------------------------------------------MSKNPNLRATKNFYLQFLTVNSRDT**

**SocTrz1 (1) -------------------------------------------------------------------------------------MSKDSSFRATKNFHLQFLTVNSRDT**

**SpoTrz1 (1) -------------------------------------------------------------------------------------MSKTVNFRATKNFYLQFVSVSSRDT**

**SjaTrz1 (1) -------------------------------------------------------------------------------------MSQKRVAHGNRTFTVQFVSTSSPET**

**ScrTrz2 (1) --------------------------------------------MKNLIYFKLFPLRGKNPITKLGHPLLNKLGTTVQLRRAGQQMVRRKEKRNTNRISLNVISSVAADN**

**SocTrz2 (1) --------------------------------------------MKNIIHNKLCTLYKKTPKIMLNHALPSKVGTFVQLRWVSQQIARRKENRNTNRISLNVISDVAADN**

**SpoTrz2 (1) ---------------------------------------------------MKASLLVPRRALLFGQLLPPKYSWYSVKRWQSQLTFRNKSKRNTNRIMLSVVSSLNPDS**

**SjaTrz2 (1) ---------------------------------------------------------------MFSVSRRQLLHSFKCLRLALRNYSQQQASLRNNDIHFSFITAAGVET**

**CneTrz1 (1) ------------------------------------------------------------------------------------------MISTETYQPNWAVRAVTTPT**

**TmeTrz1 (1) --------------------------------------------------------------------------------------------MEKDYRPNWVVRCLSVPG**

**AbiTrz1 (1) ------------------------------------------------------------------------------------------------MQSWVTTAVTSLSH**

**CciTrz1 (1) --------------------------------------------------------------------------------------MKKQAAASSTVMNWSASIQTTLSA**

**ScoTrz1 (1) -----------------------------------------------------------------------------------MSRGQKGPNRAADTMDWSASVLTTLSS**

**LbiTrz1 (1) -------------------------------------------------------------------------------------------------MNWSASVLTTVSS**

**PosTrz1 (1) -------------------------------------------------------------------------------------------------MDWSATVLSASSS**

**SlaTrz1 (1) -------------------------------------------------------------------------------------------------MSWSASVLSSTTS**

**HanTrz1 (1) -------------------------------------------------------------------------------------------------MNWSTSVVAAQSS**

**PplTrz1 (1) -------------------------------------------------------------------------------------------------MNWSVSVLSTATS**

**PhaTrz1 (1) -------------------------------------------------------------------------------------------------MNWSTSVVCGVSS**

**AbiTrz2 (1) --------------------------------------------MSGSNALKGYVILLILLLFFPALHYNSPQIFVVIVKIVHVAHRKPPTKIERPIRDWSATVATSSTT**

**MglTrz1 (1) ----------------------------------------------------------------------------------------------MAPMSLRVLAPPSSDT**

**MlaTrz1 (1) ---------------------------------------------------------------------------------MASGIGNPIRTFGIKTEINIRPITVCSTD**

**PgrTrz1 (1) --------------------------------------------------------------------------------MSQRGVSTMRPSIGANSPVLIKPVLVDASD**

**Ψ motif II**

**AbeTrz1 (16) PGTTLFLHY-DD-KRYFFGNLSEGTQRACIEN---NVRLSKLSEVFLTGKTT-----------------------------------------------WANNGGMLGML**

**TruTrz1 (16) PGTTLFLHY-DD-KRYFFGNLSEGTQRACIEN---NVRLSKLSEVFLTGKTT-----------------------------------------------WAGNGGMLGML**

**MgyTrz1 (16) PGTTLFLHY-DD-KRYFFGNLSEGTQRACIEN---NVRLSKLSEVFLTGKTT-----------------------------------------------WAGNGGMLGML**

**McaTrz1 (16) PGTTLFLHY-DD-KRYFFGNLSEGTQRACIEN---NVRLSKLSEVFLTGKTT-----------------------------------------------WSGNGGMLGML**

**CimTrz1 (16) PGTAILLHF-DD-KRYIFGNLAEGTQRACVER---GVKLSRVTDIFFTGKTS-----------------------------------------------WSTHGGIMGML**

**CpoTrz1 (16) PGTAILLHF-DD-KRYIFGNLAEGTQRACVER---GVKLSRVTDIFFTGKTS-----------------------------------------------WSTHGGIMGML**

**UreTrz1 (16) PGTALLLHF-DD-KRYMFGNLAEGTQRACVER---GFKLSRVTDIFFTGKTT-----------------------------------------------WSTHGGVMGML**

**AcaTrz1 (16) PGTTLIVHF-DD-KRYLFGHIPEGLQRACGHR---NIKFTHVTDIFMSGKTS-----------------------------------------------WANNGGLLGII**

**AdeTrz1 (16) PGTSLIIHF-DD-KRYLFGHIPEGLQRACNHR---NIRLSYVTDIFMSGRTS-----------------------------------------------WTNNGGLLGII**

**PbrTrz1 (16) PGTTILLHF-DD-KRYLFGHVPEGLQRACSHR---GVKLTHVTDIFMSGKTS-----------------------------------------------WTNNGGLLGVI**

**AclTrz1 (16) PGTAVILQF-PD-KRYLFGQIAEGTQRACTER---GIKLSYLTDIFLTGRTE-----------------------------------------------WANNGGLIGVI**

**AfuTrz1 (16) PGTTVVLHF-PE-KRYFFGQISEGTQRACTER---GVKLSYLTDIFLTGRTE-----------------------------------------------WANNGGLIGVI**

**NfiTrz1 (16) PGTTVVLHF-PE-KRYFFGQISEGTQRACTER---GVKLSYLTDIFLTGRTE-----------------------------------------------WANNGGLIGVI**

**AcrTrz1 (16) PGTTVLLHF-PD-KRYFFGHLSEGTQRACTER---GIRITYLTDIFLTGRID-----------------------------------------------WSNTGGLIGVI**

**AspTrz1 (16) PGTTVLLHF-PD-KRYFFGQLSEGTQRACTER---GIKITYLTDVFLTGQIQ-----------------------------------------------WGNTGGLIGVI**

**AflTrz1 (16) PGTTVQLQF-PE-KRYFFGQISEGTQRACTER---GVKLAYLTDVFLTGRME-----------------------------------------------WGNNGGLIGVI**

**AorTrz1 (16) PGTTVQLQF-PE-KRYFFGQISEGTQRACTER---GVKLAYLTDVFLTGRME-----------------------------------------------WGNNGGLIGVI**

**AteTrz1 (16) PGTSVLLYF-PD-KRYFFGQISEGTQRACTER---GVRLSYLTDVFLTGRME-----------------------------------------------WANTGGLIGVI**

**PchTrz1 (16) PGTSVCLNF-PD-KRYFFGQLSEGTQRACTER---GVKLALLTDVFVTGRSE-----------------------------------------------WANTGGLIGIV**

**AniTrz1 (16) PGSALLLHF-PG-KRYLFGQIAEGFQRACTER---GTKLTDVSDVFLSGRMG-----------------------------------------------WDTTGGLIGMI**

**PmaTrz1 (16) PGSTIILNY-PN-RKYSFGHLAEGSQRAFIEN---GFSFSYLNDLFVTGKTN-----------------------------------------------WANYGGTLGMI**

**TstTrz1 (16) PGSTIVLNY-PN-RRYVFGHLAEGTQRVFIEH---GFPFSYLNDMFVTGKTT-----------------------------------------------WHNYGGTLGMI**

**AbrTrz1 (16) PGTTLVLRT-PS-KHYVFGNLAEGTQRAMVQQ---GTRLLKAQDFFLTGRAE-----------------------------------------------WRNMGGLIGMM**

**CheTrz1 (16) PGTALVLRT-AT-KHYVFGSMAEGTQRAMVQQ---GTRLLKAQDFFLTGRAE-----------------------------------------------WKNMGGLIGMM**

**PtrTrz1 (16) AGTTLIFRT-SS-KHYVFGSMAEGTQRATVEQ---GVRLLKAQDFFLTGKAD-----------------------------------------------WKNMGGIMGMM**

**PnoTrz1 (16) PGTTLVLRT-AT-KHYVFGSQAEGTQRALVQQ---GARLLKAQDFFLTGKAE-----------------------------------------------WKNTGGFMGMM**

**MfiTrz1 (16) PGTTLLLHF-DN-KRYLIGSLSEGTQRACIQI---GARLLKVSECFLTGRTE-----------------------------------------------WKNTGGLIGMI**

**MycTrz1 (16) PGTTLVLHF-DS-KRYIIGSLAEGTQRACVQM---GARLLKVSECFVTGRTE-----------------------------------------------WQNTGGLIGML**

**SscTrz1 (16) PGTTLLLHF-DN-KRYLIGNIAEGTQRACVQC---KIGLMKVAEVFMTGKVD-----------------------------------------------WASTGGMLGMI**

**CglTrz1 (128) PGGCLMLHF-DN-RRYLFGRIAEGSQRTLVQR---KVSLAKIQDIFLTGCIN-----------------------------------------------WEATGGLLGMI**

**MthTrz1 (16) PGACLMLHF-DN-RRYLFGRMAEGTQRNMVQR---KVSLAKIHDIFLTGRVD-----------------------------------------------WETAGGLLGMI**

**TteTrz1 (16) PGACLMLHF-DN-RRYLFGRIAEGTQRTMVQR---KVSLAKIRDIFLSGAID-----------------------------------------------WQATGGLLGVF**

**PanTrz1 (16) PGACVLLHF-DR-RRYLFGRMAEGTQRAMVQR---KVAMAKIHNIFVTGTVD-----------------------------------------------WSTTGGLPGLM**

**NcrTrz1 (209) PGACLLVHY-DN-RRYLFGHLSEGTQRLFTEN---KIPAAKLSHIFLSGKTD-----------------------------------------------WATTGGLLGMI**

**NteTrz1 (209) PGACLLVHY-DN-RRYVFGHLSEGTQRLFTEN---KIPAAKLSHIFLSGKTD-----------------------------------------------WATTGGLLGMI**

**NdiTrz1 (209) PGACLLVHY-DN-RRYVFGHLAEGTQRLFTEN---KIPAAKLSHIFLSGKTD-----------------------------------------------WATTGGLLGMI**

**SmaTrz1 (220) PGACLLVHY-DN-RRYLFGHLSEGTQRLFTEH---KIPAAKLSHIFLSGKTD-----------------------------------------------WATTGGLLGMI**

**MgrTrz1 (116) AGSAIWLHC-DT-RRYLIGRIPEGTQRVLADRNN-DRSMIKLEEIMVTGTIG-----------------------------------------------SETCGGLMGMA**

**FgrTrz1 (16) PGTCLLVHT-DR-RSYIFGRPEEGTQRAFQSS---KIRMGPTEQVFLSGTVS-----------------------------------------------WQQVGGLFGYV**

**FoxTrz1 (16) PGTCLFVHS-ER-RAYLFGRPEEGTQRAFQSR---RLGMGATEQVFLSGSVS-----------------------------------------------WELVGGLFGYV**

**FveTrz1 (16) PGTCLFVHS-DK-RAYLFGRPEEGTQRAFQSR---RLGMGATEQVFLSGSVS-----------------------------------------------WELVGGLFGYV**

**NhaTrz1 (16) PGTCLFVHN-EK-RAYVFGRPSEGTQRAFNSR---RLGMGSTEHVFLSGSVS-----------------------------------------------WDQVGGLFGYI**

**TatTrz1 (16) PGTCIYLHH-DK-RSYVFGRVSEGTQRAFGSR---KIHMGGTEHVFLSGSVD-----------------------------------------------WDQLGGLCGYL**

**TviTrz1 (16) PGTCIYMHH-DK-RSYVFGRVSEGTQRAFGSR---KIHMGGTEHVFLSGSVN-----------------------------------------------WEQLGGLCGYL**

**TreTrz1 (16) PGTCIYMHH-DK-RSYVFGRVSEGTQRAFGSR---KVHLGGTEHVFLSGSVN-----------------------------------------------WEQLGGLCGYL**

**AgoTrz1 (15) KHPLLQLLAGHG-DRYFFGKIPEGAQRACIEG---KARLSRLNNIFLTGEMD-----------------------------------------------WASIGGLPGMI**

**CanTrz1 (15) QHPLLLLQADSG-ERYFFGKVSEGSQRCLTEN---KIKISKLGNIFLTGMLD-----------------------------------------------WSAIGGLPGMI**

**SceTrz1 (15) KHPLLLVQSAHG-EKYFFGKIGEGSQRSLTEN---KIRISKLKDIFLTGELN-----------------------------------------------WSDIGGLPGMI**

**ZroTrz1 (15) SHPLLLLQSHHG-DRYFFGKMAEGSQRCLTES---RTRIGKLQDIFLTGELN-----------------------------------------------WNSLGGLPGMI**

**VpoTrz1 (15) KHPLLLLTTENG-NKYFFGKIPEGSQRSITEN---KIRISKLENIFLTGELK-----------------------------------------------WNSFGGLPGMI**

**LthTrz1 (15) RHPLLLLQSEHG-DRFLFGQIPEGTQRTFPEN---KTRLSKLENIFLTGEMS-----------------------------------------------WNSIGGLPGMI**

**KlaTrz1 (15) KQPLLLLQSDHG-DKYFFGKIGEGSQRACNES---KVKFGKLNGIFLTGEMD-----------------------------------------------YSCIGGLPGLI**

**PpaTrz1 (15) NHPLIVVEEKINSKKFFIGSVTEGIQRTVNER---KIKLPKTESVFLTGKLS-----------------------------------------------WNRIGGLPGFL**

**CalTrz1 (15) TRPLICLTTRKG-FKYLFGKVPEGTQRVVNTFG-SEVKFSKLQGIFLTGSILS----------------------------------------------WSDIGGLPGFF**

**CduTrz1 (15) TRPLICLTTRKG-FKYLFGKVPEGTQRVANTFG-SEVKFSKLQGIFLTGSILS----------------------------------------------WSDIGGLPGFF**

**CtrTrz1 (15) NRPLVCLTTKLG-SKYLFGKVPEGSQRIINVIG-SEVRFPKLQGVFLTGSIFS----------------------------------------------WSDIGGLPGLF**

**CpaTrz1 (15) TRPLVALTTRQG-NRYLFGKIPEGAQRVINAVG-SEVRFPKLQSIFLTGTIFT----------------------------------------------WSDIGGLPGLF**

**CguTrz1 (15) SHPLVMLTNREG-FRYLFGKIPEGTQRVLNEE---RIRLGKLRGIFLTGILSS----------------------------------------------WSQIGGLPGLF**

**PguTrz1 (15) SHPLVMLTNREG-FRYLFGKIPEGTQRVLNEE---RIRLGKLRGIFLTGILSS----------------------------------------------WSQIGGLPGLF**

**DhaTrz1 (15) RHPLVMLTNREG-YRYLFGKIPEGSQRILNEN---RFRLGKLKSIFLSGTISS----------------------------------------------WSEIGGLPGLF**

**PstTrz1 (15) KHPLVMLTSREG-YKYLFGKVPEGTQRVLNEN---KFKLAKLKSLFLTGTLSS----------------------------------------------WSDIGGLPGLF**

**CluTrz1 (15) QHPMVMLTSREG-SRYFFGKVPEGAQRVLNEN---GVKLGKLKSIFLTGTVQT----------------------------------------------WSDIGGLPGLF**

**YliTrz1 (43) AGPGVTLQTMKG-EKFLFGHVTEGTQRAILEQ---KPRVNKMSGIYLTGPVT-----------------------------------------------WSTLSGLAGFL**

**AmaTrz1 (16) APSLLFKFDSQR---YLFN-CGEGTQRFCNEH---KFRLPKLKNIFLTKLD------------------------------------------------WTNLGGIPGML**

**BdeTrz1 (16) S-PSVIVRFDSG--RYLFN-CGEGTQRLCNEH---KIRLSKLRGIFMTRTK------------------------------------------------WDCIGGLPGMI**

**SpuTrz2 (16) --SPAVLLHFDS-QRYLFN-CGEGTQRFCHEH---KVRLAKVKNIFLTRVA------------------------------------------------WDCVGGLPGML**

**SpuTrz1 (16) --TPSVLVFFDS-QRYLFN-AGEGTQRFCIEN---KVRLGKLTNVFLTRIN------------------------------------------------WDTCGGIPGML**

**ScrTrz1 (26) SVIPCVHLFFDS-KRYIFGSIGEGCQRAILSQQL--KLS-KIKDVFLCHGGRMLLESPSSSLSSSSESISS------------ISSPLVHARNDTKVDWWDSCGGLIGFL**

**SocTrz1 (26) SVIPCVHLFFDS-KRYIFGSIGEGCQRAILSQQL--KLS-KIKDVFLCHGGRILSESPSS-VSPLSISSVS------------SPSPLTHSETNSQVDWWDSCGGLIGFL**

**SpoTrz1 (26) SCIPCIHLFFDS-KRYVFGSVGEGCQRAILSQQL--RLS-KIKDVFLMQGSSISSPDTYDSSSSSSTTSVSDMLQLDDRDKVIVSERNSMCSTVNYPTWWDSCGGFPGFL**

**SjaTrz1 (26) SPIPCIHLFFDS-KRYLFGSVGESCQRCVLSQQL--RLA-RIEDVFFCQG---------------------------------YSLQNYNQEDNNELSLWDCYGGLPGFL**

**ScrTrz2 (67) MIAPLLCVTLDD-RKYLIGNMGELMQLKFRNHPL--NYGGKLLRSFMMPG------------------------------------------SGRTYNPWSATAGLLGYV**

**SocTrz2 (67) LIAPLLCVTLDD-RKYLIGNMGELMQLKFRNHPL--NYGGKILRSFMMPG------------------------------------------SGRNYNPWSATAGLLGYV**

**SpoTrz2 (60) LIAPLLCVSLDN-RKYLIGSMGELTQMKFRSQAS--NYGGKSVSVFLMPP------------------------------------------SLQSLNAWGITAGLFGYL**

**SjaTrz2 (48) SAFPLIHLALPS-ERFLIGDFGEQTLRCVMLEKVRCSTKLKSAVVFPPVLNEHEFYQK-----------------------------------------WSSIAGLPGYS**

**CneTrz1 (21) KDTDLCLYVSFDNVRFLFG-CGEGTQRAFAQK---KIGFSRLGGVFIGSGELK------------------------------------------------GRGGLPGVL**

**TmeTrz1 (19) PDTSLSLYIAFDNARWLFG-CGEGTQRAFIQK--------GLVMRSLTGIIMPSGGS-------------------------------------------KDRNGLPGLI**

**AbiTrz1 (15) DTEPSILVNFEN-AKYMFN-AGENTNRAFLQS---SKNWKKMRSVFLTQVTS------------------------------------------------QRASGLTGII**

**CciTrz1 (25) DSEPSLMIAFDN-AKYMFN-AGENTVRSFAHSD---SNRKKLKALFLTQLGPH------------------------------------------------RSGGVAGLL**

**ScoTrz1 (28) DTEPSVLVTFPH-AKYIFN-VSENTNRAFLQNS---SNWKKIQGMFFTQASVQ------------------------------------------------RMGGASGLL**

**LbiTrz1 (14) DTEPTIIITFDS-AKYIFN-AGQNTNRAFLQSQ---RNWRKTRGIFFTQVGVE------------------------------------------------RAGGLAGLL**

**PosTrz1 (14) DTEPTVVVTFDS-AKYIFN-AGENTNRAFLQSR---RNWKRSRGIFFTSIGTE------------------------------------------------RASGLAGLL**

**SlaTrz1 (14) DTEPTVVVTFDS-AKYIFN-VGENTNRAFLQSR---RNWKKTRAMFLSSVGTQ------------------------------------------------RGSGLPGLL**

**HanTrz1 (14) DTEPTIFITFET-AKYSFN-MGENASRSLVQSR---RGWKKVRGLFLTQVGTQ------------------------------------------------RTSGLPGFI**

**PplTrz1 (14) DTEPTIVVNFES-GKYVFN-AGEGTGRSWLQSR---RHWRKARGVFLTSVGTQ------------------------------------------------RCSGLADMI**

**PhaTrz1 (14) DTEPTVVINFDS-GKYVFN-AGESTGRSFIQSRQ--HDWRKTRGVFLTSLGTQ------------------------------------------------RGAGLAGEM**

**AbiTrz2 (67) DTEPTILIRFND-SKYIFN-AGDNTTRAFIQHS---PTSTRMRAIFLTRVAS------------------------------------------------ATVGGLGGAI**

**MglTrz1 (17) SETPTLVLQCDS-RKYMFN-AGEGTTRISAQYR---ASNSRVEHIFLTRVAS------------------------------------------------ETMGGIPGLL**

**MlaTrz1 (30) TTSASLLLTFPQ-ARYLFN-CPENTSRSFAQAR---IPCKYLSAIFLATPRVE------------------------------------------------HSAGLHGFL**

**PgrTrz1 (31) SSSPSLLLSFDQ-SRYLFN-CPENTSRSFVQSR---IPQKNLNSIFLSSCRSS------------------------------------------------HCAGAYGML**

**AbeTrz1 (74) LTLSDTMASASGAMVEAESRKIKNLEERAKAAVRQKDSDELMARVKRRKLELEALPEFHGQR-AISLYGGPNLTHTLATGRTFVCRTGVPIEIREFGCLGC-EERDA---**

**TruTrz1 (74) LTLSDTMASASGAMVEAESRKIKNLEERAKAAVRQKNADELMARVKRRKLELEALPEFHGQR-AISLYGGPNLTHTLATGRTFVCRTGVPIEIREFGCLGC-EERDA---**

**MgyTrz1 (74) LTLSDTMASASGAMVEAESRKIKNLEERAKAAVRQRDADELMERVKRRKLELEALPEFHGQR-PISLYGGPNLTHTLATGRTFICRTGVPIEIREFGSHGC-EERDA---**

**McaTrz1 (74) LTLSDTMASASGAMVEAETRKIRNLEERAKSAVRQRDADELMARVKRRKLELEALPEFHGQR-AISLYGGPNLTHTLATGRTFVCRTGVPIEIREFGGVSN-EENDAS--**

**CimTrz1 (74) LTLADALSNASASLLESVDAKIAELKTMRDTTTDPSVRAKAESGLQKRLAERSKLEVQAQQRATLSLRGGPNLTHTIATGRRFICRQGMPICIQEFDGESG---IGNS--**

**CpoTrz1 (74) LTLADALSNASASLLESVDAKIAELKTMRDTTTDPSVRAKAESGLQKRLQERSKLEVQAQQRATLSLRGGPNLTHTIATGRRFICRQGMPICIQEFDGESG---IGNS--**

**UreTrz1 (74) LTLADALANTSSSLLESVDAKIAELETLRDTATDSRARTNAETNLLRRREERSRLEAIAEQRSTLSLHGGPNLTHTIATGRRFICRQGMPICIQEFNGDLDGDTSNAS--**

**AcaTrz1 (74) LTLAESANTSAWSQKEVYRTKAERLAKLAAETADPVQAESYRRRLEHFQERSNKLAVEFERRGKLAIHGGPNLTHTVATARKFIFRTGMPVYIHEFNGFSEGGGEGEGEG**

**AdeTrz1 (74) LTLAESVNTSAWSQREVRQAKTLRLKRLAEQTENREQWQYYRDRIELLEESNRRLAEGFDLRGKLAIHGGPNLTRTVATARKFIFRTGMPVYINEFNEFSNEFAVGKEEG**

**PbrTrz1 (74) LTVAESTSTSAWSQAEVHKIRVDRLEKLAEEAEDPVQAQWYRERLELNLKKNKRLAQDFE-KGKLAIHGGPNLTHAVATARKFIFRTGMPVFIHEFNEFMGLAENGE---**

**AclTrz1 (74) LTLADGLASTASALETAAREKEANRLKQ---GG----KP-APSGKQQQPSAQPQDGQAESERGSLTIHGGKNLTHTLATARRFVFRKGMPVFTKEYDSETLAKNATGA--**

**AfuTrz1 (74) LTLADGLASAANALELTAREKEAKGR----------------GGQPVKQQAQQQDGQAD--RGTLTIHGGRNLTHTLATARRFVFRKGMPVFTREYDSESLSKSGS-Q--**

**NfiTrz1 (74) LTLADGLASAANALELTAREKEAKGR----------------GGQHVKQQAQQQDGQAD--RGTLTIHGGRNLTHTLATARRFVFRKGMPVFTREYDSESLSKSGP-Q--**

**AcrTrz1 (74) LTLADGIASSNNALETAAREKEERFKGS-ESNA----NG---KKK-HGMSYAVHDGQLIPQRGTLTIHGGRNLTHSLATARRFVFRKGTPVFTREYDSEGMSKPGS----**

**AspTrz1 (74) LTLADGIASSNNALELLAREKEERSKESGQANA----TS---KKE-HGMSYAVHDGQLVPQRGTLTIHGGRNLTHTLATARRFVFRKGTPVFTREYDSEGMSKPDR----**

**AflTrz1 (74) LTLADGVASANTALEAMAREKEARQQKSGKSAK----QSPAKPKLEHGVPYAVKDGEAVAQRGTLTIHGGKNLAHTLATARRFVFRKGMPVFTREYDCEGMAKKGS----**

**AorTrz1 (74) LTLADGVASANTALEAMAREKEARQQKSGKSAK----QSPAKPKLEHGVPYAVKDGEAVAQRGTLTIHGGKNLAHTLATARRFVFRKGMPVFTREYDCEGMAKKGS----**

**AteTrz1 (74) LTQADTLASSTAAIETAAREKQARRQNNAKDGS----TQ--PQKLEHGVPYAVEDGQAVAQRGRLTIHGPRNLAHTLATARRFVFRKGMPVYTKEYDSETMAR-TM----**

**PchTrz1 (74) LTLADTVSSSLAAIEEDNQKKAARKAQREAEEGGKH-KPRAPQEQQHGRATVEREGEPALQKANLTIHGPANLTHTLATARRFVFRKGMPVYLKEYDSETLTKLIP----**

**AniTrz1 (74) LTKADARASSKEALEALEREKEANRQKR--------------GWTKKDTKQTPANAELEDPQHDLTVHGSKNLAHTLATARRFVFRQGLPVHTKEYDAESVAKRLR----**

**PmaTrz1 (74) LTLADSRTTGLGGLTEDDN---------------------------------KKTEKVEAALSRLTIHGGRNINHMLAAARRFIFRKSLPISVKEYDSKSLARDQQ----**

**TstTrz1 (74) LTLADSRTSGLKGLEEGD----------------------------------TKTSKVEAALSRLTIHGGRNVSHMLATARRFIFRKALPISVKEYDSTSLARDQRGN--**

**AbrTrz1 (74) LTLADSSTSSYTTAMDTFRQGQERGKK-----------------------------GEAPPKPHFDIYGPPNLKHTLATCRRFIFRKGIPINATEYVKQSPEK---DE--**

**CheTrz1 (74) LTLADASTSSYTTSMETFRLAQERGKK-----------------------------AEIPSKPHFDVYGPPNLKHTLGTCRRFIFRKGIPIVATEYTNQSPAR---DE--**

**PtrTrz1 (74) LTLADSSTSSYTTAMEIFREAQKRGKK-----------------------------GGGPQKPHFDIYGPPNLKHMLGTCRRFIFRKGLPINTTEYGNHVHEK---DE--**

**PnoTrz1 (74) LTLADASSSSYEQAMLMVQQARDKGRT-----------------------------ASEPAKHSFNIYGPPNLKHTMGTCRRFIFRKGLPIHVTEYADRPVAR---DE--**

**MfiTrz1 (74) LTLADSSAASQASSLEEVLKKAKMKAKRLGVADDP-------Q--K---MRELEDQAKKEMSNTLSFFGPPNLNHTLATARRYVFRKGMPVNVHEIRDSGLPERLPQE--**

**MycTrz1 (74) LTLADSSHASQAAAKEEAVKRIRSKAKREGYLEDE-------E--K---MRQLEEDAKRATDTTLNIFGPPNLNHTLATARRFVFRKGMPVNAHEIRDDAP---------**

**SscTrz1 (74) LTLADATSTSREAQFQHENAKMQKKG----------------------------ALAAKPEKAFLNIHGGENLTHLLATARRFVFRNGMPLYTNEYRPDRE------Q--**

**CglTrz1 (186) LTLADLKAASAADIDLTNEKLRSKGKKE--------------------------KEKDKSIPPHLNIHGGKNLVHLLASARRFILRKALPVHTRELQVDPRAELEG----**

**MthTrz1 (74) LTIADLKAASIADVEALNEKLRSQGK-----------------------------KDNKSVSAHLNIHGGKNLVHLLATARRFILRKALPVHPRELRHDPRADTAK----**

**TteTrz1 (74) LTLADLKAASAADLEQVNAKLRSRGK-----------------------------QDTKSVPAQLNIHGGKNLVHMLATARRFILRKALPVHPRELRHDPRSDES-----**

**PanTrz1 (74) LTLADVVAGAKAARADELAVREAKGLK----------------------------SKDRDIDDDLHIYGGRNLVHSLATTRRFIFRKGIPLSLNEIRTDSPSQRND----**

**NcrTrz1 (267) LTVADVIISAKTAVEEENKLRRQKNKP----------------------------EITHGGPQRIEIHGSKNLEHMLAAARRFVFRKGFPLDAHELRADPRAANPE----**

**NteTrz1 (267) LTVADVIISAKTAVEEENKLRRQKNKP----------------------------EITHGGPQRIEIHGSKNLEHMLAAARRFVFRKGFPLDAHELRADPRAANPE----**

**NdiTrz1 (267) LTVADVIISAKTAVEEENKLRRQKNKP----------------------------EITHGGPQRIEIHGSKNLEHMLAAARRFVFRKGFPLDAHELRADPRAANPE----**

**SmaTrz1 (278) LTVADVIISAKTAVEEENKLRRQKKKP----------------------------EIIHGGPQRIEIHGSKNLEHMLAAARRFVFRKGFPLDAHELRADPRAANPE----**

**MgrTrz1 (176) LTLAASKAMASKDSASRGKKTDTK----------------------------------PSKLTKLWLRGGRNLPYYLALGRSFILRQKMPLDVNEILADPRLDDPS----**

**FgrTrz1 (74) LTVGGVLDASKEQEAITNERRRERGLV----------------------------AFKESPPQALHIHGGENLNHTLAACRPVILRQPIVVKTHEHREDPRINSIK----**

**FoxTrz1 (74) LTVGGVLEASREQTAVMNEERKSKGQK----------------------------LTKQAAFETIHIHGGENLNHTLAACRPVILRQPISVRTHEHRENPRLEKIK----**

**FveTrz1 (74) LTVGGALEASREQTALINEERKSKGQK----------------------------LTKQAAFETIHIHGGENLNHTLAACRPVILRQPISVRTHEYRENLRLETIE----**

**NhaTrz1 (74) LTVGGTLEASREQTAILNEERQKKGKK----------------------------TVKPGVFEAIGIHGGENLCHSLAACRPVILRQPVSVVTYEHRTDRRAKTIE----**

**TatTrz1 (74) LSVGGAIESAKEYTTQENAKRGQKGQK----------------------------LLNKTLHAGISVHGADNLCHTMAALRPVIFRQPICVRTFEHRTDPRVADAA----**

**TviTrz1 (74) LSVGGAIESAKEFTTQENAKREQKGQK----------------------------LLNKTLHAGIAVHGADNLCHTLAACRPVIFRQPICVRTYEHRTDPRATDAA----**

**TreTrz1 (74) LSVGGSVESAKEYKMQENTKREQKGQK----------------------------LLNKTKHAGIAVHGAENLCHTLASCRPVIFRQPICVKTFEHRTDPRATDAA----**

**AgoTrz1 (74) LTASDQ-------------------------------------GN---------------KNMRLTY-GSALLGYVVSTWRYFVFRFGMDLDVNTMK-------------**

**CanTrz1 (74) LTIADQ-------------------------------------GK---------------DNLVLHH-GSQILNYIVSTWRYFVFRFGINLRTNTLVP------------**

**SceTrz1 (74) LTIADQ-------------------------------------GK---------------SNLVLHY-GNDILNYIVSTWRYFVFRFGIDLNDHIMK-------------**

**ZroTrz1 (74) LTVADQ-------------------------------------GK---------------ANLKLHH-GGDLINYVVSTWRYFVFRFGISLQTDAMK-------------**

**VpoTrz1 (74) LTLADQ-------------------------------------GR---------------DKLKLHY-NSPIINFIISTWRYYVFRFGIDLTTNVIQ-------------**

**LthTrz1 (74) LTLSDQ-------------------------------------GI---------------KCMNLIY-GNDIVNYIVSTWRYFVFRFGMRLNTKILK-------------**

**KlaTrz1 (74) LTASDQ-------------------------------------GK---------------KDISIFY-GSNILDYVVSTWRYFVFRFGLDLKVSTLK-------------**

**PpaTrz1 (75) LTVFDQK--S--------------------------------------------------RDSFFIVYGNPLLKYVIATWRYFIFRVGMNLDVKIVQDQ-----------**

**CalTrz1 (77) LTVSDA-------------------------------------TK---------------NGLTVMG-CERILSYILATWRQFVFRMGIKLHILDAE-------------**

**CduTrz1 (77) LTVSDA-------------------------------------TK---------------NGLTVMG-CERILSYILATWRQFVFRMGIKLHILDAQ-------------**

**CtrTrz1 (77) LTVSDS-------------------------------------TK---------------NGVNVIG-CGKLLSYIVATWRQFVFRMGITLNIIDTE-------------**

**CpaTrz1 (77) LTISDA-------------------------------------TK---------------KGIRVVG-ECNLLSYIVATWRQFVFRLGIDLEIINVD-------------**

**CguTrz1 (75) LTVSDA-------------------------------------TK---------------KDIDLYSNSSGLLTYLVSTWRYFVFRKGVQMNIKDTT-------------**

**PguTrz1 (75) LTVSDA-------------------------------------TK---------------KDIDLYSNSSGLLTYLVSTWRYFVFRKGVQMNIKDTT-------------**

**DhaTrz1 (75) LTISDS-------------------------------------TK---------------KSIDIFTNSGKITSFIVATWRYFVFRKGVELKINDSN-------------**

**PstTrz1 (75) LTLSDS-------------------------------------SK---------------KGIDVFTNSKSLLSYVVSTWRCFVFRKGAELKLFDTN-------------**

**CluTrz1 (75) LTISDA-------------------------------------TS---------------RGIDVFTNSSSVMAYVVATWRYFVFRKGIELNVLDTQ-------------**

**YliTrz1 (102) LTLVGMG-----------------------------------------------------KTDLDLRSCGHNVNWFCATWRHFVFHKTMAIRTDRMTKG-----------**

**AmaTrz1 (71) LTLNDVQELG--------------------------------------------------PKGPIHLHGPPNLTRFMASLRHFIYPRAMAFEVKELPGAQDATLAT----**

**BdeTrz1 (71) LTVADAG------------------------------------------------------LDSFRLYGPRNLSYFLAGTRKFMFRPNINISVVDIES-S--K-------**

**SpuTrz2 (71) LTLADAG------------------------------------------------------SSSMRLNGPNNLTHFMAATRHFIYRQSLDLSTFEFHETKG---------**

**SpuTrz1 (71) LSLADAG------------------------------------------------------NRHITVHGGKNLTHFMASTRHFVFKMSSMVQTHEFKDNAR---------**

**ScrTrz1 (120) LSMNDACDLP-------------------------------------------------HGESPFTLHGPSEIHYYLSCMRHFTYHTNVDLTIEGYTSSEAPEYADE---**

**SocTrz1 (119) LSMNDACDLP-------------------------------------------------QGENPFTLHGPSEIHYYMSCMRHFTYHTNVDLTIQGYSSSEAPEYVDE---**

**SpoTrz1 (132) LSLNDISEPGET-----------------------------------------------GEASPFVLHGPSEVHQFLSSMRHFTYHTNVNLTVQGYTSAEAPVFVDE---**

**SjaTrz1 (99) LSLADIGEPKGDG-------------------------------------------KDSIGNSKLVLHGSRELPKLLSCMRHFTYHDVLDIQLDIFDDECPATFSDD---**

**ScrTrz2 (132) QSSEIDTVLD-------------------------------------------------------LHA-PSRIASLIANSRNLTSGSSLKLNVIPFQNKLSEDHES----**

**SocTrz2 (132) QSSEIDTVLD-------------------------------------------------------MHA-PSGIASLIANSKNLTSGSSLKLNVIPFQNKISEDHES----**

**SpoTrz2 (125) QSSGIQNTWG-------------------------------------------------------LHA-PKPVISIIKKSHHLFSGSPLRLDLNSFSSEDNADATNS---**

**SjaTrz2 (116) LTFPLSQPWG----------------------------------------------------LHADAFTCKAFLRALSFSRTQSRAPDSHITPYENDS------------**

**CneTrz1 (79) MSASDAG------------------------------------------------------ISKIDVIGPPDISQYLATLRSSVIRDALTVNVTSYPRDVATG-------**

**TmeTrz1 (77) MTAADAG------------------------------------------------------IRNLDIIGPPDVGHHLASFRASLQRISINVKTHHPSTASSELR------**

**AbiTrz1 (72) MTFADN------------------------------------------------------AISKVDVVGPPGLRHYLASMRDYLFRDQLDVKTTEHIPDPGTPHP-----**

**CciTrz1 (82) MTLADAS-----------------------------------------------------AVNEMKIAGPTGLTHFLASMRFYTFRDNLAVSPLEIAPPPEHPKDP----**

**ScoTrz1 (85) MTFADA------------------------------------------------------TIQKLNVVGPPGTGHYLASARSYLYRNTMAVEVTDVPMTQARDTLP----**

**LbiTrz1 (71) MTFADA------------------------------------------------------TIKMLNVVGPPGLTHYLASMRLYTYRDSMRVKPIEASSSTTTASPP----**

**PosTrz1 (71) MTLADA------------------------------------------------------TIPKLDIVGPPGTTHFLAAMRSYTFRDAMPVNPIEASLSLSQEH------**

**SlaTrz1 (71) MTFADS------------------------------------------------------GLSSMDIVGPYGLLHFLAAMRKYTFRDNMSVNPREVPLTPSSSS-T----**

**HanTrz1 (71) MS------------------------------------------------------VADATARKLDVVGPSGILHLLASMRLYTFRNSLCVNVSEIQPTTGNTQLN----**

**PplTrz1 (71) QWSFPDSNCGSRV------LMF----------------------------------CADASIQAVKVMGPPGITHYLASMRGYLYRVTMSMKVMEVPSAPPVYAAGNL--**

**PhaTrz1 (72) RLASPSHYAQWSYP---GLLMF----------------------------------LADTSQPKMTIAGPPGMLHFVASMRAYLNRSVHNMYVNCIEAESFGRHGG----**

**AbiTrz2 (124) ASVAKT-------------------------------------------------------VHSLTVVGPPGIRHFIASLRGFIRG-ILRFSLFEYQSRGENRKPE----**

**MglTrz1 (74) MTLADG------------------------------------------------------GRTSVDVYAPPNLLYALATTRLYARRESMRVKPHEIPVTEP---------**

**MlaTrz1 (87) LGLADLQREK-----------------------------------------------------LVRIIGPLGTRYMMACGRLYTRREGMAVEAIEFVNRNRFEQ------**

**PgrTrz1 (88) MGLADGNKQ------------------------------------------------------SVRLIGPEGLRYMLACGRLFTRRQGMSVHVNEFKPTSTLQQ------**

**AbeTrz1 (179) -------------TIDISKPAWSDDHIKVWALPTYPSSSPRL---------------------------QPGKRTLDDFEGGG-----------------RAGAAATKAA**

**TruTrz1 (179) -------------TIDLSKPAWSDDHIKVWALPTYPSSSPRL---------------------------QPGKRTLDEFEGG-------------------EGAAATTAT**

**MgyTrz1 (179) -------------TIDLTKPAWSDDHIKVWALPVYPSTSPRL---------------------------QPGKRTLDDFEGG-------------------EGAAATKSA**

**McaTrz1 (180) -------------AVDLSKPVLSDDHIKVWALPIYPSTLPRP---------------------------QPGKRSLEEFEETG-------------------EAAATKAA**

**CimTrz1 (179) -------------QKVADKPTLSDDHIKVWALPILPSNNSGN---------------------------RSRKRSHDEFEEQVP----------------SSDSSKSSDQ**

**CpoTrz1 (179) -------------QKVADKPALSDDHIKVWALPILPSNNSGN---------------------------RTRKRSHDEFEEQVP----------------SSDSSKSSDQ**

**UreTrz1 (182) -------------PETLGKPTSSDDHIKVWALPIMPSTRPRI---------------------------NRRKRSHDEFEESVS----------------LSDPPSLSEQ**

**AcaTrz1 (184) VGGER---------RNVAEPTWTDSHLKVWALSISPTSSLGSPTGSV-GS-----------------MKSGRKRSLDEFQEGPNAGAAVAGQG------LESENASALDK**

**AdeTrz1 (184) QGGREGEGKGEGCERNVAKPTWTDSHLKVWALSVSPTSSRGSPVGSA-ED-----------------TKNARKRSLDEFQEGAGAAAANELRS------E-GKNVSDSDK**

**PbrTrz1 (180) ----------VETKKAVADPTWSDSHLKIWALSISPSSLPESPSSSA-GS-----------------TKSGRKRNLNEFQGG-----AAGAEE------T-SESEDGILS**

**AclTrz1 (174) ----------ADAADPFEQPTWADLNIKVWAMPIHPSPQSRVASPLPPR---------------------PRKRSHEEFQERDV-------------------DKPALDQ**

**AfuTrz1 (163) ----------ARTADPFEQPTWSDRNIKVWAMPIRPSPSQRSGRASPQVG-----------------PQSPRKRSLDEFQERHA-------------------DKPALSQ**

**NfiTrz1 (163) ----------ARTADPFEQPTWSDRNIKVWAMPIRPSPSQRSRRTSPQAG-----------------PQSPRKRSLDEFQERDA-------------------DKPALDQ**

**AcrTrz1 (171) ----------TETEDPCEQPTWSDSNIKVWAMPIKPSPGSQAPVISQ-VR-----------------SMSPRKRSLDEFEENNS-------------------ALDSIDA**

**AspTrz1 (172) ----------LGAEDPCEQPTWSDSNIKVWAMPIRSSPVPHLQATSQ-VR-----------------SMSPRKRSLDEFEETNN-------------------ASEPIDA**

**AflTrz1 (176) ----------AEAEDPFEQPTWSDDNIKVWAMPIRPLTSLQPKDVPR-VA-----------------PQSPRKRSLDEFREEVT-------------------TQEVIDP**

**AorTrz1 (176) ----------AEAEDPFEQPTWSDDNIKVWAMPIRPLTSLQPKDAPR-VA-----------------PQSPRKRSLDEFREEVT-------------------TQEAIDP**

**AteTrz1 (173) ----------PGTEDPFENPTWSDSNIKVWAMPIRPATSLPPKTASRQPS-----------------PQSPRKRSLDEYEERNV-------------------PLDPMDE**

**PchTrz1 (179) ----------VGAEDPFEKPSWSDSNIKVWAMPISPRPSARSQVTQP--R-----------------SQSPRKRSLDEFQESVQ-------------------TEDHVDQ**

**AniTrz1 (166) ----------TDQSDPFEVPTFSDENIKVWTMPISPS-----STTPR--------------------SQSPKKRSLDEFREDVR-------------------GLVEVDQ**

**PmaTrz1 (147) ------------EADPFTTPTFVDENIKVWPMALKPSYEMR-FESRS--G-----------------FKSPRKRSLNEFEERDNV------------------QSPIPDT**

**TstTrz1 (148) ----------SNDKDPFQKPTFVDENIRVWAMALKPSYEIRHFESRA--G-----------------FRSPRKRSLNEFEERDSE------------------QSTASDA**

**AbrTrz1 (150) --------------NGAIVPTWQDASIKVWALSVSPSDFEQDTQAEA-E-------------------LEKRRQHFDTQLNTFDAFQAPENER-------------PEDR**

**CheTrz1 (150) --------------KGVILPTWQDSNINLWAMSISPAGSQPDAQAEA-E-------------------IEERRRHFDTHLNTFEDFQAPENET-------------PEAR**

**PtrTrz1 (150) --------------NGAILPTWQDSMIKVWALSVSPTVSEHNAQAEA-E-------------------LESRRRHFDTKLNTFAGFQAPENES-------------PEAR**

**PnoTrz1 (150) --------------NGGIPPTWQDANIQVWALPVSPVRQQQDPEAEA-E-------------------LEALRQDFDARLNTFEDHKAPPNES-------------KEDR**

**MfiTrz1 (170) --------------NEEWKPFWADENIKVWPMSISPKATSSSQSPPA-S-------------------ISPRKRSFDEMHADGNGAMTAVAS--------------ELSQ**

**MycTrz1 (163) --------------ADDWAPMWADENIKVWAMSVSPDVGSS--KP------------------------NGKKRSIDEVDGE-REASVVPDD--------------ELSP**

**SscTrz1 (148) --------------RTNWSPTWSDDNIKVWAMAVKSEKSAK---R-------------------------SGKRSHDEFSDDSPDGSLAEKSQD----LILKEAQELQDE**

**CglTrz1 (266) --------------KDD--PDYEDENIRVWSIPLSKEKP-----TARPSR-----------------QSSPKKQKLSSSSNSHEN-----------LAAEEADQN-----**

**MthTrz1 (151) --------------KDE--PDYEDENIRVWSIPLSKEG------REEEEE-----------------EEKKKKPALSESSSSEPQEQEEG---P--TNTEEADQN-----**

**TteTrz1 (150) --------------KAE--PDYEDENIRVWSIPVSVTPL-----KPSASE-----------------PHSPKKRKLGPASDAEAEPEQD-------LAEEEADQR-----**

**PanTrz1 (152) --------------RAI--PDFEDDLVRIWHLPITSNS---------SAS-----------------RPGSRKRKHSDLEEGEEEAETAVTEQAELSDAEAQHIR-----**

**NcrTrz1 (345) --------------NSK--PDYEDENLKVWKIPLVATGMESR-SRSSSTG-----------------SQNPRKRKLNSSEEPEVMDTLVEGEDGEVVEDVPAG----VED**

**NteTrz1 (345) --------------NSK--PDYEDENLKVWKIPLVATGMESR-SRSSSTG-----------------SQNPRKRKLNSSEEPEVMDTLVEGEDGEVVEDLPAG----VED**

**NdiTrz1 (345) --------------NSA--PDYEDENLKVWKIPLLATGMESR-SRSSSTG-----------------SQNPRKRKLNSAEEPEAMETMVEGEDGEVVEDASAG----LEN**

**SmaTrz1 (356) --------------NSK--PDYEDENLKVWKIPLIATGMESR-SRSSSTG-----------------SQNPRKRKLNSSEEPEAMETVVEGEGEEAVDGVDDVPAGVEND**

**MgrTrz1 (248) ----------------NSEHDWEDENIRVWHVPLSANTSHSP---------------------------GGRKRAKVSHDDREPG------------------PG---ML**

**FgrTrz1 (152) --------------LLR--PDWEDDSIRVWKIPIQQGR---------PSS-----------------SKKRRRSSAGSGG--SPDSSE---------PQFKEPAT-----**

**FoxTrz1 (152) --------------SIE--PDWKDDVLRVWKIPIQRDR---------PSS-----------------PKKRRRS---SAI--VGDQKK---------PQFKEWSP-----**

**FveTrz1 (152) --------------SIE--PDWKDDVLRVWKIPIQRDR---------SSS-----------------PKKRRRSSA-IAI--SEDQEE---------AQFKEWSP-----**

**NhaTrz1 (152) --------------DLE--PDWQDDAIRVWNIPVQRER---------SSS-----------------PQKRRRS---SSI--VGDLSPSS-------PQFKGLAK-----**

**TatTrz1 (152) --------------SLE--PDWSDDAIKVWKIPVRRAR---------SSS-----------------PRKRRRSSQTRDRNSPKDASSEE-------DSFQPRAS-----**

**TviTrz1 (152) --------------NLE--PDWSDDAIKVWKIPVRRAR---------SSS-----------------PRKRRHSDPAMD--VAEDASSEE-------ATFQPRSS-----**

**TreTrz1 (152) --------------NLE--PDWSDDAIKVWKIPVRRAR---------SSS-----------------PRKRRHSDLAQDGVVDAMSESASSE---EANNFQPRAA-----**

**AgoTrz1 (118) -----------------DGETYRDELISVKALNVRR-----------------------------------------PGMMGIEP------------------------L**

**CanTrz1 (119) -----------------SKNIYKDKLLQVNPIVISK-----------------------------------------KHSEEVKL------------------------G**

**SceTrz1 (118) -----------------DKEVYKDKIIAVKSFNVLK----------------------------------------NGGEDRLGV------------------------F**

**ZroTrz1 (118) -----------------DQEIYSDGLLRVKSIIVPQ----------------------------------------SGGNDNL-V------------------------F**

**VpoTrz1 (118) -----------------DQELYQDKSIIVKSITVTKE-----------------------------------NE--TEGKNSDNI------------------------F**

**LthTrz1 (118) -----------------DGDVFENKLMKVRSIVVNS----------------------------------------PSQFSSMKS------------------------L**

**KlaTrz1 (118) -----------------NSECYQDSTITVKSIVISN------------------------------------SA--NVDQCVQQH------------------------Q**

**PpaTrz1 (122) ------------------EEELITDSFKANVINISP--------------------------------------------------------------AKP------KAT**

**CalTrz1 (121) -----------------TNPTIVDEEVVISPIMIDPA-----------------------------------NQ--TSPPE----------------------------N**

**CduTrz1 (121) -----------------ANPTLEDEEVVISPITINPA-----------------------------------HQ--ISPPE----------------------------N**

**CtrTrz1 (121) -----------------TNPEITGEEIVISPIRIDPV-----------------------------------EH--EPHPE----------------------------N**

**CpaTrz1 (121) -----------------QNPVISNEEIVVKSIKIAPK-----------------------------------NS--AKSDVPVVT------------------------K**

**CguTrz1 (120) -----------------TAEFIADSTVLVKPIRTASS-----------------------------------RQ--SEVPA----------------------------T**

**PguTrz1 (120) -----------------TAEFIADSTVLVKPIRTASS-----------------------------------RQ--SEVPA----------------------------T**

**DhaTrz1 (120) -----------------EHDIIADSNLIVKPIKIESNK--------------------------------TTSI--NED------------------------------V**

**PstTrz1 (120) -----------------EENIIGDNNLVIRPIKIASN---------------------------------VENI--NQDSA----------------------------V**

**CluTrz1 (120) -----------------SENLIGDSTTVFRPVKIPSR------------------------------------S--SSPMG----------------------------A**

**YliTrz1 (148) ---------------------HVTDEINIGGVLITPTESRLQ--------------------------------QE-EQ------------------------------H**

**AmaTrz1 (127) --------------------CLQDENLRIVPVLIFPTSAAHDAASAAAADG------------------STGTSSAPNGDSVSSS------------------SSSSTTP**

**BdeTrz1 (117) -------------------FDYSDENIHIKAIALTPELGDSSICPISPLAG--------------------RKRSSHESNR----------------------------E**

**SpuTrz2 (118) -------------------EPFQDENITVHPVYLAPEPIVSP----------------------------ETQNAATNGPDVITFS-----------------RKRKNES**

**SpuTrz1 (118) --------------------PFQDENLSVLPVPIHPIDPSLASNDTTTTAVKKVAIDSGNGKMLPVAISIPKNAPRASFNRPSSSR-----------------NGQQTPS**

**ScrTrz1 (178) ---------------------NIRV----TPVVISLPSGGRKRK---------------------------------YSDS--RTS------------------------**

**SocTrz1 (177) ---------------------NIRV----TPVVISLPSSGRKRK---------------------------------YSDS--PAL------------------------**

**SpoTrz1 (192) ---------------------NICV----TPVVVSLVKNSFKKRKH-------------------------------ENIN--RGT------------------------**

**SjaTrz1 (163) ---------------------NINV----YPVVIQNPTLQVSPAK-----------------------------------------------------------------**

**ScrTrz2 (182) -----------------------------------FEFREKDFC------------------------------------------------------------------**

**SocTrz2 (182) -----------------------------------FEFREKNFC------------------------------------------------------------------**

**SpoTrz2 (176) -----------------------------------FYLDEPEFC------------------------------------------------------------------**

**SjaTrz2 (162) --------------------------------------------------------------------------------------------------------------**

**CneTrz1 (128) ----------------TSVKLFESRNITVHGVALVPKLN-------------------------------------------------------SSS-----------IN**

**TmeTrz1 (127) -------------------QIYDHPSINIKAFSILPS-----------------------------------------------------------S-------------**

**AbiTrz1 (123) ------------------EIVYKDENITVYSFSILRHALSSLGELTEELEEVG------------------QKRRRSILPESPSK------------------VPRFGLK**

**CciTrz1 (135) ------------------EPVFQDENITVYALPLTPDDVSSPSAQSEDSSLK--RKRET-------SPDQPRKRSTTSVEDEASHLS---------------AEQRSLTE**

**ScoTrz1 (137) ------------------EPVLKDRDISIYGLPVLPDATCPSERSPSPTSLK--RKREP-------SPERSSKRTYAPAER--------------------MDADVTLDS**

**LbiTrz1 (123) ------------------RPVYKDENVAIYALTISSSPDSEMAAIATDSA---TTSDLS-------APDIPSKRKREATPDSPRKR----------------SSNQTLKQ**

**PosTrz1 (121) --------------------LYKDENITVYSIPIMPTLSALDGALTMSKR---KRDASP-------SSPVKRARVEEGTPP---------------------KSYETLSE**

**SlaTrz1 (122) ------------------DPVYKDDNITVYALPILPYIETESSISSQDSFGSHTVIPTS-------SSARNSKRKRTPSPPSSAKRSS-QITA--------SESELSLQE**

**HanTrz1 (123) -----------------PTPAFSDENITVYSIPLIPGSTSTSDLASVAAD----TAAPE-------AS-SLLKRKRTPSPDAPSKRSPTSRSD--------QDSKTVLER**

**PplTrz1 (139) ---------------DEPQPAYTDENIKLYAIPLHAVPASPAEGENTAAV----HGKRK-------RSPSPDSSFKRPAPSDPAAPTTSDEDL--------EHVPRPLLE**

**PhaTrz1 (141) ---------------LQPDPVFQDENLKIYGIPLTVTPANSAS-------------DLE-------AG---SKRKRSPSPDQPSKRGKQTEGT--------SPQTPTLWE**

**AbiTrz2 (174) -------------------AIYTDENITVFPIPITPTIVADE----------------------------PSERRHSNLDAN----------------------------**

**MglTrz1 (121) ------------------HVCFADEHIELQAIPLLPAQHR-----------------------------------------------------------------ELYAA**

**MlaTrz1 (138) --------------------VFKDDLITVYALGSEAIASKRALECSNNSS------PAN-------KRQKVAGSVNVDPESICSET------------------------**

**PgrTrz1 (138) --------------------VFQDHLIRVYALGNDRKDQTQVLMNRKRS-------PDS-------TAISDLSHKRTKLEPGNSE-------------------------**

**AbeTrz1 (232) REKDLVTRQKIVLDMFNSQWR-LDTLVETPLAEVSLPATLFIRDKDTGEVKPYEGPKP--GDGEALP---------------------------------------NINV**

**TruTrz1 (230) REKDLVTRQKIVLDMFNSQWR-LDTLVETPLAEVSLPATLFIRDKDTGEVKTYEGPKP--GDGEVLP---------------------------------------DINV**

**MgyTrz1 (230) REKDLVTRQKIVLDMFNSQWR-LDTLVETPLAEVSLPATLFIRDKDTGEVKPYEGPKP--GDGEALP---------------------------------------NINV**

**McaTrz1 (231) RDQDLVTRQKIVLDMFNSQWR-LDTLVETPLAEVSLPATLFIRDANSGEVKPYEGPKP--GDGEPLP---------------------------------------DINV**

**CimTrz1 (233) RFKDQVTRQAVVSEMFNSDWR-MDTLVEMPIAEVSLPATLFVRNPETQEIQPYTGPKP--GDSEPLP---------------------------------------DIMV**

**CpoTrz1 (233) RFKDQVTRQAVVSEMFNSDWR-MDTLVEMPIAEVSLPATLFVRNPETQEIQPYTGPKP--GDSEPLP---------------------------------------DIMV**

**UreTrz1 (236) RQKDQIFRQAVVSQMFNSDWR-MDTLVEMPLAEVSLPATLFIRNPDTQQIQPYTGPKP--GDSEPLP---------------------------------------DIRV**

**AcaTrz1 (261) RAQDQIARQAVVSDMFNSDWR-LDSLVEMPLSEVRHPAKIFVRNRETHKLEPYTGPGP--DATDTVP---------------------------------------DITV**

**AdeTrz1 (269) RAQDQIVCQAVVSDMFNSDWR-LDSLVEMRLPEVRLPAKVFVRNRETHKLEPYTGPVP--DGTQPVP---------------------------------------DIPV**

**PbrTrz1 (250) RAQDQITRQAVVSDMFNSDWR-LDSLVEMPLSEVRMPARIFVRNSENHKIESYTGPKP--GGTEELP---------------------------------------DLNV**

**AclTrz1 (234) HTQDQIMRQSVISDMFNSSWR-MDALVETPLVEVKMPAVMFVRNPETKDLEPYKGPAP--GSNEPLP---------------------------------------DIKV**

**AfuTrz1 (227) RTRDQIMRQSVISDMFNSSWR-MDALVETPLAEVKMPAVMFVRDPETKDLKPYHGPAP--GSDKPLP---------------------------------------DIKV**

**NfiTrz1 (227) RARDQIMRQSVISDMFNSSWR-MDALVETPLAEVKMPAVMFVRDPETKDLKPYHGPAP--GSDKPLP---------------------------------------NIKV**

**AcrTrz1 (234) RTRDQVIRQSVITDMFNSTWR-LDALHETKLADVKMPAVMFVRNPVTKDLEQYKGPAP--GSNEPLP---------------------------------------DINV**

**AspTrz1 (235) RTRDQVIRQSVITDMFNSNWR-LDALHETKLADVKMPAAMFVRNPVTKDLEQYKGPAP--GSNEPLP---------------------------------------DITV**

**AflTrz1 (239) RTRDQIIRQSVITDMFNSTWK-LDALVETPLAEVKMPAVMFVRNPETRALEQYTGPAP--GSNEPLP---------------------------------------DIKV**

**AorTrz1 (239) RTRDQIIRQSVITDMFNSTWK-LDALVETPLAEVKMPAVMFVRNPETRALEQYTGPAP--GSNEPLP---------------------------------------DIKV**

**AteTrz1 (237) HTKNQIMRQTVITDMFNSTWR-MDALVETPLAEVKMPAVMFVRNPETKDLDRYTGPLP--GSGEPLP---------------------------------------DIKV**

**PchTrz1 (241) RTKNLLVTQSIVQDMFNSTWK-MDALHETPLAEVMMPAAIFIRNPETKDLEQYKGPVP--GGPDALP---------------------------------------DIKV**

**AniTrz1 (222) QSKDQLVRQSVVSHMFDSTWT-LDALEETRLADVKMPAQIFVRNPETKDLDKYTGPLP--GGDEEVP---------------------------------------EMTV**

**PmaTrz1 (207) FAKDQMLREHIVKDMFDSDWK-LDALYEENLYDVKQPAVMFVRNPKTKDLERYQGPLP--GSSDSVP---------------------------------------NIKV**

**TstTrz1 (211) RDKDQLLREHIVKDMFDSDWK-LDALYEENLYDVKLPATMFVRNPETKDLERYQGPLP--GSSGIVP---------------------------------------NIKV**

**AbrTrz1 (213) EVRYDRIRTATIKHMFDSNWR-MDTLVERNISEVEMPAAMFVRNSDTHGYESYQGPKP--GGSDPLP---------------------------------------DITV**

**CheTrz1 (213) EARYDKIRTATIKFMFDSNWS-FDTLVEQHISEVKMPAAMFVRNPETHSYEPYDGPKL--GGSEPLP---------------------------------------DITV**

**PtrTrz1 (213) EKRYDQIRTATIKFMFDSNWS-FDTLVERHISEVDMPAAMFIRDPATKGFQPYHGPKP--GGSDPLP---------------------------------------DITV**

**PnoTrz1 (213) DARYDRIRSATLKFMFDSNWS-FDTLVERHIAEVQMPAAIFVRNPDTHGYEPYQGPRP--GGSEPLP---------------------------------------DITV**

**MfiTrz1 (232) EDRDYLTVKAIVGEMFNSSWR-LDTLYETALSDVKLPATIFVRNAETNKIEKYTGPLP--GDAKNPPS------------------------------------DPQMKV**

**MycTrz1 (218) KDRDYLSAKAVVSEMFDSSWR-LDTLHETPLAAVRLPAELFVRNPQTNKISKYIGPLP--GAVGEQP-------------------------------------DPDQIV**

**SscTrz1 (212) EDNKHQIRKSVVSSMFDSDWK-LDTLHSMKLKDVRMPAAIFIRDS-DGKIQRYQGPKP--GGAQEVP---------------------------------------DIEV**

**CglTrz1 (322) ----IR--EAVVNDMFCSKWK-LDTLREMRLADVQLPAKIFVRNA-QGHIEAYAGPP-----ASEAP---------------------------------------NTKV**

**MthTrz1 (212) ----IR--EAVVKDMFASKWK-LDTLRELRLADVQLPAKIFIRNE-QGHIEPYEGPP-----PSEAP---------------------------------------DTKV**

**TteTrz1 (210) ----IR--EAVVKDMFCSKWN-LDTLREMKLKDVQLPAKIFIRND-QGHIEPYDGPDP---SSSSCP---------------------------------------DINV**

**PanTrz1 (215) -----Q---AAVAGMFSSTWS-LDTLHEVNLRDADPLAKIFFRSE-GGMIEEYKGPRP--GNTEKLP---------------------------------------DIRV**

**NcrTrz1 (417) DEKTHLLMQTVIKHMFDSDWK-ADTLVEQPLHKVKLPAKIFVRDD-EGKITVYKGPLP--GRDANVP---------------------------------------DIRV**

**NteTrz1 (417) DEKTHLLMQTIIKHMFDSDWK-ADTLVEQPLHKVKLPAKIFVRDD-EGKITVYKGPLP--GRDANVP---------------------------------------DIRV**

**NdiTrz1 (417) DEQTQLLMQTVIKHMFDSDWK-ADTLVEQPLHKVKLPAKIFVRDD-EGKITVYKGPLP--GRDANVP---------------------------------------DIRV**

**SmaTrz1 (432) EEKTHLLMQTIIKHMFDSDWK-PDALVEQSLHKVKLPAKIFVRDD-EGKITVYKGPLP--GRDADVP---------------------------------------DIKV**

**MgrTrz1 (294) SETDQELVKSVLMTMFETDWR-LDGLVETNLSQASRDATLYVRK--NGSLEEYRGPMP--GGSEPVP---------------------------------------DITV**

**FgrTrz1 (204) LSDYRLAASLVENLMFNGTVRGNGTVIPIKLANARPNDI-LFWRS-NGDFQPYNGPRP-GHANFEDK---------------------------------------DEFV**

**FoxTrz1 (201) LSDPEYASTLVEKVMFNGKLKGNGMLIPVKLSEVKPRDT-VFTRQ-EGEIKLYKGPRPGDGSELTEP---------------------------------------DQTV**

**FveTrz1 (203) LSDPEYATTLVEKVMFNGRLKGNGMLIPVKLSEVKPKDTTIFIRE-EGKAKLYKGPRPGDGSKLTEP---------------------------------------DQIV**

**NhaTrz1 (203) LSDPEYAATLVEKTMFNGHLKGNGLLVPTQLSQVKPTDT-VFVRK-GSDVSLYKGPRPGDGAKISNP---------------------------------------NETV**

**TatTrz1 (208) ISDPEVASAIVERIMFNGSLKNRSVLVPKKIRDLKPTDKAAIIE--NSSIRLYKGPYAADGVTLPNP---------------------------------------DDTV**

**TviTrz1 (206) VSDPEIASAIVERIMFNGSLKNRSVLIPKKIRDLKPTDKAAIVE--NGTIRLYKGPYMTDGAEVSNP---------------------------------------DDTV**

**TreTrz1 (212) VSDPDVASAIVERIMFNGSLKNRSVLIPKKIRDLKPTDIAAVVR--GGTMKLYKGPYMTAGVEVPNP---------------------------------------DDTV**

**AgoTrz1 (146) GKAAVQQFKFLAERMFPMLAP-----------------------------TAYQDPS-------SDPEL-----------------------------------SMRLPK**

**CanTrz1 (147) NKTVESLLNDIVAHMFPKKTD----------------------------TNMKYEPS-------KDPHL-----------------------------------NVKLPE**

**SceTrz1 (147) DSFQKGVLRSIVAKMFPKHAP-----------------------------TDRYDPS-------SDPHL-----------------------------------NVELPD**

**ZroTrz1 (146) NERQNMALKSIVANMFPKNGP-----------------------------TSRYDPS-------SDPFL-----------------------------------NVELPT**

**VpoTrz1 (150) TKELKSVINSIASSMFPKKSP-----------------------------ISKFDPS-------TDPHL-----------------------------------NVSLPN**

**LthTrz1 (147) TSQLNTGLKYIVSKMFPEHEP-----------------------------TARHDPA-------SDPQV-----------------------------------NVNLPR**

**KlaTrz1 (149) SLGFTNGLKNIIARMFPEVNP-----------------------------TDEQDPS-------ANSTL-----------------------------------NVDIPP**

**PpaTrz1 (146) NNVSTSLLDAIISHIFPLNIN----------------------------LNNYSENRK----------------------------------------------NFDRIS**

**CalTrz1 (149) SSKLVRQIKKLASLMFPLDTS----------------------------QVNSRDPES----YKSDPT------------------------------------QNDIHT**

**CduTrz1 (149) SSKLLRQIKKLASLMFPLDTS----------------------------QVNSRDPES----YKSDPT------------------------------------QNDIHT**

**CtrTrz1 (149) SNVLVRQVKKLASLMFPLDTS----------------------------EVNSRDPES----YRSDPT------------------------------------SKDIHT**

**CpaTrz1 (153) ADKVLTQIRKLASLMFPLDTG----------------------------EVNSRNPES----YKSDPS------------------------------------SKDIHT**

**CguTrz1 (148) PSGIVPSLKKLMSLMFPSDTS----------------------------AVNDPDPAS----YKSDPS------------------------------------ETEIHT**

**PguTrz1 (148) PSGIVPSLKKLMSLMFPSDTS----------------------------AVNDPDPAS----YKSDPS------------------------------------ETEIHT**

**DhaTrz1 (149) SNRLHQQLKKLISLMFPMDTT----------------------------KVNDPDPTS----YKSDPS------------------------------------ETEIQT**

**PstTrz1 (150) AKRLAVQMKKLVSLMFPKDTS----------------------------KVNDRDPNS----YKSDPT------------------------------------ETDIHT**

**CluTrz1 (147) SAKILSQLKKLTSLMFPPDS-----------------------------AANSRDPAS----HKSDPS------------------------------------ENEIQT**

**YliTrz1 (174) QNGEKLAPRNLQPLRRDTPWTNVNRVLKTMFSS--------------------RG---------------------------------------------------GDYG**

**AmaTrz1 (181) SNAPPPRTGKPIPLPMGASIPAALVPRSARATATKVPAARLP--------KRRRSPSPTTTSAHLPPQVR------------------------------------QCVE**

**BdeTrz1 (160) SKDDAYFLQKIIKQMFPKQPE-----------------------G-LCRITMQKQVQDIWDDFKSRG-------------------------------------------**

**SpuTrz2 (164) QNDHLICKRAFLRRMFPVDQGYHPLSDHTGTEITDVTIPAG------EGETATVVAMEDIKDSN-----------------------------------------EDEVV**

**SpuTrz1 (191) SNRSAATVHAGQKRKADDEDPDSTSVQDESMAYKREIIRQMFNSGESLSIKVGKGDAFHERKDSSRFDR--------------------------------QSGQSHNLE**

**ScrTrz1 (204) ----SNTDITSTTSDSTPHWFSHSSNETAFVVDN---------------------AL-----------------------------------------------------**

**SocTrz1 (203) ----SSNENTSATSDSTPHWFTHSSNETAFVVDN---------------------AL-----------------------------------------------------**

**SpoTrz1 (220) ----NARPLKEDRANTSPHWYSHVSNDTSFVVEN---------------------AM-----------------------------------------------------**

**SjaTrz1 (183) -----RRKREHAPKDDENSLVNEPNN----VVHL---------------------GS-----------------------------------------------------**

**ScrTrz2 (191) --------T--IQGEMSPSWSYLSFM------------------------------------------------------------------------------------**

**SocTrz2 (191) --------T--IKGEIFPLWSYLSFM------------------------------------------------------------------------------------**

**SpoTrz2 (185) --------T--IKGNIYSNWSFLSFN------------------------------------------------------------------------------------**

**SjaTrz2 (162) -------SMVIYEDPFISIKQ-----------------------------------------------------------------------------------------**

**CneTrz1 (156) EVNMVYSPYDPYSATFQPSHLSPSDLQKWCDHVVRDMFQNNAQARLSKRAPSPTPPPTSPRLGSPIGS--------------------------------------PKRP**

**TmeTrz1 (146) -QSDSNDQSSPIPTPPPFIPITSTVTNPVPHLHPPTQPWALPPDQKNTWIEHIMDDMFSRHFAKPSP---------------------------------------DTPG**

**AbiTrz1 (179) PPKQGLEPDNCIKFMFKSDES-------------------------SQRWVKKEGESR-IADDYGRP---------------------------------------NIPR**

**CciTrz1 (203) LMADSSFKPQSLTGENAAEYRRLLVQTMFPADVAKVQQSEKKGQGEAMKVQGHGKTKKQRKAEEAAATAASAISAKDAIKRRSPSPAPFPVDKDAQKFDDYRRTPRHLPK**

**ScoTrz1 (200) ILAREDFTPAMLSGAHATEYIQRVHGCMFSKQTQ----------------GGGKKSRKQQNQQVKGS--------------------------GPIRAQPDVFRRGSLPP**

**LbiTrz1 (189) IMEDPDFLPEDLTDERAHEWREHVIKTMFPDTKI-----------DSPKKGG-KAKVSKAVQQDSGN--------------------------GSANVDDCRRARAAPPK**

**PosTrz1 (180) ALNDPKLCPVRLEGPVAHEYRKLMVKTMFPASQK-----------SSISSPSQKSGKRKGKATDIAV--------------------------DTLPEVDIYRRPRIP-N**

**SlaTrz1 (198) AMKKSDFLPAVLRGDLAQSWRRFVIQTMFPGTAIPKVIESS----VKPQVISQKGAKKKGKIDDDAS--------------------------VAPTPPVAKISRISQPP**

**HanTrz1 (196) LRDDANFSPSSLVGAEAQEWRRLVVDHMFTWTEPP----------PQRTLNARASKR--VRVKTEGTI---------------------KASSTARR---GKGIFNPAGS**

**PplTrz1 (215) RIAEPGFSPLELSGEDAEEWRRMVVANMFPCSPPPP---------AEEPIKKRKQKQ--KDVTPPVVA---------------------EAELKPVQPQVDPIMYNAAIA**

**PhaTrz1 (205) RSREAGFSPSTLTGDDAQEWHRMILKTMFNPKEPDA---------KDLKAKKDANAKGSFGVKPPGSE---------------------VSVSAPVT---EGDVRKPAWE**

**AbiTrz2 (209) -KSPRIMKAKTAVGLTADEVRQAAVYHMFKRNS------------------------------------------------------------------------ANIRP**

**MglTrz1 (148) QSSDLPSFDPVLQPWNQPHWRPSSLRGADALQWFRCIVQDAWKAEEASTILPDTVSSG----------------------------------------------NAHGNI**

**MlaTrz1 (191) --PDAPVEGTGCTTRDPSAWLDSVLQDMFRPTITGS-------------------RK-----------------------------------------PKPDTKTGPTPA**

**PgrTrz1 (189) --PPTGVQP--VLN--SHDALPEVIDDMFRASGTS----------------------------------------------------------------RPLGKKNTTPA**

**flexible arm**

**GP motif Walker A-like motif**

**AbeTrz1 (300) LVRQPWP------------GAMVESLPATTPSDCAVSYIVRGHDSRGKFDRKRAESLNIADKSDFRRLASGE--TVISADGK----------------------------**

**TruTrz1 (298) LVRQPWP------------GAMVDSLPATTPSDCAVSYIIRGHDSRGKFDRKRAESLNIADKSDFRRLASGE--TVVSADGK----------------------------**

**MgyTrz1 (298) LVRQPWP------------GAMVDSLPATTPSDCAVSYIIRGHDSRGKFDRKRAESLNIADKSDFRRLATGE--TVISVDGK----------------------------**

**McaTrz1 (299) LVRQPWP------------GAMVDSLPATSPSDCAVSYIVRGHDARGKFDRKRAESLNIADKSDFKKLAMGE--TVIAADGK----------------------------**

**CimTrz1 (301) LVRKPWP------------GALVESLPPTSPSECAMSYIIRNHDVRGKFDAKKAAALGVQRGPDYRKLTENQ--SVLSKDGQ----------------------------**

**CpoTrz1 (301) LVRKPWP------------GALVESLPPTSPSECAMSYIIRNHDVRGKFDAKKAAALGVQRGPDYRKLTENQ--SVLSKNGQ----------------------------**

**UreTrz1 (304) LVRKPWP------------GALVESLPATSPSECSMSYIIRNHDVRGKFDAKKAAALGVQRGPDYRKLTENQ--SVLSKDGQ----------------------------**

**AcaTrz1 (329) LVRKPWP------------GALTETLPPTTPSDISISYIIQHYDVRGKFNAAKAKALGVKQGPKFGQLTAGQ--SVVSEDGK----------------------------**

**AdeTrz1 (337) LVRKPWP------------GAMTQTLPPTTPSDVSISYIIQHHDVRGKFNPAKAIALGVKKGPKFGKLTSGQ--SVESSDGK----------------------------**

**PbrTrz1 (318) LVRRPWP------------GAMIQSLPPTTPSDISISYIIQHHDVRGKFNAEKAIALGVPRGPKFSKLTMGH--SVESTDGK----------------------------**

**AclTrz1 (302) LVRQPWP------------GANIEKLPPTTRGHKAVSYIVRNHDIRGKFDPVKAKALKVRAGPDFARLTKGE--KVLAEDGT----------------------------**

**AfuTrz1 (295) LVRQPWP------------GAGVEKLPPTTRCDEAVSYIVRNHDLRGKFDPQKAKELNVRAGPNFARLTKGE--NVLSEDGK----------------------------**

**NfiTrz1 (295) LVRQPWP------------GASVEKLPPTTRCDEAVSYIVRNHDLRGKFDPQKAKELNVRAGPNFARLTKGE--NVLSEDGK----------------------------**

**AcrTrz1 (302) LVRQPWP------------GAAVEKIPHTTWCQESVSYIVRSHDIRGKFDPKKAQELNIRKGPDYAKLTRGE--SVESLDGK----------------------------**

**AspTrz1 (303) LVRQPWP------------GAAVEKIPHTTWCQESVSYIIRNHDVRGKFDPKKAQELNVRKGPDYAKLTRGE--SVTSQDGK----------------------------**

**AflTrz1 (307) FVRQPWP------------GAAVEKIPPTTWCDEAVSYIVRNHDIRGKFDPKKAEELKVPKGKDFGRLTKGE--SVKSEDGQ----------------------------**

**AorTrz1 (307) FVRQPWP------------GAAVEKIPPTTWCDEAVSYIVRNHDIRGKFDPKKAEELKVPKGKDFGRLTKGE--SVKSEDGQ----------------------------**

**AteTrz1 (305) LVRQPWP------------GATVEKLPPTTWCDEAVSYIVRNHDIRGKFRPDVAKNLNVTPGPDYAKLARGE--SVLSKDNQ----------------------------**

**PchTrz1 (309) LVRKPWP------------GATVESLPSTSPSKESVCYIVRNHDIRGKFDPKKAQELKVEKGVKYAALTKGE--SVQSLDGK----------------------------**

**AniTrz1 (290) LVRKPWP------------GASISKIPTTTPCVESLCYIIKNHDLRGKFDAKKAIALNVKPGPDFGALTRGE--TVKATDGT----------------------------**

**PmaTrz1 (275) LVRRPWP------------AATLDRLPPTAPSSESLCYFVRNHDLRGTFDPKKAIELGIPKGPAFSQLTKGI--SYTTENGT----------------------------**

**TstTrz1 (279) LVRRPWP------------AATLDRLPPTTPSSESLCYFVRNHDQRGAFDPKKAIELGVPRGPAFSQLTKGL--SYTTEDGT----------------------------**

**AbrTrz1 (281) WTRTPWP------------GATILALPPTRPAPECVSYIVRSHPSRGQFDVARAKALGVKPGPDFGKLTNGQ--SVPNANGE----------------------------**

**CheTrz1 (281) WTRTPWP------------GAQILALPPTKPASECVSYIVRAQPIRGQFDVARAKELGITPGPKFGQLSKGI--SVQNASGE----------------------------**

**PtrTrz1 (281) WTRTPWP------------GAGILALPPTKPAPEAISYIVKSHPVRGQFDVASAKALGVTPGPDYGKLTNGI--PVKNAKGE----------------------------**

**PnoTrz1 (281) WTRTPWP------------GATIMAIPPTRPLPECLSYIVRTFPSRGTFDVARAKALGVKPGPDFGKLTSGK--SVQNEKGD----------------------------**

**MfiTrz1 (303) LVRRPWP------------GALIESLPDNKPAKVAISYIIRNHMQRGTFRPERAKALKVKSGPDYSKLAKGE--SVKNLDGE----------------------------**

**MycTrz1 (288) LVRKPWP------------GALVEKLPHTEPAKESISYIIRNHMQRGKFNPKRAMELKVPKGFAWSQLTKGE--NVENTEGQ----------------------------**

**SscTrz1 (279) LTRKPWP------------GALVESLPQTKPSTSSVSYIIKNYPQRGKFNAKEAERLNVKRGADYRTLVSGK--SVIATDGT----------------------------**

**CglTrz1 (380) LVRMPWP------------ASQIPELPPTKPSKQSMCYIVKCHPRRGKFDVNAATSLGVQKT-DFKKLTKGE--SVPGKDGS----------------------------**

**MthTrz1 (270) LVRLPWP------------AAQIEQLPPTEPSRQSMCYVVKCHSRRGKFNVQAATKLGVAKQ-DFKKLTSGE--TVTGKDGV----------------------------**

**TteTrz1 (270) LVRLPWP------------AAKIERLPPTSPAQRSMCYIVKCHPRRGKFNVEAATKLGVVKT-DFKRLTMGQ--SVPGADGT----------------------------**

**PanTrz1 (274) LIRSPWP------------AAKIETLPITKPSNESLCYIAKCHPRRGKFKPEEANKLGVSKF-DFKKLIDGE--TITLENGT----------------------------**

**NcrTrz1 (484) LVREPWP------------GALVHRLPPTDPCFDSTCYIVKNHARRGKFQPANALKYGLPKW-TFSKLAKGE--SVTAEDGT----------------------------**

**NteTrz1 (484) LVREPWP------------GALVHRLPPTDPCFDSTCYIVKNHARRGKFQPANALKYGLPKW-TFSKLAKGE--SVAAEDGT----------------------------**

**NdiTrz1 (484) LVREPWP------------GALVHRLPPTDPCFDSTCYIVKNHARRGKFQPANALKYGLPKW-TFSKLAKGE--SVTAEDGT----------------------------**

**SmaTrz1 (499) LVREPWP------------GALIHRLPPTDPSFDSTCYVVKNHARRGKFQPANALKYGLPKW-TFSKLAKGE--SVTAEDGT----------------------------**

**MgrTrz1 (360) LTRRNWS------------ASQIRNLPKTTPSSTSMCYIVRLQSRRGKFDTAAAERLGLEKK-LRGRVCMGE--SVTTASGK----------------------------**

**FgrTrz1 (272) WKLPTT--EDSEELRFNYPNLTHRPLPPTVYSRTSMCYLVKCLPRRGKFDAIKAKELKVPVV-DYKKLIAGQ--TIETEAGV----------------------------**

**FoxTrz1 (270) WVFPEK--EAQIDRSADIINVTHRRLPPTIYSQSSMCYIVKCLDRRGKFNPQKAKELGVHVT-DFRHLTAGS--TIVTKDGV----------------------------**

**FveTrz1 (273) WVFPEK--ETQIDRSGDILNVTHRPLPPTIYSQSSMCYIVKCLDRRGKFNPQKAKELGVQVT-DFKQLTAGN--TVVTKDGV----------------------------**

**NhaTrz1 (272) WVFPEH--ESLIDRNADLINVTHRPLPPTIYSQTSMCYLVKCHDRRGKFNPARAKELGVVVS-DFKYLTQGQ--SVTGKDGI----------------------------**

**TatTrz1 (277) WHFPELGEIGVDDRSENTLAINHFPLPRTTYGESSMSYIIKAHDRRGKFNAPVAKSLGVEPR-DFKLLTAGQ--SVQGKDG-----------------------------**

**TviTrz1 (275) WHFPEIGEVGVDERSNNALAINHFPLPRTTYGETSMSYIIKAHDRRGKFNAPVAKSLGVEPR-DFKLLTAGQ--SVQGKDG-----------------------------**

**TreTrz1 (281) WHFPEIGETGVDERGDNVLQINHYPLPRTAYSEMSMSYIVKSHGRRGKFNAPMAKSLGVEPR-DFKLLTAGE--SVQGKDG-----------------------------**

**AgoTrz1 (185) DTML----------------------------QVSTSYEITFNPVRGKFDAKEAARLGVPGGPVRRELAEGR--SITLADGT----------------------------**

**CanTrz1 (187) NISVP---------------------------SETTNYEVQFNSIRGRFKVEEAIRLGVPKGPLFAKLTKGE--SITLEDGT----------------------------**

**SceTrz1 (186) LDAKV---------------------------EVSTNYEISFSPVRGKFKVEEAIKLGVPKGPLFAKLTKGQ--TITLDNGI----------------------------**

**ZroTrz1 (185) QFSVP---------------------------RTSTCYEIVFEPVRGKFRPDIAAKLGVPKGPSFGKLASGQ--SITLDDGS----------------------------**

**VpoTrz1 (189) LSYTY---------------------------NSTQSYEISFHPIRGKFKVKEAINLGIPKGPLFSKLANGE--SITLEDGT----------------------------**

**LthTrz1 (186) EYAIP---------------------------KQSTSYEINFNPIRGRFKVEEAMRLGVPKGQLFAQLTKGL--SVTLPNGD----------------------------**

**KlaTrz1 (188) EAIGAK--------------------------NLTTSYELTFPAVRGKFNVEEAKRLGVPKGRLYAELAKGN--SITLEDGT----------------------------**

**PpaTrz1 (182) QVKLP--------------------TVDGLSNSFSTNYLIDFHPVRGKFNVKKAIELGVPKGKTFAQLTKGED--VLLPDGVT---------------------------**

**CalTrz1 (191) HVHLP-------------------SASEIATTQQSISYCISFVPVLGKFDPKKAKELGLTPGPQFRDLTNGL--SVVNKNGD----------------------------**

**CduTrz1 (191) HVHLP-------------------SASEVVTTQKSISYCISFVPVLGKFDPKRAVELGLTPGPQFRDLTNGL--SVVNKNGD----------------------------**

**CtrTrz1 (191) HVHLP-------------------KASEIVTSQAAYSYSVRILPIRGKFDPKRAIELGLKPGPQFRDLSNGK--AVVNENGE----------------------------**

**CpaTrz1 (195) HVQLP-------------------EASKLITQQDSISYCIEFVPIPGKFDAKKAKELGLKPGPVFKELVAGK--SAVNEAGE----------------------------**

**CguTrz1 (190) HVKLPDP-----------------HASSLVQNQLAINYLIRFLPIRGKFDPVKAKALGIKPGIDFRKLTQGS--TVYNESGE----------------------------**

**PguTrz1 (190) HVKLPDP-----------------HASSLVQNQLAINYLIRFLPIRGKFDPVKAKALGIKPGIDFRKLTQGS--TVYNESGE----------------------------**

**DhaTrz1 (191) HVKLPNP-----------------FNLLPTYQQPSLNYLVRFLPIRGKFDPIKAKSLGIKPGIDFRKLTQGH--SIVNEQGE----------------------------**

**PstTrz1 (192) HVRIPEP-----------------QALLPTAQQSAVSYLVRFLPIRGKFDPVKAKSLGLQPGANYRKLSQGV--AVENDQGV----------------------------**

**CluTrz1 (188) HVRLPEP-----------------SELVDVGSQPALSFVIRFLPVRGKFDPVKAKALGVEPGINYRKLTMGD--SVLNSKNE----------------------------**

**YliTrz1 (213) YNRKELQ------------KQSDTALPAVDLDERSTCYIAQLHQKKGKFNVEAANALKVP-RYDFKQLIAGN--NVTLEDGS----------------------------**

**AmaTrz1 (247) WLQGKWQG-----------QLQGVQPIFGKQVEASVAYVCFGPEVRGKFDVKRAAELGLKPGRAFGALLKRLIANVFVSFFGAHLMLVRGLGKLQRGESVTMPD------**

**BdeTrz1 (203) --------------------YNWDQLPSTADDAVSLCYICQGPEIAGTLDPVLAMKLGVKRGPDMGKLKSGQ--TVLSINGVP---------------------------**

**SpuTrz2 (227) VPRTEIG------------RLGSARLPRTEPDHVVLSYICRGPNVPGKFNPVAAQKLGVKPGKDFGILAGGQ--TVTTADGT----------------------------**

**SpuTrz1 (269) VPNIALG---------KRDHTKLVRLPMAIPSKTVTCFICQGPPVRGKFDPDKATALGVPPK-EFGTLHRGD--SFLNDKGE----------------------------**

**ScrTrz1 (236) YNT--PP-----------PLENQSPQIF-------ISYIVQSHPSLGKFDAKKAKSLGITKGTDYGKLAKGE--SITLENGS----------------------------**

**SocTrz1 (235) YNT--PP-----------PLEEQSPQIF-------ISYIVQSHPSPGKFDAKKAKSLGITKGVDCGKLAKGE--SVTLDNGS----------------------------**

**SpoTrz1 (252) YNT--PA-----------PLEPDKPELF-------ISYIVQSHPTPGKFDAAKAKSLGITKGLDCGRLARGE--PVTLENGK----------------------------**

**SjaTrz1 (210) AVP--QS-----------DAYNEGIDTH-------FSYVVQSHPSPGRFDAKKAKSLGITKGADCGKLARNE--NVQLPDGT----------------------------**

**ScrTrz2 (207) -T-----------------V-----------------------PMHGTFNSEKAKLLGVPFGKANGLLCQGKS-VLASDNKT----------------------------**

**SocTrz2 (207) -T-----------------V-----------------------PIHGTFDAVKAKLLGVPFGKANGLLCQGQS-VLASDNKT----------------------------**

**SpoTrz2 (201) -S-----------------K-----------------------EAAGVFNADKALALGVPFGPSNGKLCAGEA-VLSKDGTT----------------------------**

**SjaTrz2 (176) -----------------------------KRHNSWCSLHVSTPVATGVFDVEKAIQLGVKPGPAFGLLTKGQ--SVLSSDGFTR--------------------------**

**CneTrz1 (228) RVSNVFLAPDGTINAARPDPRAPLPLPSDADVETQMVYICQAPDVRGKFDVAKATALGVPNGPLRGKLTRGESIEVSDPNVEGG--------------------------**

**TmeTrz1 (216) PIRPNS-----------ADARYPLGLLDDSVQRCDMIYVFQAPDFPGKFDVASAERLGVY-GKARGLLVKGQTVEVKDPSQPGG--------------------------**

**AbiTrz1 (224) NFRLQLP-----------SFS-P---PSTFPLSSTLAYVVVGPRTRGKVDGKKLDELNVPRNSLRGKLSRGETIQFEVKGPSG--------SE-----------------**

**CciTrz1 (313) GYHTQLP-----------SLPPNFQRT-P------IGYVIIGPRIRGRFDVARATALGVPRGPLRGLLTKGETVTFQVEETFTNVQGQQETRMK----------------**

**ScoTrz1 (268) GFHAQLP-----------QPVNVDPSTSRTP--ATLAYVVLGPRIRGKFDAAAATKLGVPFGPLRGDLAKGLNVTVKVQNEAG--------ETI----------------**

**LbiTrz1 (261) GFHMQLP-----------AFSRTSDVQ-------ALAYVVIGPRVRGKFDVEKAVELGIPFGPLRAKLTKGESITFKVKRGD---------EEF----------------**

**PosTrz1 (252) GFHQQLP-----------RFAYDVPVNGPP----TLAYIIAGPRIRGKFDVKRAEALGVPFGRLRGQLTKGQAVTFDVNVGD---------NVE----------------**

**SlaTrz1 (278) GFNKPLP-----------PLEFNYTDSTDISSKPVVAYAVVGPRVRGKFDVKKAEALGLKPGPLRSKVTRGETVTVMVDDGLGQG------KMM----------------**

**HanTrz1 (270) YH--QLP-----------THTDPAPG-------SSLAYVVVGPRVRGRFDAKRAQELGLQ-GRLRGRVARGETVTYTVDDGKG--------GQV----------------**

**PplTrz1 (293) HRGRRLP-----------PFAHPDSGP------ATLCYIVVGPRVRGKFDAARANALGVQ-GKLRAPLTQGQSVSFMVDDGTG--------NMV----------------**

**PhaTrz1 (282) HINAQLP-----------KLDVPRTS---------LCYVCVGPRARGKFDNEKAKQLQIPRGRIRSSLTNGQTISFMVDDGNG--------GQT----------------**

**AbiTrz2 (246) EVLEDEP-------------------------IADDSERWVPTWLHGNRLPFFSFPKTFPQSSTLAYVVIGP-------RPE----------------------------**

**MglTrz1 (212) PARLATSPARCAYALPPPLVPCIQGGADAGRQAAVMAYICSGHTQRGKFDPARASELGIPPGPEFARLSRGEQVRIIRPVAWSTMDAEQRQEWLRSCRRNGSGSNNNNHS**

**MlaTrz1 (239) YSYDKLE-----------APTSPISSE-------PSSYFIVGPKHRGKFLPNKAKELGVKPGPDFSKLAKGED--LVLKDGR----------------------------**

**PgrTrz1 (229) YSYQRLQ-----------EPDEPLNTD-------PVSYLVIGPRLRGKFLPEKAKQLGVKPGPNFAKLVNGES--VQVNGET----------------------------**

**AbeTrz1 (368) -------------------TITPDMVLGETRPGKGFAVL---ELPSVDYIQSIINRKEWDNAE--------------------------------VMKGMEVFIWILGPG**

**TruTrz1 (366) -------------------TITPDMVLGETRPGKGFAVL---ELPSVDYIQSIINRKEWENAE--------------------------------IMKGMEVFIWILGSG**

**MgyTrz1 (366) -------------------TITPDMVLGENRPGKGFAVL---ELPSVDYIQAIVNRKEWENAE--------------------------------IMKGMEVFVWILGPG**

**McaTrz1 (367) -------------------TITPDMVLGENRPGKGFAVL---ELPSTDYIQSVVNRKEWDNPE--------------------------------VMKGMEVFIWILGPG**

**CimTrz1 (369) -------------------TITPDMVLGESRIGKGVAII---ELPSVDYVENLIARPEWKSTE--------------------------------VMNGMTAFIWILGKG**

**CpoTrz1 (369) -------------------IITPDMVLGESRIGKGVAII---ELPSVDYVENLIARPEWKSTE--------------------------------VMNGMTAFIWILGKG**

**UreTrz1 (372) -------------------TITPDMVLGESRIGKGVAII---ELPTVDYIENLLSRPEWQSPE--------------------------------VTKGLVVFIWILGKG**

**AcaTrz1 (397) -------------------VITPEMVIGESQPGGGIAFL---DIPTADYVENLVNRPEWTTPE--------------------------------VMRGLTTFVWILGPG**

**AdeTrz1 (405) -------------------IITPDMVLGESQPGGGLAFV---DIPTPDYVENLVNRPEWTTPE--------------------------------VMKGLASFIWILGPG**

**PbrTrz1 (386) -------------------TITPEMVLGEPQPGGGIALM---DLPTADYVENLLNRPEWTTPE--------------------------------IMRGFTTFIWVLGPG**

**AclTrz1 (370) -------------------TVSPDMVLGPPRLGKGLAII---DLPTPEYVDDLVGRPEWNSPA--------------------------------VTTQLEAFIWILGPG**

**AfuTrz1 (363) -------------------TVTPDMVLGPPRLGKGLAII---DLPTSDYVDDLVDRPEWNSAS--------------------------------VTRGLEAFLWILGPG**

**NfiTrz1 (363) -------------------TVTPDMVLGPPRLGKGLAII---DLPTSDYVDDLVDRPEWNSAC--------------------------------VTTGLEAFLWILGPG**

**AcrTrz1 (370) -------------------IVTPDMVLAPTRLGKGMAII---DLPTPAHVDDLVNRPEWNSPA--------------------------------ATTSLEAFLWILGPG**

**AspTrz1 (371) -------------------IVTPDMVLGPSRLGKGMAII---DLPTPAHVDDLVNRPEWNSPA--------------------------------VTTGLQTFLWILGPG**

**AflTrz1 (375) -------------------TITPEMVLGPPRLGKGLAII---DLPSPEYVESLISRPEWKSPS--------------------------------VTSNLEAFIWILGPG**

**AorTrz1 (375) -------------------TITPEMVLGPPRLGKGLAII---DLPSPEYVESLISRPEWKSPS--------------------------------VTSNLEAFIWILGPG**

**AteTrz1 (373) -------------------TITPDMVLGPTRLGKGLAII---DLPTPEYVDGLVNRSEWNSPS--------------------------------VTTNLEAFLWILGPG**

**PchTrz1 (377) -------------------TITPDMVLGQTRPGKGVAIM---EVPSSEYVEDLVSRAEWKSPA--------------------------------VTTELQAFIWILGAG**

**AniTrz1 (358) -------------------MVTPEMVLEPTKPGKGLAVM---DLPTSDYVESLLNRPEWKSPS--------------------------------VASNLAAFFWILGPG**

**PmaTrz1 (343) -------------------VVTPDMVMGPTRRGRTVAFI---DIPSTFYIDDLISRPEWKSPT--------------------------------LTETLASVFWILGPG**

**TstTrz1 (347) -------------------VVTPDMVLGPTRPGRTVAFI---DLPSTLYIDDLISRPEWKSPT--------------------------------LTETLSSVFWILGPG**

**AbrTrz1 (349) -------------------MITPDQVMGADKPGQGIAVL---DIPSVAYVESILQREELSSPE--------------------------------VMTGIEAIVWMLGPG**

**CheTrz1 (349) -------------------WVTPEQVMGADRPGQGIAIL---DVPSVDYVDSIVQREELNSPE--------------------------------VMNGIGAIIWMLGPG**

**PtrTrz1 (349) -------------------LIHPNQVMGPDRPGQGIAIL---DVPSVAYVESIVRKEELSSPD--------------------------------VMTGIRAIVWMLGPG**

**PnoTrz1 (349) -------------------WIEPSQVLGADRPGQGIAIL---EVPSLEYLEAVVQREELNNSQ--------------------------------VMAGIGAIIWVLGPD**

**MfiTrz1 (371) -------------------TVTPEQVMDPPRVGGGIAVV---DLPEPEYIENLVHRPEWRTKQ--------------------------------VMEGVGAMIWICGKD**

**MycTrz1 (356) -------------------TITPEMVLEPSRVGGGLAVV---DLPSAEYIDNLVARAEWKEPK--------------------------------VMEGVGAIVWICGKG**

**SscTrz1 (347) -------------------IVTPEQVLGESKEGSGVAIL---EVPDVSYIKGLLAREEWKSEE--------------------------------VMAGVELVIWILGRG**

**CglTrz1 (447) -------------------VVTPNMVMGDQIEGRGFAVI---DLTSQDLIDDLLNRHEWSNPE--------------------------------IMQGIDTVYWILSDG**

**MthTrz1 (337) -------------------VVTPDMVLGPPIEGQGFAVV---DLPSEDLVEDLLSRPEWSNPE--------------------------------IMKGVSAMFWILSSG**

**TteTrz1 (337) -------------------IVTPDMVMGAQIDGRGFAIL---DIPFPDLIDPLLQRHEWSNPE--------------------------------IMKGIDTMYWILSDD**

**PanTrz1 (341) -------------------VVTPDMVMEPTVAGRGLAVI---DIPAEDMVNSFLARPEWSDSA--------------------------------LMSGIDVMYWISSEQ**

**NcrTrz1 (551) -------------------VVTPDMVLDPPIPGHGFALV---DVRYEYLLDSLLKRPEWENKE--------------------------------IMEHIDAFYWILSP-**

**NteTrz1 (551) -------------------VVTPDMVLDPPIPGHGFALV---DVRYEYLLDSLLKRPEWENKE--------------------------------IMEHIDAFYWILSP-**

**NdiTrz1 (551) -------------------VVTPDMVLDPPIPGHGFALI---DVRYEYLLDSLLKRPEWENKE--------------------------------IMEHIDAFYWILSP-**

**SmaTrz1 (566) -------------------VVTPDMVLDPPIPGHGFALI---DIRYEYLLDSLLRRPEWENKE--------------------------------IMENIDAFYWILSP-**

**MgrTrz1 (427) -------------------VITPEMVMAPPVPGVGFAVI---DVPSADYVESLISRPEWDNEK--------------------------------IMSDIVAIYWILGKG**

**FgrTrz1 (349) -------------------TVTPEMVVGSDIPGHGFIVA---DIESHDLIDSFMGRLEWSSS---------------------------------LMDDVVAVYWILGP-**

**FoxTrz1 (347) -------------------TVTPEQVLGETQPGQGFILA---DIESRDLIDSFMERPEWSNSE--------------------------------LMSHVAIVYWILGP-**

**FveTrz1 (350) -------------------TVTPEQVLGETQPGQGFILA---DIESHDLIDSFMERPEWSNTE--------------------------------LMSHVAIVYWILGP-**

**NhaTrz1 (349) -------------------TVTPDMVLGEMQLGNGFIVA---DIESRDFLDSFFERPEWSNTE--------------------------------LMANVVTLYWILGT-**

**TatTrz1 (355) -------------------LVTPEMVLGETQRGKGLVVA---DIGSQDFIEAFMERPEWENQE--------------------------------LMENIAVMYWIVGP-**

**TviTrz1 (353) -------------------LVTPEMVLGETQRGRGIIVA---DIGSQDLVEPFMERPEWKNKE--------------------------------LMENVAVMYWILGP-**

**TreTrz1 (359) -------------------LVTPEMVLGEPQPGRGLIVA---DIASQDLVEPFMARPEWKSAE--------------------------------LMDNIAVMYWILGP-**

**AgoTrz1 (237) -------------------VITPEQVVHKQREFARVLIL---DVPSDDYIPAFFEKFADYNVEG-----------------------------------LGAVYYLLGSE**

**CanTrz1 (240) -------------------IVSPDQVLEKERNFGKVLIL---DIPDNSFIPSFMEKFKTHDLSG-----------------------------------TAAIYYFLDDK**

**SceTrz1 (239) -------------------VVTPEQVLENERHFAKVLIL---DIPDDLYLNAFVEKFKDYDCAE-----------------------------------LGMVYYFLGDE**

**ZroTrz1 (238) -------------------QITPEQVLEKQRQFPRVLIL---DIPDDSYIEGFREKFKEYDCED-----------------------------------LGIVYFFLGGD**

**VpoTrz1 (242) -------------------VILPEQVLEKERNVSKVLIL---DIPNDNYIPHFKEKFKNYDFED-----------------------------------LSVIYYFLDED**

**LthTrz1 (239) -------------------VVKSEQVLEKQRSFAKVLIL---DIPSDSYWPQFLEKFQVYEKSS-----------------------------------LGAVYYFLGEG**

**KlaTrz1 (242) -------------------VINSDQVLSETRNFGKVLVL---DIPSNKYIPRFIDEFKNYPTDQ-----------------------------------LSAIYYFLGDE**

**PpaTrz1 (243) -------------------VVKSSDVVEESKSFPKLLVL---DIPSDEYLEPTLHNPLIVSSS----------------------------------QDIGIVYIFLNEK**

**CalTrz1 (252) -------------------TITPDQVIAPNRVFRKILVI---DVPSNDYYENTINSDRWFEHDHPK-----------------------------NAGEVGLVYHMLGQD**

**CduTrz1 (252) -------------------IIAPDQVIAPNRIFRKILVI---DVPSNDYYENTINSNRWFEHDQTK-----------------------------NAGEVGLVYHMLGQD**

**CtrTrz1 (252) -------------------TIQPEQVIAPDRLFRKLLII---DIPNNDYYENTITSNKWFAKAENE-----------------------------KSNEIGLVYHFLGEE**

**CpaTrz1 (256) -------------------TILPSQVVGPDRILPKVLII---DIPSEAYYEPTVTSYQLQEIEN-----------------------------------VGLVYHFIGDD**

**CguTrz1 (253) -------------------PVYPEQVIEPSKHFSKALIL---DIPDPSYLHTAINTNDWFEKNEEV-----------------------------GEEAIGIVYHFLGDD**

**PguTrz1 (253) -------------------PVYPEQVIEPSKHFSKALIL---DIPDPSYLHTAINTNDWFEKNEEV-----------------------------GEEAIGIVYHFLGDD**

**DhaTrz1 (254) -------------------TVHPHQVIEESKLFLKLLIV---DIPNASYLSNTLNCEEWFRSNENI-----------------------------GEEDIGIVYHFLGDD**

**PstTrz1 (255) -------------------LIQPQQVVADPKTFPKLLIL---DIPNKSYLDNSLGSDKWFEKNDDL-----------------------------GEEEVGIVYHFLGDD**

**CluTrz1 (251) -------------------LVHSHQVLAEPKSFRKLVII---DIPNANYLENTLRSDEWFLQSEEA-----------------------------GQELPGLVYHFLGDD**

**YliTrz1 (280) -------------------VIKPEQCIGEPSNYGRILFL---DIPDARYVDGVINCPEWTRKYNTRDAQPVYGAGQKRDSGEMNEEVKTPDVSNVLEPFVSTVYHFFGPE**

**AmaTrz1 (340) -----------------GKVIHPLDVMGPTRPSQVFVVL---DLPTEDYLRGALASDTLFQRIG---------------------------------DSPALVFHMLGAN**

**BdeTrz1 (264) --------------------ISPDQCVTPRRPGPVFLIL---DCPSKSYIASLIENAIIKQAAS-------------------------------GEKPAQCVVHICGDK**

**SpuTrz2 (295) -------------------VVYPHMCMGEPRPG---AIFVIIDCPSPAYINALVNSPEFAPFYDTTN-----------------------------GNHVRCVVHITGDG**

**SpuTrz1 (339) -------------------RVDPHQCMGPDRPGS---VFFIIDCPSADYIGTLVNNPRFVPHYAS--------L---------------------TNNPVAVIVHMLGDD**

**ScrTrz1 (296) -------------------TVYPDDVIGPP-------------VPGSTFFFIHCTHASLIDSLIQQ----PQWA---------------------NAPEPICIIHSVSED**

**SocTrz1 (295) -------------------TVYPDDVIGPP-------------IPGSTFFFIHCTHESLIDTLIQQ----PQWS---------------------TAPEPVCIIHSVTDA**

**SpoTrz1 (312) -------------------TVYPKEVIGPS-------------IPGSSFFIIHCPNELVIDLVIEN----HKWT---------------------NAPKPVCVIHSVTPE**

**SjaTrz1 (270) -------------------IIKPSDVIGPD-------------VPGLCFYFVYCPSLNWLEATLEH----SAWS---------------------RAPKPVCIIHAVSQD**

**ScrTrz2 (247) -------------------WVRPEEVLGPT-------------RPAQKFVIIGCSSPSALENLINY----ANTW---------------------EHELPSCIIHVLDKG**

**SocTrz2 (247) -------------------LVRPEQVVGPT-------------KPGQKFVVLGCGSRTAINDLLHY----VNTW---------------------EQDFPSCIIHVLDKG**

**SpoTrz2 (241) -------------------WIYPHQVVGPP-------------RKRQYFYVLGCSSLSALNQMSKH----VDSF---------------------SDVYPTCIIHILEKG**

**SjaTrz2 (229) --------------------VHPEQVLGSPKPPKNLVLLGTRDISETAFDDALNCLKLNFIPS-----------------------------------KNDCVVHILPRG**

**CneTrz1 (312) -----------------IRVVKPEDCLIGGGKGGTLVIVNCTEKTLQALLDCEALRQWQKKSP----------V---------------------GAKEGEGEVDVMVHR**

**TmeTrz1 (288) -----------------MRIVRPEECLSGGGTGAVMVIVQCTEKTVSSLLGSSQLNDYKHPGG---------------------------------GKTVALMVHHTSKD**

**AbiTrz1 (294) -----------------MRTVRPEDCVGLSEPPAVVIIL---DVPMIEMIPSVIHSFDSAYKPFRSIDP--SDM---------------------EKHTVRVVYHLLGEG**

**CciTrz1 (389) -----------------DVTVKPEQCVGESEAPSAVVVL---DVPEKRYLGSLEKWFAEGVIGRVR-SRKEEDE---------------------KEYSVKAVFHMLGEG**

**ScoTrz1 (341) -----------------ERVVQPSDVMSESIPPSAVLIF---DVPSSAYIPSLVNSFTNAPLYSRLRSRKEEDT---------------------KDYAIRAIFHKLGKG**

**LbiTrz1 (328) -----------------ERTVKPEECIGPSETPGVIIVL---DVPSTHHIPDLVSSFKDSEFFRQFLSKVPGTS---------------------DSYAVRNIFHLCGDN**

**PosTrz1 (322) -----------------KRTVRPEECVGESESPGVLIIL---DVPTPAYIPSLISSFTTSSTFARFRSHQPEDA---------------------KEYNVRTVFHLCGKG**

**SlaTrz1 (355) -----------------ERTIRPEDCMGQSESPGVMLIL---DTPTPSHIPLLTSAFTRSSLFTSFRSREVRSQ---------------------EDHAVRAVFHICGNH**

**HanTrz1 (335) -----------------ERTVRPEDCVGESESPGVVMIL---DVPSQAHIPDLTWSFDS-PFYSTFRSKDAEDL---------------------KKYNVHIIYHLLGEG**

**PplTrz1 (361) -----------------ERTVMPEEVVGPSEQPHVTMIL---DVPTPEHIPELVASFTENPFYARFRSKAEEDA---------------------KEYHVHAVFHLCGPG**

**PhaTrz1 (348) -----------------ERTVRPEEVIGPSEAPKVVMIF---DVPTRDHIPSLVSAFETSPFYKSFLG--TEHA---------------------DEHVVRCIHHLCGEG**

**AbiTrz2 (296) ---------------------KPSAKDIPPQPHPVVIIL---DVPTPDFIPSVLRMFDNECAPFLYTDPHHG-----------------------GNYKIKAIYHMLGRR**

**MglTrz1 (322) RSGKEASQQDFSHTDVEYVDIQSQDVVGSARAGPVFFYMH---VPTLQHLDSLLDDPKIRAAFAPYTWETNKSL---------------------IEEQRRTPHMILHAV**

**MlaTrz1 (301) -------------------IIEGKMCLEGG------------SDGSAFYISNVANKHQLNSTALPQLQEMQDLA---------------------QGAELTTIFHFVGTD**

**PgrTrz1 (291) -------------------VVTSDMCVEGG------------SAGSAFFISSIGSMEQLGRTTLVDPACISRVS---------------------DGANLCAVYHLVHED**

**AbeTrz1 (424) --VGGHPLLQEFVTKMS----KYKHIVSSPDYCADNYGFPAVSTATLELSAIDGDRYQKTFYNGEAMLPVSAFAA-----------ADTKTSVSIAQPNLTIDLEPSVKL**

**TruTrz1 (422) --VGGHPLLQEFVTKMS----KYKHIVSSPDYCADNYGFPAVSTATMELSAIDGDRYQKTFCNGEAMLPVSNFAA-----------ADTKTSISIAQPNLTIDLEPSVKL**

**MgyTrz1 (422) --VGGHPLLQEFVAKMS----KYKHIVSSPDYCPDNYGFPAVSNATVEFSAIDADRYQKTICNNKAMSPVNDFVA-----------TDTKTSISIARPNLTIDLEPSVKL**

**McaTrz1 (423) --VGGHPVLREFVAKMS----KYKHIVSSPDYCPDSYGFPAVSNATIEFAAIDGVRYRTPFCNNEAKASVNTFAS-----------ADTKDSINIAQPNLTIDLEPSVKL**

**CimTrz1 (425) --VGAHPRLQEFISTMS----QAQHVFSSPDYSPNDLTFRAVARSTMEFSEIDSARYSPPKYVTSPRSN---VP---------------RPEICVAKPGMVVNLEPEFSI**

**CpoTrz1 (425) --VGAHPRLQEFISTMS----QAQHVFSSPDYSPNDLTFRAVARSTMEFSEIDSARYSPPKYVTSPRSN---VP---------------RPEICVAKPGMVVNLEPEFSI**

**UreTrz1 (428) --VGSDQKLQEFISTMP----QAKHIFSSPDYSHNELTFRAVARSTIEFSEIDSTRYSPPNCSDCHGLK---PL---------------NSDIRIAEPGAVVDLEPQFDI**

**AcaTrz1 (453) --VGSHPLLQEFVVKMS----QYKHIVSSMDYCSNYLAFPFGSTETTHFSVLDNERFHVPHHDNVELPQKTFLTLPSSSPPSRPASDPLKSVFLPALPNLQVELQPKFLI**

**AdeTrz1 (461) --VGSHPLLQEFVSKMS----QYKHIVSSPDYCPNYLAFSLGSTETAHFSFIDNERFHLPHHNNEEVPQKTFLTP--GPLPDSSTKDPLKSVFIPARPNLRVELQPKFQI**

**PbrTrz1 (442) --VGGHPLIQKFVSKMS----QYKHIVSSPDFCPNYLAFPYSSMETSQFSVLDSARYSVPHHDNVVVPQKTFLTS---SAVPTPTADALKSAFEPAVPGLQLELQPKFVI**

**AclTrz1 (426) --VGDHPRLREFVARMS----HCKHTVSSTDYCPNYLALGSVAGSSIRLARLRRENYPIPYHDNQTLPQLGTSTC----DSETTKAMVRNSPFESIKPGLVIDMEPKFEL**

**AfuTrz1 (419) --VGDHPRLREFVARMS----HCKHTVSSTDYCPNYLALSSVAGSSIRLARLRGENYPVPVHDNKTLPQPGTPTS----ESETTKEMIRNSPFEPVKPGLVIDMEPKFEL**

**NfiTrz1 (419) --VGDHPRLREFVARMS----HCKHTVSSTDYCPNYLALSSVAGSSIRLARLRGENYPVPVHDNKTLPQPGTPTS----ESETTKEMIRNSPFEPIKPGLVIDMEPKFEL**

**AcrTrz1 (426) --VGDHPRLHEFVARMS----HCKHTVSSSDYCPNYLAMKSIATSTTRMGFLRPESYPTLVHDNVTLPQP--GTT----SSDSARDAAKPVSLQPVAPGVIVDMEPQFAL**

**AspTrz1 (427) --VGDHPRLHEFVARFS----KCEHTVSSSDYCPNYLSMKSIATSTTRMGLLRPDNYPTLVHDNVTLPQP--GTR----TAEPTTDTTKAVALQPVVPGVIIDMEPNFAL**

**AflTrz1 (431) --VGDHPRLREFVASMP----QCKHTVSSSDYCPNYLAMGSIAGSSVRMAQLRRDNYPVPVHDNVSLPQPGTRTH----GSEVTVRNVQNSPFEAIEPGLIIDMEPNFDI**

**AorTrz1 (431) --VGDHPRLREFVASMP----QCKHTVSSSDYCPNYLAMGSIAGSSVRMAQLRRDNYPVPVHDNVSLPQPGTRTH----GSEVTVRNVQNSPFEAIEPGLIIDMEPNFDI**

**AteTrz1 (429) --VGDHPRLHEFVAKMS----HCKHTVSSSDYSPNYLSLASVAGSSVRMAQLRKENFPVPVHDNVTIPQPGTPTA--------GTEIAKIPSFEPVEPGLVIDMEPKFVL**

**PchTrz1 (433) --VAEHPKFQEFVARMS----HCKHTVSSTDHCPNYLALTSCASSTVRLARLKGDSYAVPVHDNMSLPQPGTSNA----NSKSAIAARQNSPLQPLEPGFIIDMEPSFGL**

**AniTrz1 (414) --VGEHPRLREFVASMP----NCKHIVSSTDYCPNYLTMQSVAGSAIRMARIRPDNYLVPIHDNNSVPQT--QSS----LEDRMAVDSQNHSFERAEPGLIVSMEPEFKI**

**PmaTrz1 (399) --VAEEPRLLDFISSLK----NCEHVVSSTDVCPNRLTMVTAAKSSLRLAKINSDNFIIPYHDNVTVPQAGTRVL----RHKSEPSAAQKPLWTVSRPGLLLDMEPKFGI**

**TstTrz1 (403) --VAEDSRLLDFISGLK----GCEHVVSSTDVCPNRLTMMAAAKSSLRLAKINSENFAIPYHDNVTVPQPGTRPN----GHNSNASTTQKPDWTVSCPGLLLDMEPNFGI**

**AbrTrz1 (405) --VADHPTLVKFMNKMD----SIEHVVSSIDTAPNRIANDSVAAQATRLGQIDPTRYSTPVFDNTSLPQKSLYRT------GTPATSLLPNGAIAADRGMSFTLMPKFVL**

**CheTrz1 (405) --VAGHSTLTEFMKKLR----DVEHVISSIDTAPNRMTNDSVAIQATRLGQVDPARYSTPVFDNTSVPQKSIYRA------GQSASSELPKGVIAADRGLSFALMPRFTM**

**PtrTrz1 (405) --VAGHPVLTEFMEKLS----DVQHFVSSVDTAPNRVSFNSVAGQATRLNLIDPKRYSTLVFDSTEVPQKTVHRT------TPARTSSLPPNIFAADRGMTFTLMPKFEA**

**PnoTrz1 (405) --ISGHPILSEFMDKLS----DVQHIISSPDVSPNRIAHDSVACQATRLGQVDPARYSVPIHDNFTVPQEGLFSN------SQRHISPMPKESIRADRGLSFTLMPKFAT**

**MfiTrz1 (427) --VAKDPKLHAFMQEFQ----HLKHIVSSPDCCPNNISLDSVAGSTIRLKEVDPERYIVPVHDDPLSLQTRQVGD--QKTSVANTAHTLPEAVHVAQRGQVVQLEPAIEI**

**MycTrz1 (412) --VAMDSRLHAFMKEFE----HLQHIVSSSEYCPNHIALDSVAASTVRLKQVDPDRYTVPVHDAADGKSTYGGGN---NFLTTREKHPLPKGVHIAARGHEVQLEPTIEY**

**SscTrz1 (403) --VLADPSIQKFMEEYK----KFTHIVSSQDNSPNYLTFSSAAAQAIRLHLLDSERFPIPSFSNASEPPSQ------------------PVPYLRAKLGKTMLLEPKFEI**

**CglTrz1 (503) ITLESPG-VSQFIKTLS----SAKHIVLGSAVCPNVPALESPTSQLIKMNSIDRDRFPLPVFDSQPTSELGQEL---------------ESVAELARAGLKYQLAPKPSF**

**MthTrz1 (393) VTLEKDQRLAQFVRARS----GIRHIVLSSSLCP--------------------------------------N-----------------AVAEVGRPGLKFQLAPKSTF**

**TteTrz1 (393) ITVSNDDRVQQFMKTHA----SIKHIILGSAMCPNVPGLESPTSQLIKMNGIDPQRFPLPVFDTKPRSDLDKEL---------------ELIAEVGRPGLQYQLAPRATF**

**PanTrz1 (397) FSAYRDERLVEFMKKFL----RYSTFCSARIRPLAPVG-----------------------------------------------------AGDVGAAGARINLHPKPGP**

**NcrTrz1 (606) -EVKDDSRLKDFMAKHS----SFKHVVLGEGMNPNTISFGGASGKAIMMHRMDPDRFSIPNHNNQEEALPA-GL---------------ASVAQLGKPGERLLLSPSVEF**

**NteTrz1 (606) -EVKDDSRLKDFMAKHS----SLKHVVLGEGMNPNTISFGGASGKAIMMHRMDPDRFSIPNHNNEEEALPA-GL---------------ASVAQLGKPGERLLLSPSVEF**

**NdiTrz1 (606) -EVKDDFRLKDFMAKHS----SLKHIVLGEGMNPNTISFAGASGKAIMMHRMDPDRFSIPNHNNQEEALSA-DL---------------ASVAQLGKPGERLLLSPSVEF**

**SmaTrz1 (621) -DVKDDSRLKDFMGKHN----SFKHIVLGEGMSPNVISFAGASGKAIMMHRMDPDRFSIPNHNNKEGDLPA-GL---------------ASVAELGKPGQRLLLSPSVEF**

**MgrTrz1 (483) --LAADERILAFMRKHS----GLRHTVSSLDVCANHVVFEASTKGMIRHNYLDKKRFPLPVFDNAGPMSNLSS-----------------KPFVPAKPEVRLELAPRQVF**

**FgrTrz1 (403) -GLAGDARIQKFVDEHP----TLKHFFCAKDTCPNMISLAGPAQLQTKLRVIDPDRFSLLDFDNHVRGVLPS-----------------GPQVQSGRTGNKINLMPRLRF**

**FoxTrz1 (402) -GIADDARIQKFVDEHP----TMKHFFCAQDTCPNMISLAGPGQLQTKLRRIDPERFTLLKYDNDIKGAMPN-----------------GSQVESGRIGNKISLMPRLKF**

**FveTrz1 (405) -GMADDARIQKFVDEHP----TMKHFFCAQDTCPNMISLAGPGQLQTKLRRVDPERFTLLKFDNDVKGAMPN-----------------GSQVESGRTGTKIALMPRLKF**

**NhaTrz1 (404) -GLANDARIHQFIQEHP----NLKHVFCAQDTCPNMIALAGPSELQTKLRRIDPERFSLLKYDNTVKGDIPP-----------------GLKVEHGRIGSKMSLMPRLRF**

**TatTrz1 (410) -DVTYDQRIQQFISKHP----DIKHIFCTQESCPNMITHPGAATIQTKLRRIDPERFPLLKFENTVKYPAPPE----------------GSSIELGRAGHKFQLMPRLVF**

**TviTrz1 (408) -NMTEDARIQQFIQQHT----DIKHIFCADEACPNMVTHPGAATIQTKLRRIDPERFPLLKFENTVRYPAPSE----------------GSPIELGRAGHKFQLMPRLVF**

**TreTrz1 (414) -NLSGDARIQQFIQEHA----TIKHIFCADDTCPNMITHPGAAAIQAKLRRIDPERFPLLKFENTVQYPAPSE----------------GSPIELGRAGHKFQFMPRLVY**

**AgoTrz1 (290) VTVSNELIRFMELFDG----KTVKHFVSHDTITPNNISFWDSSVTMLKLKTLQPSSYNIPFTDRTLSGDFYRCF-------DKQLPVDAVVDNTSESPFSSTIESSRVFI**

**CanTrz1 (293) VVFNDDIIELMETLN---H-GNVQHLISHSKISPNSLNFRGSALTTLKLKSMQPESYNLPKCDQILSKDFFDCF-------EKSTPLGTTLIQTSEGDIKSSIDKENVHI**

**SceTrz1 (292) VTINDNLFAFIDIFEKNNY-GKVNHMISHNKISPNTISFFGSALTTLKLKALQVNNYNLPKTDRVFSKDFYDRF-------DTPLSRGTSMCKSQEEPLNTIIEKDNIHI**

**ZroTrz1 (291) VTINDNLIKSMELFG-----NDVQMFVSHPKVCPNTIVFKGAALTVLKLKALMVQNFNLPQSNSILSKEFYECF-------SKPTDRGTTLSQSQEEPLRSTIPSENVHP**

**VpoTrz1 (295) ITIGKELIEFMEIFP-----DSIQHIVSHSLISPNTVTFKGSAITLLKLKSLQVDNYNLPRTDRIYSKEFFDCF-------NKNLPSDVSVIQQQEEPLTSTIDGKNVHV**

**LthTrz1 (292) VSIGQPLLSLMQQLDG----EKTQHFVSHFEVCPNGLVFQSSAVTTLKLKAVQNKNYNLPRTDRVFSKDFYECF-------QKELPQGSSRVQRNERPLSTTIDINKVHV**

**KlaTrz1 (295) VTITDDLFQFFDTFS---D-DQIQHYVSHSSISPNTIAYESAAIATLKLKALQVESYNIPITDRVYSREFFHCF-------PKDLPEGSSLVQQQEHVPSCLVKNENMHV**

**PpaTrz1 (297) CNLSRIHPILLNYFNAN---SHCQFVVSHKDITNNLINFTGASLVSLKLKALFPNFFQLPYSSLSNGTPFNEKFA-----------SNVHILNTRSCPVVLKPGSPDAVI**

**CalTrz1 (311) LDLD--LSDYKERFLSKFP-SDTYHVISHPSMTNNVIMNDRFISNTLKIKSILNENFNLVNSENFKPLS---NS-----------------------QVDRLHALQSYHL**

**CduTrz1 (311) LQLD--LLDYKKRFLSKFS-SDTYHVISHPSMTNNVIMNDRFISNTMKIKSILNENFNLVNSETFKPLS---DS-----------------------HVDRLHALQSYHI**

**CtrTrz1 (311) LDFD--LQHYKENFLSKFP-SDAYHIISHPSMTNNVILNDKLVANTIKLKSLMNENFNLVNSEEFQPLS---DG-----------------------HVERLHALQSYQI**

**CpaTrz1 (309) VKFD--MQDYQQSFLDKFP-ATTKHLISHKSITNNIIVNEKFASGHLKLKSILPDNFQLMNSDPFRPLSG--SD-----------------------KVGRLHALQCMGV**

**CguTrz1 (312) IDIG--SEAYRD-LLQKFP-SNCKHIVSHSKFDKDVLVFKSAAENLLKLKSLQSECYNLPYVADSNQSIPLEG-------------------------IFRLHQQQQVVV**

**PguTrz1 (312) IDIG--SEAYRD-LLQKFP-SNCKHIVSHSKFDKDVLVFKSAAENLLKLKSLQSECYNLPYVADSNQSIPLEG-------------------------IFRLHQQQQVVV**

**DhaTrz1 (313) IDFN--QPEYLD-FIKKFP-DNCKHVISHSKLSDDTLIFKTSAINVLKLKCLQKDHFNLPYIESYNPLQSDSAD-----------------------SIHKLQQLQQFHI**

**PstTrz1 (314) VDFE--TPAYQD-FIAKFP-RDSKHIISHPKLANNTLVFKRSAIDVLTLKCIMKDNFNLPHIEPYESLPEG--------------------------SVFKLHQLQQFHV**

**CluTrz1 (310) IDFR--SADYIS-FISQFP-QDCQHVISHSSIADDTLVFRTAAVHLLKLKCVLNNSFSLPYIEKHSPLDLSPN-------------------------THKLSSLQYFTI**

**YliTrz1 (368) MESYFASDPEKYFEWMESLGSNVMHIISAPWLDPHALTFRGSASLCYKLRNIVGDQFPLHGKDYRTLSDP--------------------KADFLADETIQNRFNMRLLT**

**AmaTrz1 (397) --LAETPTFQAFAQRFP---TSAKHFVFDPATLGHPVSFPSVAVSQTMLSTLVPTFFPGYVHTVHDEVQELPKG-----------LTEAVPTAQFAMPLLEIGIEPRFEL**

**BdeTrz1 (320) -------------------------VLTDQRYIDFMNSF--PSTTQNQLHMLDSSMFLLPYVAPPLKDLPVVFG--------------LPKSAKPAELLLSYQFEPKLLI**

**SpuTrz2 (354) --VLSDPRYVEWMNKFG---HQTRHFIISRRHNPMGISYLAAGNLQHKLNVLDAEVFPLPYFTNDPEEDLRTIMD-------------LPKGAVMATPLTNYIIEPAPKV**

**SpuTrz1 (398) VLENAAYRSWMNNFGET-----TQHIIISGKHCAKPIIFRGSATSQHRLNLLDPDVFRIPFYDNESISLNHEGLP---------------PRTMAANNLLIYQMEPHRKV**

**ScrTrz1 (349) VYNN-Q--KYQKWISSLST--KATNILSPTKPIETINYP-RSSSAITALNMLDDTSFPLGKNCYHDNLDTLKRDG-----------------YVIACPKMKFMFGKKPGI**

**SocTrz1 (348) VFNN-Q--RYKHWVSSLSN--KASNILSPTSELETVNYT-RSASAITALNMLDENTFPLGKNCYQESLDSLKRDG-----------------YVIACPKMKFMFGKKPGI**

**SpoTrz1 (365) VYKN-P--RYQSWISSFPS--EVSHLIASTEVNEVINYP-RSAVAIATLNLLDSKVFPLGFNCYEVKNVQK-NNR-----------------IAFAKPKLRFAFGKKTGI**

**SjaTrz1 (323) VLNH-P--SYLSWLNSFGD--NVSHLVSPLEPNHSVLFY-KSSLTSLALNLLHPTIFPLGTSFLQTKLCK--DGR-----------------FFKVVPRTTFTFGKTLKL**

**ScrTrz2 (300) VWGN-E--YAKFMKHPKLR--NSTHLISCIELAKDSLIFRRNRSANVLPSCRGFLSTHLS---------------------------------------SVHSLSENVFE**

**SocTrz2 (300) VWGN-D--YAKFLEHPKIQ--NIKHIISCVELGKDLPTFKRSKSVNVLPSCRGFLSDDLS---------------------------------------TTCNLPDNVLK**

**SpoTrz2 (294) IWGP-E--YIKFLSHPKFS--RAQHFISCIELASNNPVFQRNKGRNVLPACRDFAAFDIKP----------------------------------STLDTQTQLPENTYV**

**SjaTrz2 (284) LWSSEYVNNVMKKHKN------ITNIISAPELLYDRSLF--QRSRRFTSSLVCEQFYPWRSTKVRHARSILP------------------KDTYIAQQGLSVALRTD---**

**CneTrz1 (374) VPRSVWVDKRYQAWMQSFG-EKTKHLIANTTPATDHAVFNSAAWNTLHLSLIEPTIFHPPFLRHPSTSFTSLEST-------------LPSNVTFIHPNDFVKMYPPSPL**

**TmeTrz1 (348) IWMREDYQRWVQSFG-------TEPKVTDTTPSPNEIFFTSAAWNHLHLSQIDSTIFHFPNSLPQTTPPISLPSN---------TVLLQSGHSIRMHPPGSLELLPRPSK**

**AbiTrz1 (361) VLQDERYIDFMKGFPAT-----TEHVVSSSEYSPNPLTFTSHGFKQYCLSRLDPEVFPIPKFDISPPTSLASIPS---------------------LPIQTHFLSPKFII**

**CciTrz1 (457) VLED-P--RYIEFMNGFRD--GVDHLVTSKEHCPNRVTFTSATFNQLRLNKLDNEMFPIQKFNLEPSKSLSSIPN---------------------LPKNTQLMRPNVRI**

**ScoTrz1 (410) VLDD-P--RYVEFMNGFPE--QTHHIIASRDHCPDPITMTSAAFSQLRLNVLDPDIFHVPKFSLPSDTWLTDIPN---------------------LPPSTHVMTTDTQV**

**LbiTrz1 (397) VLGD-E--RYKAFMKEFAP--DVNHVVASREHCPDPVTFTSAAFNQLRLSELDNTMFPVPKYRLDPETKFSDIAG---------------------LPEKIHIMASNLYM**

**PosTrz1 (391) VLED-A--RYIEFMNGFSP--SVHHVVSSKDFTADPVTFTSVAFNQLRLNQLDQEMFPLPHFSLEARRTLSALPN-------------LPKQTIPLKASLFIKMRPFSPP**

**SlaTrz1 (424) VLED-E--GYKDFMDGFGS--DVQHIVASRKHGHDPVTFTSAAFNQLRLNQLDSEIFKIPKYRLEPDTDIRRGG-------------------YLYFLNIVCIRPPRPPL**

**HanTrz1 (403) VLED-P--RYIEFMNGFPD--HTHHLISSREHNNDPVTFTSCAFNQLRLNCLDPGMFPLIKYDLKYKKDLSSVPS---------------------LPANAAVMQIHTYV**

**PplTrz1 (430) VLED-E--RYKAFMRGFAD--GVHHLVSSREHGADNATFTTAAYSQLRLNQLDPDMFPPHHYSVLPRRDLSLVPG---------------------LPPLVELLQHNRLV**

**PhaTrz1 (415) VLED-E--RYKALLGKFAL--DVHHVVSSRQHCPDPVSFTTAAYNQLRLNRLDPEMFPTQKFSREPSKQLTDVPG---------------------LPQNTIPMYTDFYL**

**AbiTrz2 (359) VAFDGRYYGFLHGFSS-----HTKHFISCPELSRDYFTFPLNAWRLYQLTLLDVKTFPFLMRRVTKPQHTSCLVL---------------RSTELMRPGLQIRLDEDAFA**

**MglTrz1 (408) PLEVWQDERYQAWRRDFG--PACHHSVVNRDMCADTLTYTSNAISLLRLSRMDPDVFSVPGYRLEPRVRDPSTLP--------TQINTHIPLQPRGAPTQLPIIAPVFDK**

**MlaTrz1 (359) VLSD-E--RYVAWLSQFSG--HTKHYLSSPSHSPDLFSFEPSVLLQLKLSLVSPTLFPIPYRDLNPKIPVHIPT------------------LSHLDRFQVIPINGEFSP**

**PgrTrz1 (349) VVLD-E--RYIQWISKFAP--EVTHHLSTPSHSPDLFTFEASALLQLKLNLIAPSSFPIPHYSLTPALSCPSPE------------------LNLLSRYETFSLNSAKST**

**PxKxRN loop**

**AbeTrz1 (517) TASPTPP----------------------------------------SKPIKMPRRNSYSRVMGEILSQPGVQKRIEKLKASIPNGDAEI---------IALGTGSSCPS**

**TruTrz1 (515) TASSSPP----------------------------------------SKPINMSRRNSYSRVMGEILSQPGVQKRIEKLKASIPNGDAEI---------IALGTGSSCPS**

**MgyTrz1 (515) TTSSALP----------------------------------------PKPIKMSRRNSYSRVMGEILSQPGVQKRIEKLKESIPNGDAEI---------IALGTGSSCPS**

**McaTrz1 (516) ITTSVPKPLSA------------------------------------PKSGKAPRRNSYSRIMGEILTQPGVQKRVESLTKAIPNGDAEV---------IALGTGSSCPS**

**CimTrz1 (511) ENSQTERNIDIDQ---------------------------------IKRSVGDPVRGHVDAVRQKFN-EILFQTKVENMRQSIPNNDAEI---------ITLGTGSSLPS**

**CpoTrz1 (511) ENSQTERNIDINQ---------------------------------IKRSVGDTVRGHVDAVRQKFN-EILFQTKVENMRQSIPNNDAEI---------ITLGTGSSLPS**

**UreTrz1 (514) HSSQTEREIDIKQ---------------------------------IKESVSGLARGHVDAVRQKFN-DPSFQEKVEAFKQKIPDSDAEI---------ITLGTGSSLPS**

**AcaTrz1 (557) NSDGVHKPLNTNN---------------------------------ITKQISSRVLKLAREAKEDIN-SPEFQDLLKKVRQNIPNQDAEI---------ITLGTGSSLPS**

**AdeTrz1 (563) NSSGVHQPLDGEG---------------------------------IRNNVSFRVKRAARAANQDIQ-SPQFQEFLENVRRNVPNQDAEI---------ITLGTGSSLPS**

**PbrTrz1 (543) KRDNVQKPLNADV---------------------------------IKNDIPPRVIETAHQAEHEIE-GHEFQQILADVRRNLPGQDAEI---------ITLGTGSSVPS**

**AclTrz1 (526) SRSEIVPLFNPAE---------------------------------TMQRIPRSIEQRVTAIRRRVQ-KPEFQKKLAEFRQGLPGANVEI---------VTLGTGSSSPS**

**AfuTrz1 (519) NRSEVVPLFNPAE---------------------------------TVQRIPRSIEQRVTAIRRRVM-NPQFQEKLAEFRKDLPGANVEI---------VTLGTGSSSPS**

**NfiTrz1 (519) NRSEVVPLFNPAE---------------------------------TVQKIPRSIEQRVTAIRRRVM-NPQFQEKLAEFRKDLPGANVEI---------VTLGTGSSSPS**

**AcrTrz1 (524) NYSAVEPHFDTAK---------------------------------VDHKMPRAIEQRMSTIRKRVK-KPEFTQKLESFRKNLPGSDVEI---------ITLGTGSSAPS**

**AspTrz1 (525) NYSEVEPHFEPAQ---------------------------------VQRQMPWAIEQRLSTIRKRVK-KEAFTKKLESFQKDLPGANVEI---------ITLGTGSSAPS**

**AflTrz1 (531) NRSEVVPRFNAIE---------------------------------AVQRMPVAVQKRMNTIDRRVK-KEEFEEKLRQFRKDLPGADAEI---------ITLGTGSSSPS**

**AorTrz1 (531) NRSEVVPRFNAIE---------------------------------AVQRMPVAVQKRMNTIDRRVK-KEEFEEKLRQFRKDLPGADAEI---------ITLGTGSSSPS**

**AteTrz1 (525) NRSEVMPLFNTSK---------------------------------IANKMPRAVDQRMRTIQKRIK-KPEFQQKLEDYRKGLPGADVEI---------IALGTGSSSPS**

**PchTrz1 (533) NSDEVMSRFNPAT---------------------------------ALHSIPRSVEQRMAVIRKRVA-KPDFQRKLQAQRREWPGADAEI---------IALGTGSSVPS**

**AniTrz1 (512) NSDEVPRRLNAGS---------------------------------ILTKMPQSAVRRARLVTKRLQ-HPLRQEKIQQFVSDIPGADAEI---------ITLGTGSSAPS**

**PmaTrz1 (499) NESELIESLETRD---------------------------------VVTRLPKAVQQRVNVIRKSLT-KHEVISQFMKLRDGLPPLAQEAEV-------VTLGTGSSSPS**

**TstTrz1 (503) NESELIPQLDTRD---------------------------------VVTRIPKAVQQRLNVIRRRLT-NHEVVRQVMDLRKELPPLAMESEV-------VTLGTGSSSPS**

**AbrTrz1 (503) KKDTASKLFTPNE---------------------------------IQSETPSEVLELAKQAQATVQ---ENRGNMQMWKLLLARPDTEV---------TTLGTGSALPS**

**CheTrz1 (503) KKELISRPFDDEA---------------------------------ARRDTAPEVLELAQEAQTVLQ---HHREDMNKWRQLLARPDTEV---------ITLGTGSALPS**

**PtrTrz1 (503) HKETATPLFDLET---------------------------------ARKEASPEVLELAQKAQESLR---QHRGDMLKWRQLLARPDTEI---------TTLGTGSALPS**

**PnoTrz1 (503) KEETKTQMFNSNV---------------------------------VKHETDAEVLRLAAEAQQAVK---DDQINQDAWKQLLARPDTEV---------ITLGTGSALPS**

**MfiTrz1 (529) QSKEVVPTLDVAQ---------------------------------IVSEVPQDVMDEARKAQEATK---TTPEDVQKWLGKLPPDAKDAEV-------ITLGTGSALPS**

**MycTrz1 (513) QTKTEVAPLNIAE---------------------------------TISETPQDVLAEAAKSQEAAK---VVSDELRTWIDSLPPGAADAEV-------ITLGTGSALPS**

**SscTrz1 (489) QDDKCIPYLDTQQ---------------------------------VVKEADPEVLALANEARKEIMS-PEYQAKLDEVQKDIPCKDAEV---------ITLGTGSALPS**

**CglTrz1 (593) LTDLVVPPMDTKR------------------------------PLWELATYTPQVMKLARVAQKAVS-ETDFLAEVERLQQDLPSPQTEI---------VPLGTGSAMPS**

**MthTrz1 (444) LTDAVVPPMDTKK------------------------------PLWELGSYSPQVPGLADAARKAIS-DPAFGAEVDRSQQDLPSPQTEI---------IPLGTGSAMPS**

**TteTrz1 (484) ITDGVVPVMDTKR------------------------------PIWELMTYAPQALERASAARNAIA-APSFLTEVEKSQHDLPSPETEI---------VPLGTGSAMPS**

**PanTrz1 (450) SSEFLVSPIDPIG------------------------------TLKALVGQHREVVEMAREASKKIA-DPAFLVEVEESQKDIPNRDTEI---------IPLGTGSALPS**

**NcrTrz1 (695) QRQFANAVMDTIK------------------------------PIKELTSTHAKAFNLALEAQTKVS-DPAFAARVAESEQDIPNRDAEI---------VTLGTGSALPS**

**NteTrz1 (695) QRQFANAVMDTIK------------------------------PIKELTSTHAKAFNLALEAQTKVS-DPAFAARVAESEQDIPNRDAEI---------VTLGTGSALPS**

**NdiTrz1 (695) QPNFANAIMDTIK------------------------------PIKELTSTHAKAFNLALEAQAKIS-DPAFAARVAQSEQDIPNRDAEI---------VTLGTGSALPS**

**SmaTrz1 (710) QPKFAIDIMDTIK------------------------------PIKELTSTHAKAFNLALEAQEKIA-HPAFAARVAESEQDIPNRDTEI---------VTLGTGSALPS**

**MgrTrz1 (570) QDDRAQPLLDTN---------------------------------AKELQPTSAVKQLVATARETVE-KQEFQLLVQADNKNLPCPNTEV---------IALGTGSSLPS**

**FgrTrz1 (491) DVGNVVPFPDLV-------------------------------QQFKSINEDVEIMKLVEEASREIS-DPEFLRKLEEDEKDIPNRDTEI---------IPLGTGSSIPG**

**FoxTrz1 (490) GEGEIAPFPALG---------------------------------EAAQSVSDEILELARKAREETS-DPEFLRKLEEDEQDIPSRDAEI---------IPLGTGSSIPG**

**FveTrz1 (493) GEGEIAPFPDLM---------------------------------EAAQSVSDEILELARKAREETS-DPEFLRKLEEDEQDIPNRDAEI---------IPLGTGSSIPG**

**NhaTrz1 (492) DTGTVAPFPNLA---------------------------------EAAQSVSDEIMDLARKAQEETS-DPEFLKRIEEEEKDIPNRDAEI---------IPLGTGSSIPG**

**TatTrz1 (499) DDQAIAPFPDLV---------------------------------EAHESVDEEVLKMAEAAHAEAT-DPAFVARIEEQELDIPNRDAEI---------IPLGTGSSVPS**

**TviTrz1 (497) DDQAIAPFPDLV---------------------------------EAYESVDDEVLKMAEEARSEAT-DPKFLERVEKEEVDIPNRDAEI---------IPLGTGSSVPS**

**TreTrz1 (503) DDQAIAPFPDLV---------------------------------EAYESVDDEVLKMAEEARSEAT-DPKFVERIEQEEVDIPNRDAEI---------IPLGTGSSVPS**

**AgoTrz1 (389) YSTRVTASIEPYTPGD---------EPLKINI-----TESPDRYQSWEEVYQKRIEPLKIE-NSNLHDVVTSQKEVDNFNTEGKRRDVEI---------MTLGTGSALPS**

**CanTrz1 (392) FKRGTDVTLEPYTKNSEL---EVQDLNSKVIT-----TELPSTADFWKRAFHNHVVPLQIPGATYENIVENQFHVNNFNSSPEKATQPEI---------ITLGTGSALPS**

**SceTrz1 (394) FSQNKTVTFEPFRMNE---------EPMKCNI------NGEVADFSWQEIFEEHVKPLEFPLADVDTVINNQLHVDNFNNSAEKKKHVEI---------ITLGTGSALPS**

**ZroTrz1 (389) FTQGSVTQIESFTKGE---------ESMRIKI------ENNCPDWSWDFAYRRHVKPLNLPSTSFEKIVTEQQG-VDNFDTPEKKSNVEA---------ITLGTGSALPS**

**VpoTrz1 (393) YRQNTILKLDPFTKDS---------NDLRYKI-----TNKNSDIVDWKKYYDQFLKPFSDEIGSVDDVVNSQINTNNYNNTIEKSRRVEI---------VTLGTGSALPS**

**LthTrz1 (391) LLQNDGIIVEPYTDGE---------NEIKVRH-----SKSSKKTKTWAELYEQHVTPLGIPGATYNSTVESQKNVNNFN-SEAKKGKIEV---------VTLGTGSSLPS**

**KlaTrz1 (394) FEKMKGIALEPFTVGK---------ENMVIHE---SQLPTAKQSSTIDELYESHVVPLCIEGSSLERTVHEQVHVNNFD-NKTKLNEIEI---------ISLGTGSALPS**

**PpaTrz1 (393) VENSLESVNQFWKAR--------------------------------FDESLLDTFGISFDSIEPYINDNKLVTSGSLSDSETLKDKVQV---------VTLGTGSSLPS**

**CalTrz1 (392) NDNGVVPDSSSAIKT------------------------------TNKSLFEEEVESLDIPNAANFETMEKTKIELDCNKHTNLKDRVQV---------FMLGTGSALPA**

**CduTrz1 (392) NDNGVVPDSSAAIKT------------------------------TNKSLFDEEVGSLDIPNAADFESMEKTKIELDCNRHNNLKDRVQV---------FMLGTGSALPA**

**CtrTrz1 (392) TENEVHADNSCSVQA------------------------------TNKSLYDNEVAPMEIPEAVDYESMTKTSIELDDETTSSLKDKVQI---------CMLGTGSALPS**

**CpaTrz1 (391) DDTGVHGDNTNVLRS-----------------------------------SNLSLYDEMTHSSDIAPPFETLQTRFNLLKDSAADLKDLVHV-------STLGTGSALPS**

**CguTrz1 (393) KPDEITNNYRQDSNE------------------------------TWESIYDSVSTQGSFRLPEKSQLLSSEPIPFNSS-NTSLRDKVQV---------YTLGTGSALPS**

**PguTrz1 (393) KPDEITNNYRQDSNE------------------------------TWESIYDSVSTQGSFRLPEKSQLLSSEPIPFNSS-NTSLRDKVQV---------YTLGTGSALPS**

**DhaTrz1 (396) ETSSIKSDDSLISED------------------------------TWSSLYDSNILPLDIKNINKTDILESEPISLAQI-PGSMKDQVQV---------VTLGTGSALPS**

**PstTrz1 (394) QSSGVEVDTSLIQSD------------------------------SWESIYNENIAGLNLPDVNLGDIVDSEPSTLDAL-SGSLKDQVHI---------VTLGTGSALPS**

**CluTrz1 (391) DPSGVSLDEQNIISE----------------------------TWESLYDAEIPSSEVLAGTDKSTILQNGILPLSPIPNASSLKDHVQV---------VTLGTGSALPS**

**YliTrz1 (458) QGDAVSIENTTN---------------------------------------TSRVVNSGLREYEGLGYTPEETSVDRPLRNDDGTLKCKAEL-------LTLGTGSACPS**

**AmaTrz1 (491) IKSDRAAVSAVAG------------------------ANEMLGNDKKLRSLLSAKTAAAESIKTESGATDADMAEATAETAVTATNGTENNDEGGDWIVMPLGTGSAIPS**

**BdeTrz1 (389) DVSECRSELVMETG--------------------------TNAYKEYVCLAKQTVDAINAAEKERSDAMAVSRKDLPLLSVPDPTEGVAV---------TPLGTGSAIPG**

**SpuTrz2 (446) DNSEALRP------------------------------------------FLREEVSGLAEPDLISCIKKDIERRHATSSVVPPEKDVTI---------IPLGTGAAIPG**

**SpuTrz1 (488) DVSEQKTPWDHKDP------------------------------NGKVLATLDKPRLLEYQVEAKRLREEILASAAEAENTRKPGDDVLV---------CTLGTGAALPS**

**ScrTrz1 (436) EYPEPGLD---------------------------------------VSQLRQNILEEKPDYKHLLT-KAQLLNSSLNLPKDFAGSDIQF---------STLGTGSAMPS**

**SocTrz1 (435) EYPEAGMD---------------------------------------VSQLRNNISEEKPDYKELFN-KSRLLNESLSLPNDFAGSEIEF---------CTLGTGSAMPS**

**SpoTrz1 (451) DDSEVGVS---------------------------------------IEELKDKILKEKPDYKSFVEEAQKYVSDKPKA-PSFAGSDIQI---------CTLGTGSAMPS**

**SjaTrz1 (408) NEPEAYPS----------------------------------------IDSLLDTLKIENPDYVRLAGKARELVCQKTTSMIEPGHDIQL---------ITLGTGSAMPS**

**ScrTrz2 (366) LEEGLS---------------------------------------------VEISDQKCFVKNENTENVGVKTHSFNELPSPSVGYVV-----------DILGTSATSPT**

**SocTrz2 (366) LEEGLS---------------------------------------------LELSNQECLVKSENKENTKVEPRLSNSLPESSKGYVV-----------DILGTSATSPT**

**SpoTrz2 (365) LKEETS----------------------------------------------MVLYDEQCKISESPSYSPVKLAKKFSSFNPLPFENEGYTL-------DVLGTSATCPT**

**SjaTrz2 (365) --------------------------------------------------------GCSVSGHKESFDESETRNSMKSREIITSHSTITL---------DTLGTGSSCAS**

**CneTrz1 (470) ETFPWHEKDLPFTVTEN----------------------QADDAREKVKKEKKEYGEVCEKARNTVRQLDMEKAKAKEMNNENEYSDDIVV--------TTLGTGSAIPS**

**TmeTrz1 (442) DIPFPLPYLSSISDIPSPSSE-----------YLNACLKAQETVKHLSLPIPPKTSATETTPLQSVNPILPLQDVGMKDQHDKVTYEAEPKVGDDII-ITTLGTGSALPS**

**AbiTrz1 (445) PVRPAGPPKLDPALQVD----------------------------LFHPAFDQPQPLPDKTLRAFGMAKEAVASFVGPQDPQLSGVKI-----------LPLGTGSSHPT**

**CciTrz1 (541) GVRPYAPPKLDPQTEG----------------KDLFHPLVEGKKLGEGESIEVQLPEETVEKFEQAREAVKKIAEGAAKPVERPGDDVVV---------VPLGTGSAVPG**

**ScoTrz1 (494) HIRPPSPPKRDPYAQ----------------------------DKFHPVCQAPVELTQATREAFDKAQAVVSQRIAEGAVPKLPGADVTV---------LPLGTSSAVPS**

**LbiTrz1 (481) NMRPPAPPIIENLPGG-------------------------GDRFHPAVKKSSSLDLSQLTSGKFAAARLNVANALENGMDVPKGAEVGV---------LPLGTGSALPS**

**PosTrz1 (483) TVDAIYSSQDLPT---------------------------HDLFHPAVTAQTSTNVPLPLPPSTLEKFEVARSLVGDSIGHVTPGSGTEKANDVIV---TPLGTGSALPT**

**SlaTrz1 (510) HEKDTEH---------------------------------YDLFHPATSSSAPFSLPELTMKRFAEAKSAIEDRIANNTVPKQPGDDITI---------LPLGTGSAVPS**

**HanTrz1 (487) DMRPPSKPVLESFFVE-------------------------RDSFHPAVATPRPISLLASTRDKFTRAQSAVQAGLATRNSDEPSPGDDLVV-------IPLGTSSTVTT**

**PplTrz1 (514) DVRPPRKPCHDVLAQQ------------------------YDHFHPAITGSQGVLLPAAVADVFRKSRAKVKQLAAARDTSKKPGDDVEI---------IPLGTSSALPS**

**PhaTrz1 (499) QVRPIGEPRLGRHASG--------------------------IDTFQPIVNSSEPLELPVPTQEAFEAARERGIKALEGRGSKPGDDVMI---------IPLGTGSAIPT**

**AbiTrz2 (449) TGSIVYNFGSPAQ----------------------------------TLSSILQIAPLPKQVQAAFSSARRNIFRHPDFQRSEKRISAKV---------VCLGTGKSIPN**

**MglTrz1 (508) PLHEMHQHATLDDDDNDS-----------------------------KNSASPGSKATWDAYCAMAAQVRQSASVPPRTPEAGLADQVEL---------TTLGTGSSAPS**

**MlaTrz1 (446) GPELVRLDYDLIN---------------------------------NPKSTSELIKRLGLEEGFPNILARIRQTEMENLDPKPDNIAGRIMV-------TTLGTGSANPS**

**PgrTrz1 (436) SDNNMQIDYSVFS-------------------------------CSDRSELMSKLGVKPGLADELKYVKSDNDRSVVPTGEAEFADGIIV---------TTLGTGSAAPS**

**motif I motif II**

**AbeTrz1 (578) RYRNVSGTLLRVPG--------------HGNYLFDAGEGTLGQLKRTFGPDE----------------------LKEVLRDLRVIWISHLHADHHLGTVSVIKAWHEAVY**

**TruTrz1 (576) RYRNVSGTLLRVPG--------------HGNYLFDAGEGTLGQLKRTFGPDE----------------------LKEVLRDLRVIWISHLHADHHLGTVSVIKAWHEAVY**

**MgyTrz1 (576) RYRNVSGTFLRVPG--------------HGNYLFDAGEGTLGQLKRTFGPEE----------------------LKDVLRDLKVIWISHLHADHHLGTVSVIKAWHEAVY**

**McaTrz1 (581) RYRNVSGTLLRVPG--------------HGNYLFDAGEGTLGQLKRIFGPEE----------------------LKAVLRELKVIWISHLHADHHLGTVSVVKAWHEAVH**

**CimTrz1 (578) MYRNVAGTLLRVPG--------------HGSYLFDCGEGTLGQLKRVFSPEE----------------------LKEVLRELKVIWISHLHADHHLGTVSVIKAWYEETF**

**CpoTrz1 (578) MYRNVAGTLLRVPG--------------HGSYLFDCGEGTLGQLKRVFSPEE----------------------LKEVLRELKVIWISHLHADHHLGTVSVIKAWYEETF**

**UreTrz1 (581) MYRNVSATLLRVPG--------------SGSYLFDCGEGTLGQLQRVFSPEE----------------------LKEVLRELKVIWISHLHADHHLGTVSIIKAWYEEVF**

**AcaTrz1 (624) KYRTVSATLLRVPG--------------VGNYLFDCGENTLGQLQRTFAPEE----------------------LQEVLRDLKVIWISHLHADHHLGTVSVIRAWHKVTH**

**AdeTrz1 (630) KYRTVSATLLRVPG--------------VGNYLFDCGENTLGQLQRAFSPEE----------------------LREVLRDLKVIWISHLHADHHLGTVSVIRAWHEVTH**

**PbrTrz1 (610) KYRSVSGTLLTVPS--------------VGNYLFDCGENTLGQLQRVFSPQE----------------------LRKVLQDLKVIWISHLHADHHLGTVSVIRAWYKVVY**

**AclTrz1 (593) KYRNVSSTLVNVPG--------------VGYYLLDCGENTLGQLKRMYDPKK----------------------LREVLQNLRMIWISHLHADHHLGTASVIKAWFRENY**

**AfuTrz1 (586) KYRNVSSTLVNVPG--------------VGSYLLDCGENTIGQLKRMYEPKK----------------------LQEVLQNLRLIWISHLHADHHLGTASVIKEWFRANY**

**NfiTrz1 (586) KYRNVSSTLVNVPG--------------VGSYLLDCGENTIGQLKRMYEPKK----------------------LQEVLQNLRLIWISHLHADHHLGTASVIKEWFRANY**

**AcrTrz1 (591) KYRNVSATLLNVPG--------------YGYYLLDCGENTLGQLKRVFEPEK----------------------LREVLQNLRMIWISHLHADHHLGTASVIRAWFQENY**

**AspTrz1 (592) KYRNVSATLLNVPG--------------YGYYLLDCGENTLGQLKRVFSPEK----------------------LREVLQNLRMIWISHLHADHHLGTASVIKAWFQENY**

**AflTrz1 (598) KYRNVSSTLVHVPG--------------YGYYLLDCGENTLGQLKRVFEPEK----------------------LREVLQNLRMIWISHLHADHHLGTASVIKAWFQENY**

**AorTrz1 (598) KYRNVSSTLVHVPG--------------YGYYLLDCGENTLGQLKRVFEPEK----------------------LREVLQNLRMIWISHLHADHHLGTASVIKAWFQENY**

**AteTrz1 (592) KYRNVSSTLLHVPG--------------YGYYLLDCGENTLGQLKRVFEPEQ----------------------LREVLQNLRMIWISHLHADHHLGTASLIRAWFHENY**

**PchTrz1 (600) KYRNVSATLLKVPG--------------YGYYLLDCGENTLGQLKRVFEPEE----------------------LREVLQNLRMIWISHLHADHHLGTVSVIKAWHAENF**

**AniTrz1 (579) KYRNVSSTLVHVPG--------------RGYYLFDCGEGTLGQLKRMFSPEQ----------------------LREVLQNLRLIWISHLHADHHLGTVSVIKAWYQENY**

**PmaTrz1 (568) KHRNVSATLLNVPG--------------KGYYLLDCGENTLGQLKRTYPPEQ----------------------FREVMQNLRMIWISHMHADHHLGSVAVIREWYEVNY**

**TstTrz1 (572) KHRNVSATLLKVVG--------------KGYYLLDCGENTLGQLKRTYPPEQ----------------------FREVMQNLRMIWISHMHADHHLGSVAVIKEWYEVNY**

**AbrTrz1 (568) KYRNVSATLVRVPG--------------IGNYLLDCGENTLGQLSRVFPHEE----------------------LIDIIKNLRLIWISHLHADHHLGTAAVIRAWYQIVH**

**CheTrz1 (568) KYRNVSATLVRVPG--------------IGNYLFDCGENTLGQLARVFPHEE----------------------LIDVLKNLRVIWISHLHADHHLGTAAVIRAWYQVVH**

**PtrTrz1 (568) KYRNVSATLVRVPG--------------VGNYLLDCGENTLGQLSRVFSHEE----------------------FVDVLKNLRMIWISHLHADHHLGTAAVIKAWYQLVH**

**PnoTrz1 (568) KYRNVSATLVRVPG--------------VGNYLLDCGENTLGQLSRVFPPEE----------------------LRDIIKNLRLIWISHLHADHHLGTTGVIRAWYHLVH**

**MfiTrz1 (596) KYRNVSSTLVRVPG--------------WGNILLDCGENTIGQLQRVFPAED----------------------FKQVLRELRLLCISHMHADHQLGTTSVIKAWYQEVH**

**MycTrz1 (580) KYRNVSATLVTVPG--------------WGNILLDCGENTLGQLRRVYGEQA----------------------LKQIWQDLKIIVISHMHADHHLGTASVIKAWYEQVH**

**SscTrz1 (556) KYRNVSATLLRIPE--------------YGNYLFDAGENTLGQMKRVFGKD-----------------------LPGVLSNLKAIWISHLHADHHLGTASVIKAWHDETS**

**CglTrz1 (663) KYRNVSATLIRVPG--------------WGNYILDCGENTLGQLRRAFGYQA----------------------ADDILRDLRAIYISHAHADHHLGTVNVLSRWREVAP**

**MthTrz1 (514) KYRNVSATLIRVPG--------------WGSYLLDCGENTLGQLRRALGHQG----------------------ADEVLRDLRAIYISHVHADHHLGTVSVIARWREVVP**

**TteTrz1 (554) KYRNVSATLIRVPG--------------WGSYILDCGENTLGQLRRCFGYEG----------------------ADEVLKDLRAIYISHLHADHHLGTVSVIARWRQLSL**

**PanTrz1 (520) KYRNVSATLIRVPG--------------HGSYLFDCGENTLGQLRRLYGNED----------------------TDEVLKDLRAIYISHLHADHHFGTASMMARWNKVTA**

**NcrTrz1 (765) KYRNVSATLIRVPG--------------YGSYLFDCGENTLGQLRRVYGYAE----------------------TDAILRDLRAIYISHLHADHHLGVPSVLARRAAANA**

**NteTrz1 (765) KYRNVSATLIRVPG--------------YGSYLFDCGENTLGQLRRVYGYAE----------------------TDAILRDLRAIYISHLHADHHLGVPSVLARRAAANA**

**NdiTrz1 (765) KYRNVSATLIRVPG--------------YGNYLFDCGENTLGQLRRVYGYTE----------------------TDAILRDLRAIYISHLHADHHLGVPSILARRAAANA**

**SmaTrz1 (780) KYRNVSATLIRVPG--------------YGSYLFDCGENTLGQLRRVYGYAE----------------------TDVILRDLRAIYISHLHADHHLGVPSVLAQRAAVSA**

**MgrTrz1 (637) VYRNVSATLLRVPG--------------AGNYLFDCGEGTLGQLRRHYGIEE----------------------ADEILRDLRVVWLSHPHADHHLGTARLLQAWDAATS**

**FgrTrz1 (560) KYRNVSSTLIRVPG--------------IGNYLLDAGEGTLGQIRRLFGEKE----------------------TGNILRDLKCIVISHLHADHHLGTPMLIKAWYEHTI**

**FoxTrz1 (557) KYRNVSSTLIRVPG--------------IGNYLLDVGEGTLGQIRRLFGEEE----------------------TGNILRDLRCIVISHLHADHHLGTPNLIKAWYEHTI**

**FveTrz1 (560) KYRNVSSTLIRVPG--------------IGNYLLDVGEGTLGQIRRLFGEEE----------------------TGNILRDLRCIVISHLHADHHLGTPNMIKAWYEHTI**

**NhaTrz1 (559) KYRNVSATLIRVPG--------------IGNYLLDCGEGTLGQIRRFFGDEE----------------------TGNILRDMKCIVVSHLHADHHLGVPSFVKAWYEHTL**

**TatTrz1 (566) KYRNVSATLIRVPG--------------IGNYLLDCGEGTLGQIRRLFDAEG----------------------TADILRNMRCIVISHVHADHHMGTPSLIKAWYEQTL**

**TviTrz1 (564) KYRNVSATLVRVPG--------------IGNYLLDCGEGTLGQIRRLFDAEE----------------------AADILRNLRCIVISHVHADHHMGTPSLIKAWYEQTL**

**TreTrz1 (570) KYRNVSATLIRVPG--------------IGNYLFDCGEGTLGQIKRLFGAEE----------------------AADVLRNMRCIVISHVHADHHMGTVSLIKAWYEQTL**

**AgoTrz1 (475) KYRNVISTLLKIPYDA-------DGRILNRNIILDAGENTLGSIHRLLHKVK----------------------IPRFFRDLKMIYLSHLHADHHLGIASLLKEWYLHNK**

**CanTrz1 (485) KYRNVISTLVKVPFNN-------NGVISNRNVILDAGENTIGMLKRMFNSTE----------------------LLSIFKDLKLIYLSHLHADHHLGIISILREWNRYTK**

**SceTrz1 (480) KYRNVVSTLVKVPFTDA------DGNTINRNIMLDAGENTLGTIHRMFSQLA----------------------VKSIFQDLKMIYLSHLHADHHLGIISVLNEWYKYNK**

**ZroTrz1 (474) KYRNVLSTLLKIPYKTE------TGNIDSRIILLDAGENTLGTIRRHFPSTT----------------------VCKIFKNMKMMYLSHLHADHHLGIISILKEWFKYHQ**

**VpoTrz1 (480) KYRNVVSTLIKVPYINK------DNSIENRCVLLDAGENTLGTILRMFSSID----------------------CKKLFKDLKMIYLSHLHADHHLGIISILEEWYKYNQ**

**LthTrz1 (477) KYRNVISTLVKIPYNN-------KGKIDNRNILLDAGENTLGAILRLIPSVD----------------------IPGFFRDLKLIYLSHLHADHHLGIASILNEWHKQNL**

**KlaTrz1 (482) KYRNVISTLVKIPYTEV------DGTVVNRSILLDAGENTLGTIHRTIPDID----------------------VPKLFRDVKLIYLSHLHADHHLGIASLIKEWYKHNK**

**PpaTrz1 (462) KYRNVISTVLRVPYTQDG-------KLQYRSIIMDGGENTIGSIKRIYGPD-----------------------YPKFFKELQCIYLSHLHADHHLGIISLIEERFKHNN**

**CalTrz1 (463) ICRNVLGNLIRLPYQDDS-----G-AISYRSIVLDGGENTIGSLLRLFGHENGKE-------------------FVQIFQELSLIHLSHLHADHHLGIVSLINEWFNINQ**

**CduTrz1 (463) ICRNVLGNLIRVPYQDGS-----G-TISYRSIILDGGENTIGSLLRLFGHENGKE-------------------FFQIFQELSLIHLSHLHADHHLGIVSLINEWFNINQ**

**CtrTrz1 (463) ISRNVLGNLLRIPFQHDD-----G-SISYRSIILDGGENTIGSLLRTFGHDNYKY-------------------VNQIFQELSLIHLSHLHADHHLGIVSLIGEWFKLN-**

**CpaTrz1 (459) ISRNVLSNLIRIPYQHRD-----G-SITYRSIILDAGENTIGSMLRNFGHKDESD-------------------LKQIYSELTLIHLSHLHADHHLGMISIINKWFELNS**

**CguTrz1 (463) IPRNVISTLVRIPYSSGD-----K--VEFRTMILDGGENTLGTINRMFRHGDGSK-------------------LQQIFSELSLIHLSHLHADHHLGLVSIINEWFKHNK**

**PguTrz1 (463) IPRNVISTLVRIPYSSGD-----K--VEFRTMILDGGENTLGTINRMFRHGDGSK-------------------LQQIFSELSLIHLSHLHADHHLGLVSIINEWFKHNK**

**DhaTrz1 (466) IHRNVISTLVRIPYIDNN-----T--VKFRTIMLDGGENTLGTMMRNFGHNNKEQ-------------------LIKIFEELSLIHLSHLHADHHLGIISIINKWFEINI**

**PstTrz1 (464) LHRNVISTLVRIPYHSSQ-----G-DIRFNSILLDGGENTLGTFSRNFGHNMGEQ-------------------ARKVMKELSLIYLSHLHADHHLGIVSVINEWFLVNN**

**CluTrz1 (464) IHRNVLSNLVRIPYRDEE-----TQEIRFRAILLDGGENTIGTLMRNFGHDSSKQ-------------------LKQIFSELRLIYLSHLHADHHLGIISVISAWAEANK**

**YliTrz1 (522) KYRNVAGLIMRVPRADG----------SFTGVVMDCGEGTYGTLTRMYSPEA----------------------CLQIMREIKMIYISHLHADHHLGTPTFIEQWLKANP**

**AmaTrz1 (577) QFRNVSSTLLSTPR---------------GPVLFDCGEGTLGQLSRLFPPAGVVDSATPLTGTTGATLRQRHPTLEALLGDLKLLFVSHLHADHHLGLTNILKHTTHPIT**

**BdeTrz1 (464) KYRNVSSTLIQTPQ---------------NAYLLDAGEGTYGQLFRHFGPIE----------------------LQRVFESLSFMCVSHMHADHHLGTITVLLEWAKATE**

**SpuTrz2 (505) KYRNVSSTFLMLSS---------------GNVLLDAGEGTLGQLFRHFGPDM----------------------MKEALRNLRMIFVSHLHADHHLGVVRILKYWDEVRD**

**SpuTrz1 (559) RYRNVSSTLVYTPS--------------HGSILFDAGEGTYGQLYRRFKGADGPS-------------------LEDVLTNLKFLFISHMHADHHLGAFKVLLERKKLFD**

**ScrTrz1 (497) LYRNVSSTYVKIPK--VRDSESVEASSYTNNILLDCGEGTLGRLARQYGDC-----------------------LNDEISSLKWIYISHMHADHHAGVIGVLRLWFSISN**

**SocTrz1 (496) LYRNVSSNFIKIPKRATEREDFIESKRLTNNILLDCGEGTLGRLARQYGDS-----------------------LNDEISLLRWIYISHMHADHHAGVIGVLKLWFSIST**

**SpoTrz1 (512) LYRNVSSTYVRIPVDKKCMEDSAIS---MKNILLDCGEGTLGRLSRQYGDN-----------------------LKYEIASLRWIYISHMHADHHAGVIGVLKAWTKYSD**

**SjaTrz1 (469) LYRNVSANLLRIPI-------FGETN-KTYNILLDCGEGTLGRMARQFGDR-----------------------LTEELAALRWIYISHMHADHHAGVNGILQAWAKVTE**

**ScrTrz2 (420) LYRSLSCYLVNVDN---------------CFILLDCGEGSYSQLIRQYGTN-----------------------LDSVLKQLRLIFISHMHADHWLGLVNILLAWNESTR**

**SocTrz2 (420) MYRSLSCYLVNVDN---------------NIVLLDCGEGSYSQLIRQYGTN-----------------------LDSVWRQLRLIFVSHMHADHWLGLVNILLTWNNSTR**

**SpoTrz2 (422) WRRSLSSYSVAIDG---------------TVIMLDCGEGAISQFFRQYGTN-----------------------TEPMLRKLKAIFITHLHSDHYLGLLNVLQAWNKANT**

**SjaTrz2 (410) IVRNVSGYFLSSGK---------------DTFLLDCGEGSLSQLSRQYGSN-----------------------TDVVLSRLKCIFISHSHADHHYGLPSIIQALLSFHL**

**CneTrz1 (550) KYRNVSSTHLAIP--------------SLGGILLDAGEGTLGQLRRRFGDG-----------------------LKSVLGEMKMVFVSHMHADHHLGVNAVLEERFRLGI**

**TmeTrz1 (540) KYRNVSSTLVEIP--------------GTGGVLLDCGEGTLGQLRRRFGD------------------------LSELWKDLKMIFISHLHADHHLGLQSILEDRFKHNI**

**AbiTrz1 (516) KYRNVLSTLIRTP---------------SGNILLDCGEGTTGQLTRYFGKGEEG--------------------IDDILRNLKCIFVSHAHADHHMGLAKLLRQRRLLSK**

**CciTrz1 (626) RYRTVSSTLIQIPN--------------WGNILLDAGEGTYYQLARHFGEEG----------------------VKEVLRDLKCLYVSHVHGDHHMGVPMILRKRKEASP**

**ScoTrz1 (567) KYRNVSSTLIRIPG--------------HGSILLDCGEGTWGQLAREYGTDESAPNN-----------------VYDVLRDIKCIFVSHLHADHHGGLSTLLAKRRQLDP**

**LbiTrz1 (557) KYRNVSSTLIQIPG--------------WGNILLDAGESTWGQLVRYFGTDDTSSPN-----------------VWDVLRNLKCVFISHIHADHHLGLSMILTKRRSLNP**

**PosTrz1 (563) KYRNVSSTLIRIPK--------------WGSILLDAGEGSWGQLIRLFGAPNPERAGQDVEN------------VETFLRDLRCLFVSHIHGDHHMGVAKILAKRRQLDP**

**SlaTrz1 (578) KYRNVSGTLIHIPN--------------YGNILLDAGEGTWGQLARHFGISDTPYN------------------VSQALRDLKCIFISHIHGDHHIGLAKILAMRQSLSP**

**HanTrz1 (565) RYRNVSGILLKVPE--------------RGYVLFDAGEGTFGQLMRTFGSDATQPEN-----------------AWQVLRELKCIFISHAHADHHVGLAKILAMRKTLDP**

**PplTrz1 (591) KYRNVSSTLVRIPG--------------WGSLLLEAGEGSWGQLARMYGDDIDSWTSG----------------VWELLRDLKCIFISHMHADHHIGLAKILAMRKLMNP**

**PhaTrz1 (574) RHRNVSATLIQIPN--------------HGNILLDAGEGTWGQLTRLFGDDPERKSG-----------------VWEVLRDLKCIFLSHAHGDHHIGAAKILAMRQKMSP**

**AbiTrz2 (516) QFRNVISTLLVTPD---------------GHIMLDCGEGTAQQLERRYGVE-----------------------TKNMLRDLKCIFISHAHADHHLGLISLLRRRRMLFD**

**MglTrz1 (580) KYRNVLSTLVYMPG--------------DGYLVLDAGESTYFQLARRFGPGELGWDGRIG--------------INKVLRELKMIFVSHIHGDHHMGVIRLLLERRRLSP**

**MlaTrz1 (516) KYRNVSGTLLHIPS------EADESHSGFHYALLDSGEGTLGQIKRNFGSR-----------------------WEEVLRDLKLIFISHLHADHHSGLATLLTARHKLSG**

**PgrTrz1 (506) KYRNVSSTLLHIP---------DGHGQKFNFVLLDAGEGTMGQIKRKFGLG-----------------------WKDTLRNLKMIFISHLHADHHCGLASLLAERSQLDN**

**AbeTrz1 (652) GPLSSSPSYM-----DITEEDVPRILKEKRLYVVSAQHMLQWMAEYTNVEN-----YGYD------------KVVPLSASSFEHPDGKT----------DYTYTIHRRQR**

**TruTrz1 (650) GPLSSSPSYM-----DITEEDVPRILREKRLYVVSAQHMLQWMAEYTNVEN-----YGYD------------KIVPLSASSFEHPDGKT----------DYTYTIHRRQR**

**MgyTrz1 (650) GNLPSTASYM-----DITEEDVPKMLSEKRLYVVSAQHMLQWMAEYTNVEN-----YGYD------------KVVPLSASSFEHPDGKI----------DYTYTIHRRQR**

**McaTrz1 (655) GSLSTIASNM-----DIAEADVSKILRERRLYVISAHHMLQWMAEYANVEN-----YGYD------------KIIPLAASSFEQSDGKT----------DYSYTLHRRQR**

**CimTrz1 (652) GIP---PQKT-----ASFETNLGQFLSEKRLCLVSDIHMLDWLAEYSHVEN-----YGYD------------KIVPLAATSYERSSGYI----------SSVLTLHRRDK**

**CpoTrz1 (652) GSP---PQKT-----ANFETNLGQFLSEKRLCIVSDIHMLDWLAEYSHVEN-----YGYD------------KIVPLAATSYERSSGYI----------SSVLTLHRRDK**

**UreTrz1 (655) GAL---PQRQ-----ADFETDLSRFLSERRLCVVSDIHMLDWLAEYSHVEN-----YGYD------------KIVPLAATSYVHPNRHI----------SSILTLHRRDK**

**AcaTrz1 (698) RTQSRTAPP------PEQERDLTKLLNEKRLFVVSNAKMIEWLAEYSSVED-----YGFD------------NLLLLAADPTVPDSFHY----------SHLGSDGQ---**

**AdeTrz1 (704) GALSSTAPV------PEPEYDLTKLLSEKRLFVASDAKMIEWLAEYSGIEN-----YGFD------------KLLPVEADTSFGDNFKY----------SHLSRDRQ---**

**PbrTrz1 (684) GTLSSASS-------PSPEQDLTKILSEKRLFVVSHAKMIEWLAEYAGVEN-----FGFD------------KITPLEVDSSAADSFRF----------SHLDQNSR---**

**AclTrz1 (667) PEGVPRTTTI--------ETDMSKILTDKRLFVISEEMMVGWLEEYAGVED-----YGFG------------KLVPLVAFPNYAN--GS----------IRTTLSYRHTR**

**AfuTrz1 (660) PNGVPGAGDV--------ETDMRKILQEKRLFVVSEEMMIGWLEEYAGVED-----YGFG------------KLVPLAAHPSYINPDSK----------NTTKFTYRHCR**

**NfiTrz1 (660) PNGVPRAGNV--------ETDMRKILQEKRLFVVSEEMMIGWLEEYAGVED-----YGFG------------KLVPLAAHPSYINPDCK----------IKTKFTYRHCR**

**AcrTrz1 (665) PEGIPQTNDV--------DMDMSKILKEKRLFVVSEQMMTGWLEEYAAVEN-----YGFG------------KVVPLSATPYLYQG--N----------IRTQFTYRHCR**

**AspTrz1 (666) PEGIPQTDEV--------EMDMSKILKEKRLFVVSEQMMTGWLEEYAAVEN-----YGFG------------KVIPLSASPYLHQG--N----------IRTQFVYRHCR**

**AflTrz1 (672) PNGDSQTSAL--------ETDMSKILKEKRLFLVSEENMIWWLEEYASAEN-----FGFG------------KLIPLSAYPVIQNR--A----------LRTKFVYRHCR**

**AorTrz1 (672) PNGDSQTSAL--------ETDMSKILKEKRLFLVSEENMIWWLEEYASAEN-----FGFG------------KLIPLSAYPVIQNR--A----------LRTKFVYRHCR**

**AteTrz1 (666) PDGVPHTTAV--------ETDMAKILNEKRLFVVSEEMMVGWLEEYAGVEN-----YGFG------------RLIPLSANPDLTNG--V----------YRTELLYRHCR**

**PchTrz1 (674) KDGFGSTASR--------ETDMARILQEKRLAVVSDNAMISWLEEYSQVED-----FGYD------------KLLTLSAHPDVEGS---------------KVTTV----**

**AniTrz1 (653) PSGVAQLSEP--------EKDIGKILEEKRLFVVSDLMMIEWLEEYAGVED-----FGFA------------KLTPLSAYLYRTG-DEG----------MKWSFKYRHCR**

**PmaTrz1 (642) GARS-GSEMP--------SENIADILKQKRLAVVGESMYIQYLEEYSGVED-----FGYE------------KTLPLSVLPPTRDRP-------------YTSVVYRYTR**

**TstTrz1 (646) GARSPSTGSA--------PEDVTEVLKQKRLAVVGETMYIQYLEEYAGVEN-----FGFE------------KILPLAVLPPAIEKP-------------YTSLVYRYTQ**

**AbrTrz1 (642) NAVPNSKP--------LDTSASDIDTSAYGLSVISHSGMLQWLSEYSAIED-----FGYS------------RILPLQITANEARKSS----------------------**

**CheTrz1 (642) NSVPNLQP--------LGNTVGGTDISAYGLSVISHSGMLQWLHEYSAIED-----FGYS------------RILPLQITANEHGSSS----------------------**

**PtrTrz1 (642) NSVPNPHT--------LGNSTDGIDVKSYGLSVISHRGMLQWLNEYSSVED-----FGYS------------RILPLQLTPNEKGKSS----------------------**

**PnoTrz1 (642) GGVPNKEK--------LDAATVLNNSSQYGLSVISHTGMLQWLYEYSSVED-----FGYS------------RILPLEVSPNETGRGS----------------------**

**MfiTrz1 (670) NGKPAAPVTP-------TQDWKEIFEDQ-RLAIISEPAMQYWLYEYSALED-----YGYS------------RLAPLSMTPANLRTGQ-----------PSRMSWFI---**

**MycTrz1 (654) NSQPAPPASPRITADYGELDWPSVFNGQNRLAVVAEAAMQNWLEEYSAVED-----YGYS------------RLAPLNLSAVRFFSRTS----------QSTLSWYI---**

**SscTrz1 (629) KN-------E--------------ATRNNKLAIASDHGMIHWLTEYSEVEH-----YGFE------------RLELIEMTPAVNNLYE----------------------**

**CglTrz1 (737) EGN-------------------------K-LALIATQKYQDFIREFHQVQD-----LSPE------------RIVPITLRSP-GNPIPG----------RHAQATFPAD-**

**MthTrz1 (588) EEEGG-----------------------K-LALIATQKYQDFVREFHEVQD-----LGLD------------RIVPVVLRCA-GRPLPG----------RHAEPPAVPD-**

**TteTrz1 (628) PADR------------------------K-LAIIATPKYQDFVREVQSVQH-----LSGE------------RLLHVTLRSTSGRPMPG----------TLTKTSFPDG-**

**PanTrz1 (594) GTD-------------------------ALLGVIATGGFHSWMLEYDGVEP-----LGLD------------RVVGIVPYRG-GQPNAD----------LGWKFPPTLN-**

**NcrTrz1 (839) ALP--------------------ADEAPKPLTIISTTKYIGFLHEYKDVEP-----LDWA------------NIKFLTLIGTGRKPTDD----------PSGPPSADLGP**

**NteTrz1 (839) ALP--------------------ADEAPKPLTIISTTKYIGFLHEYKDVEP-----LDWA------------NIKFLTLIGTGRKPTDD----------PSGPPSADLGP**

**NdiTrz1 (839) ALT--------------------ADEAPKPLTIISTSKYIGFLHEYKDVEP-----LDWA------------NIKFLTLIGTGRKPTDD----------PSGPPSADLGS**

**SmaTrz1 (854) AH----------------------KEEAKPLTIISTPKYIGFLHEYKDVEP-----LDWT------------NIKFLTLIGTGRKPADD----------PNGPPSADLGP**

**MgrTrz1 (711) SLSATTSSG-----------------TPPFLTVAGPTGLMNYIREYQNVEY-----ISN-------------R-LILRGVAR--PNK-----------------------**

**FgrTrz1 (634) NN------------------------SNAKLAISCVARYQALLEEVSQVED-----IGFH------------RLHFPNAEAD----TNT----------KRGHGRYELK-**

**FoxTrz1 (631) ED------------------------TNAKLAVSCVSRYKALLEEVSQVED-----IGFH------------RLHFPNCNST----KPD----------KLNNGRFVIK-**

**FveTrz1 (634) ED------------------------TNAKLAISCISRYKALLEEVSQVED-----IGFH------------RLHFPHCSST----KPD----------KYNTGPFVID-**

**NhaTrz1 (633) QD------------------------SNAKLAISCISRYRNLLEEVSQVED-----IGFH------------RLHFPSCS------QSD----------KFNTGRREVK-**

**TatTrz1 (640) KDN-----------------------SNATLAISCIGRYRVLLEELSQIED-----IGFH------------RLRFPSCPFP---KEKD----------RDVTTKEDLG-**

**TviTrz1 (638) KDN-----------------------SNATLAISCIGRYRILLEELSQIED-----IGYH------------RLRFPSCPWP---KEKD----------RDLTTREDLG-**

**TreTrz1 (644) KDG-----------------------SNATLAISCIGRYRIMLEELSQVED-----IGYH------------RLRFPSCPYP---KEKD----------RDLTTREDLLG**

**AgoTrz1 (556) EDPE------------------------AKIYLVAPWKYDTFIEEWFTLED-----RSIL---E--------RVNYISCEHFLDGPKRKPQGYQPSLGYPEEIDTTARNR**

**CanTrz1 (566) NDEN------------------------AKIYIVTPWQYKKFVDEWLNYED-----ESIL---S--------RINYISCEHFIHGNYVR-----------KEIKPLDINE**

**SceTrz1 (562) DDET------------------------SYIYVVTPWQYHKFVNEWLVLEN-----KEIL---K--------RIKYISCEHFINDSFVR-----------MQTQSVPLAE**

**ZroTrz1 (556) DDD-------------------------EVIYVITPWQYNRFINEWLLLED-----ANLL---N--------RIRYISCEHFVNGSYVR-----------RETKPIPLEE**

**VpoTrz1 (562) EDKD------------------------AIIYLVTPWQYNKFIHEWLNLEK-----PEIL---E--------RIRYISGEHLVSGNFVR-----------REIQPITIEE**

**LthTrz1 (558) TDPS------------------------AVLYIVVPWQYNIFVKEWLSLEN-----SEIL---E--------RLRYVSCEHLVDGGYVR-----------KELKPVPFED**

**KlaTrz1 (564) HTA-------------------------DKLYVITPWQYNIFVREWFSLED-----PAIL---S--------RIEYISCEHLKAGDDIR-----------KQTKPLSMDV**

**PpaTrz1 (542) ----------------------------TDLYLIIPWQYETFLKEWSRLPN-----QDDLPDLS--------RLKIFSCEQFHTNPHPS------------LEQVSFEQM**

**CalTrz1 (548) DES-------------------------KLLYLVVPWQFITFLKDWYSLES-----QYNKVFDMN-------RLVCISCEDFMMDNR------------TPEFIKIDMDK**

**CduTrz1 (548) DES-------------------------RILYLVVPWQFITFLKDWYSLES-----QYNKVFDMN-------RLVCISCEDFMMDNR------------TPEFIKIDMDK**

**CtrTrz1 (547) DKS-------------------------KTLYLVVPWQFITFLRDWYSLES-----QYHPTFDIN-------RLVCFSCEDFCLETR------------AAEYQKMSMDK**

**CpaTrz1 (544) GSD-------------------------KKLYLIIPWQFITFVNDWYSLET-----QYNHAVDLT-------RLQMFSCEDFLKEGR------------LPEYQKLTIDE**

**CguTrz1 (547) STA-------------------------KKLYLILPWQYNHFLSEWYRLEE-----YFSADVDIN-------RLKYLSCEEFLRDR-------------APEYKQFSIQE**

**PguTrz1 (547) STA-------------------------KKLYLILPWQYNHFLSEWYRLEE-----YFSADVDIN-------RLKYLSCEEFLRDR-------------APEYKQFSIQE**

**DhaTrz1 (550) DSE-------------------------KKLYLVIPWQYNNFMKEWYSLEE-----QIYDKIDLN-------RIVYLSCEDFIKDR-------------QPQFQQVDIDE**

**PstTrz1 (549) EDH-------------------------RKLYLIVPWQYETFISEWYKLEG-----QLNDSVNLD-------RIEFLSCEDFLPDR-------------LPKYHKIDIDD**

**CluTrz1 (550) NNT-------------------------DKLYLVIPWQYNNFITEWYRLEQ-----YTSN-IDMS-------RIVYLSCEDFMRTP-------------EAQLKQFTLDE**

**YliTrz1 (600) ---------------------------DETLSVVGPQSYKRFLEECANFTP-----------------EMQARIRYYGCWAFLEKHR-----------------------**

**AmaTrz1 (672) --------------------------------LVGPWRLMHYLRELDSASVGRGGRRGSNDAGNADASPASPAVPSVTKSPFAKAVPVP---------------------**

**BdeTrz1 (537) KTS-------------------------KRLLLVAPTQMWNWLCEYTLVQK-----LPLD------------RIEFVDAATTKWKREDG---------------------**

**SpuTrz2 (578) SRN------------------------PQPLIIVAPARYQIWLEEYADCEE-----FGLK------------HVLFLNAEDVRWNASG----------------------**

**SpuTrz1 (636) KAG----------------------KPAPTICIVGPPQYRIWLDEFDDCEP-----LGLAG----------DQITFLPCHDLLPKASTS---------------------**

**ScrTrz1 (582) T--------------------------SSKLFLSGPPQFESWLKDYSKLDK-----LDLS------------RIVFLSNSATRRDRT-----------------------**

**SocTrz1 (583) T--------------------------SSKLFICAPPQFESWLKDYSKLDK-----LDLS------------RIVFLSNSAVRRDRT-----------------------**

**SpoTrz1 (596) G--------------------------RSKLFITAPPQFEFWLLEYSRIDY-----LPLS------------NIVFISNSALRTDRK-----------------------**

**SjaTrz1 (548) P--------------------------DQVLFITAPQAFYNWLSEYATLDS-----LPMD------------RVVFINNTALRNDRPN----------------------**

**ScrTrz2 (492) G-------------------------ADTTLTVVCPKSLRFWVS----------------------------RICDSTKLSHIVERIR----------------------**

**SocTrz2 (492) S-------------------------SDSKLTVVCPRSLRFWLS----------------------------RVSESARLSQIIKRLH----------------------**

**SpoTrz2 (494) N-------------------------NSMHINIIGPKFLWQWLQ----------------------------RLKSPANLQALLNRII----------------------**

**SjaTrz2 (482) P---------------------------YKVLLICPLDIKNWC-----------------------------NFLFPSIHQAIQ--------------------------**

**CneTrz1 (623) TTP---------------------------LYIVAPYLIALSLQETATWQA-----AGTEQGLKNVRFL-CVERLGERMSADIPGEDDSQKIEWTQ----KEGGGKGR--**

**TmeTrz1 (612) HTP---------------------------LYIIAPIQIALSLQESYKWQL-----DVPREALRNLVFLPSNRIQGMISRDSLSLVKRVDG---------GSGSSVGVGK**

**AbiTrz1 (591) LP-------------------------DHPLYVVSVRSVHRTLKEVHELED-----IGLNDDPRE------NGVVQVISEALNTRWNYDDEVALRRAG------------**

**CciTrz1 (700) PP-------------------------ENPLYLVSIRIVQLYLQEVQQLYD-----LGIRPSINTIEGA-GNAVIPIQSEALHYARPG----------------------**

**ScoTrz1 (646) PP-------------------------KDPLFVYSIRRVHLYLRELQMLQD-----LGIDDP------A-GNGVYGMLSEGLHWRNTGS---------------------**

**LbiTrz1 (636) LP-------------------------TEPLYIVTIRGVNMYLRELSDIQD-----LGLNDPS-------GNGVVQIQSEALHYRRTD----------------------**

**PosTrz1 (647) PP-------------------------TQPLYLVALRAAHVYLREYSDIED-----LGLFDSS-------PSGVVTIMSESLHWKRPER---------------------**

**SlaTrz1 (656) PA-------------------------DSPLYVVANRTVFMYLYEYAALED-----LGFLS---------HNGVVPIFNDAIHWKQNN----------------------**

**HanTrz1 (644) PP-------------------------EDPLYLVSVRVVHLYLREQADLED-----LGLADSPVT-----TNGVVTVMSDVINYRGRP----------------------**

**PplTrz1 (671) PP-------------------------SQPLYVVGLRRTLVYLREQSEVED-----LGVDQADG-------NGIITILSDVLNWRPVL----------------------**

**PhaTrz1 (653) RP-------------------------TGPLYVVSIRPVLLYLHEYQEFED-----LGLGRYDG-------TGVETVLSDAIHWDGFT----------------------**

**AbiTrz2 (588) RP-------------------------KQPLYIVATRLVHLFLKEYQDLED-----IGLYDDPTQDG------IIHILADALNYRHDEYPITG-----------AWR---**

**MglTrz1 (662) SEP---------------------------LVLVTNNFTRFYLYEYDLIEQLGVRDGSVLALENEALDWEHGIDPDPLSTRATATATTAR--------------------**

**MlaTrz1 (597) ---------------------------VSPLVLVSQYGVNLYLREKSLVEP-----MGIEQG----------LVHWIDVEKLVGARAS----------------------**

**PgrTrz1 (584) ---------------------------CAPLALLCQYGIYLYLSEKAVIEP-----LGLSIG----------RVQWFNSEHLLQSSDK----------------------**

**motif III motif IV**

**AbeTrz1 (730) NGAAIPDSDG------KYLGTRLKFDDENSPLTELLKQGTGLDAILTTPVSHCQGAKAVSLIFPTG-------------------FKVSYSGDCRPS--TRFIEMG----**

**TruTrz1 (728) NGAAIPGKDG------KFLGTRLKFDDKNSPLTELLKQGTGLDAILTTPVSHCQGAKAVSLIFPTG-------------------FKVSYSGDCRPS--TRFIEMG----**

**MgyTrz1 (728) NGAAISDKDG------KHLGTRLKFDDANSPVTKLLKQGTGLDAILTTPVSHCQGAKAVSLVFPTG-------------------FKVSYSGDCRPS--TRFIDIG----**

**McaTrz1 (733) NGAAISDKG-------KHLGTRLKFDDEQSHLTTLLKQGTGLDAILTTPVSHCQGAKAVSFVFPTG-------------------FKVSYSGDCRPS--TRFIEMG----**

**CimTrz1 (727) NGLVQEQFG-------ENRGEWINFDSPKHQFMSPFQAATGLSSIFTVPVSHCQGAKAVSFTFPSG-------------------LKVSYSGDCRPS--EAFTRIG----**

**CpoTrz1 (727) NGLVQEQFG-------ENRGEWINFDSPKHQFMAPFQAATGLSSIFTVPVSHCQGAKAVSFTFPSG-------------------LKVSYSGDCRPS--EAFTRIG----**

**UreTrz1 (730) SGLVIESTG-------ENRGEWFNFDFPNHPSTTLFRSATGLKSIFTVPVSHCQGAKAVSFTFPSG-------------------LKVSYSGDCRPS--EAFTRIG----**

**AcaTrz1 (772) --PLLDYHG-------KPLTTFVSFNPDRSPLALQLQQATGLSTLLTVPVMHCQGSMATSFVFPSG-------------------LKVSYSGDCRPS--RDFARVG----**

**AdeTrz1 (778) --PILDENG-------NPVKIHLSFNPDRSPFATQLQQATGLSSLLTVPVMHCHGAMATSFVFPSG-------------------FKVSYSGDCRPS--REFARIG----**

**PbrTrz1 (757) --PILNERG-------NPMKTLLSFNPDQSPFAAQLQAATGLSTLLTTPVMHCNGSKATSFVFPSG-------------------LKVSYSGDCRPS--RNFAKIG----**

**AclTrz1 (740) DDGSYPGYEQDGSRPQITTLDFHDKNSP---LTPLLQRATGLTDILTTRVPHCRGAMAVSLIFPDS-------------------FKVSFSGDCRPS--NSFAAIG----**

**AfuTrz1 (735) DDGSYPGWEADGSRPQTSTLDFNDDSSP---LAPLLRQATGLADILTTRVSHCRGAMAVSLVFPDG-------------------FKLSFSGDCRPS--TNFATIG----**

**NfiTrz1 (735) DDGSYPGWEADGSRPQTSTLDFNNDSSP---LAPLLRQATGLADILTTRVSHCRGAMAVSLVFPDG-------------------FKLSFSGDCRPS--TNFATIG----**

**AcrTrz1 (738) ADGSYPNHQLDTAKPSTTSLSFNDNSSP---LSALLREATGLSDLLTTRVSHCRGAMAVSLVFPNG-------------------FKVSFSGDCRPS--AGFATIG----**

**AspTrz1 (739) GDGSYPGHQLDATRPATTSLSFHDQSSP---LTSLLREATGLSDLLTTRVSHCRGAMAVSLVFPDG-------------------FKISFSGDCRPS--AGFATIG----**

**AflTrz1 (745) ADGSFPGQEVETYRPRTTELSFDDESSP---LTPLLREATGLTDLLTTKVSHCRGAMAVSLVFPNG-------------------FKLSFSGDCRPS--PSFAAIG----**

**AorTrz1 (745) ADGSFPGQEVETYRPRTTELSFDDESSP---LTPLLREATGLTDLLTTKVSHCRGAMAVSLVFPNG-------------------FKLSFSGDCRPS--PSFAAIG----**

**AteTrz1 (739) ADGSYPGS-ESDEKPQITSLRLDDDTSP---LTPLLRKATGLSNLLATKVSHCRGAMAVSLVFPDG-------------------FKISFSGDCRPS--PIFAAVG----**

**PchTrz1 (740) FS--HPLP-----NRRRSQLDFTDKSSP---LTPLLKSATGLADILTCRVKHCKGALAVSLVFSNG-------------------FKVSYSGDCRPS--DKFAAIG----**

**AniTrz1 (727) ADGSYRGREVEHIKPEQSELKFNCKGSPGEELSAKLRKATGLSDILTAYVSHCRGAMAVSLIFPDG-------------------FKVSFSGDCRPS--PTFVTIG----**

**PmaTrz1 (713) PDGTSKDDA-------QN-SAYIRFDSTENYLSGLLRAATGLTGLNAVFVNHCRHAMGLTLEWEDG-------------------FKVSYSGDCRPS--KNFAEMG----**

**TstTrz1 (718) PDGTTQEDG-------RN-PVFLRFDTPKSHLTPLLQATTGLVSLNAVFVNHCRHAMGLSLEWEDG-------------------FKVSYSGDCRPS--MNFAQMG----**

**AbrTrz1 (705) TLSILNSFG-------------KNESENPIVQRKEYERLFGFQDIQAAKVVHCNGAMAVSMTFPSS----------PSDLQGVKPLKVSYSGDCRPS--YHFSKIG----**

**CheTrz1 (705) RLSIMNTFG-------------KERTENPEVHRSMYEKFFGFVDIQTANVSHCHGSMAVSITFPRS----------PSDPEAVKPLKVSYSGDCRPS--WHFSKIG----**

**PtrTrz1 (705) TLSVLNSFE-------------KLESENPSIHRREFEQLLGFEDIQSAHVAHCHGSMAVTFTFPRS----------PSDPEDVKPLKVSYSGDCRPS--YHFGRVG----**

**PnoTrz1 (705) LLNILNSFN-------------AEQNYDTTIKRNQYPQLFGFEDIQTAKVVHCHGAMAVCITFPRS----------PEDPHNLKPLKVSYSGDCRPC--RHFARVG----**

**MfiTrz1 (741) PPSELKGLS-------------KSDYHAKLDEHVIDHSLLNLQDIQAVAVQHCHGARAISITLPSG-------------------FKVSYSGDCRPN--TPFTQIG----**

**MycTrz1 (734) PPSQLKNLS-------------HQAYNARLEQNYIDPALIGLSDIQAASVKHCHGARATSLTLNTG-------------------FKVSYSGDCRPC--KAFWQIG----**

**SscTrz1 (679) ------------------------------EFTAAQTEAFGLSSIQACQVSHCHGALAVAFNFPNG-------------------FKVAYSGDCRPS--QDFVRIG----**

**CglTrz1 (792) -----------------------------FDP-----QTLQLPQIESCFVDHCYEATAVVLTFPD------------------TGLKLAYSGDCRPS--RQFAELG----**

**MthTrz1 (645) -----------------------------EDA-----RAARLPRVEACFVDHCYEATAAVLTFPD------------------TGLKVAYSGDCRPS--RPFAELA----**

**TteTrz1 (685) -----------------------------VDP-----EALGLPRIEACFVNHCYEATAVVLTFPD------------------TGLKIAYSGDCRPS--ATFAELG----**

**PanTrz1 (650) -------------------------------QSECNTMQERFPKVEICWVDHCQNATAIVMTFAP------------------SGLKIAYSGDCRPS--KKFAELG----**

**NcrTrz1 (902) GNNHFNMLPG----------TLCALQPASFRTAATLEAQTGLASIDACFVDHCLGATAAVFTWP-------------------SGLKISFSGDCRPS--DCFAQIG----**

**NteTrz1 (902) GNNHFNMLPG----------TLCALQPASFRTAATLEAQTGIASIDACFVDHCLGATAAVFTWP-------------------SGLKISFSGDCRPS--DCFAQIG----**

**NdiTrz1 (902) GNNHFNMLPG----------TVCALQPASFRTAATLEAQTGLASIDACFVDHCLGATAAVFTWP-------------------SGLKISFSGDCRPS--DCFAQIG----**

**SmaTrz1 (915) ANNHFNMLPG----------TVCTLQPAALRNADVLESQTGLASIDACFVDHCLGATAAVFTWP-------------------SGLKISFSGDCRPS--DAFAHIG----**

**MgrTrz1 (760) ---------------------TTRIAEPITFSETQQADLGGLTRIDVAKTDHCFDSFATVFTWEKT------------------GLKVAYSGDCRPS--EEFVQIG----**

**FgrTrz1 (688) ------------------------------------DNTFGLKAITRIPVPHCWLAMATELELT-------------------SGLRIAYSGDCRPS--TEFATTC----**

**FoxTrz1 (685) ------------------------------------NGDFGLRAIKRIPVPHCWLSYGTELELT-------------------SGLRIAYSGDCRPS--DEFAQEC----**

**FveTrz1 (688) ------------------------------------NGDFGLRVIKRIPVPHCWLSYGTELELT-------------------SGLRIAYSGDCRPS--NEFAREC----**

**NhaTrz1 (685) ------------------------------------DDSFGLRSITRVAVPHCWLSFATEIELT-------------------SGLRIAYSGDCRPS--DDFAREC----**

**TatTrz1 (696) ----------------------------------DEN--FGLASIKRVPVPHCWRSFGTQLELT-------------------SGLKIAYSGDCRPS--KLFAQEC----**

**TviTrz1 (694) ----------------------------------EEN--FGLASIKRVPVPHCWRSFGTQLELT-------------------SGLKIAYSGDCRPS--KLFAQEC----**

**TreTrz1 (701) ---------------------------------DDENNNFGLASIKRVPVPHCWRSYATQLELT-------------------SGLKIAYSGDCRPS--KSFAQEC----**

**AgoTrz1 (626) LNLLVEKNGEKR-----RYVSDNSTVNIPWRHIRNMQKDLQIASFKTCRAIHCEWAYSNAISFYVG-------------SSSKEQFKVSYSGDTRPNFDKFAKGIG----**

**CanTrz1 (625) FEAVISENTNKKRK--LQLSLDDTSSFKDYTSIKMMCKDLKIFKFETCRAKHCDWAYSNTITFYKN-------------SFNSDLFKVSYSGDTRPNLERFAYEIG----**

**SceTrz1 (621) FNEILKENSNQESN--RKLELDRDSSYRDVDLIRQMYEDLSIEYFQTCRAIHCDWAYSNSITFRMD------------ENNEHNTFKVSYSGDTRPNIEKFSLEIG----**

**ZroTrz1 (614) YADGLESADKKR----RKLEIDSNSSLRDLDTIKKMYKDLRIFNMQTCRAKHCDWAYSNTISFFMS-------------SSSKKLFKMSYSGDTRPNIEHFSKDIG----**

**VpoTrz1 (621) FDSMLKLPAKKK----QKLSFVEDSSYRDLKAINTMYQDLKIKSFQTCRAKHCNWAYSNTITFYTN------------SNPSNNTFKVSYSGDTRPNIKSFAQDIG----**

**LthTrz1 (617) FAAATKPALKKR-----RLEFDENSSFRDRETITQMYRDLKILRFQTCRAKHCDWAYSNSISFFTG-------------SDSSGIFKVSYSGDTRPNVEKFSKTIG----**

**KlaTrz1 (622) DGTYATK--GRK-----DLEHDEESAVKDTAKIKKLMQNLRLMSFRTCPAIHCDWAYCNSISFFLS-------------ARSKELFKLSYSGDTRPNFNQFARGIG----**

**PpaTrz1 (599) IN--------------AKQTITPKKIPLQRISTETMFKDLGLTGIQTCSAIHCEWAYSVGFTFDIG----------------GDAFKVSYSGDTRPN-HRFAFEIG----**

**CalTrz1 (609) FEEFYDEGGLHK------PIPRDRLLSVDHKKIKNMYETVGLNSVTTVRALHCAWAYSTTFDFKLN--------------ENGETFKISFSGDTRPN--PRFSECG----**

**CduTrz1 (609) FEEFYDKGNLHK------PIPRDRLLSVDRKKVKNMYETVGLNSVTTVRALHCAWAYSTTFDFKLN--------------ENGDTFKLSFSGDTRPN--PKFAECG----**

**CtrTrz1 (608) FEEYFDKKLLHK------SVPKGKLNSLDLESIQEMYDSIGLNSIATVRALHCAWAYSSIFDFKLN--------------NRGETFKVSFSGDTRPN--PKFSRCG----**

**CpaTrz1 (605) FEQAYDCGDRHR------SINKAPLQPVDNDAIAQMYEAIGLVSISTVRALHCAWAYSSTFKFILD-------------SNRDQHFTISFSGDTRPN--PKFCSIG----**

**CguTrz1 (607) FEEQFDRKDLKK------SIAKEPLAPRSTALIEELYEELKLVKVSTVRAIHCYWSYSISLEFKLD---------------PTETFKISYSGDTRPN--PKFVDIG----**

**PguTrz1 (607) FEEQFDRKDLKK------SIAKEPLAPRSTALIEELYEELKLVKVSTVRAIHCYWSYSISLEFKLD---------------PTETFKISYSGDTRPN--PKFVDIG----**

**DhaTrz1 (610) FERKYDNDEFNQ------VIPREKLAPKNHVLINDLYKNLGINSIQTVRAIHCYWSYSISIDFALP---------------SEESFKVSYSGDTRPN--PKFVDIG----**

**PstTrz1 (609) FEYLYDNNDLNR------IVAKSPLEPLNRSAISRLFADLRIKEIATVRALHCAWAYSVSITFDLE---------------DNESFKVSYSGDTRPN--PKFVDIG----**

**CluTrz1 (609) FEEKYDSNRLTD------RIPKEDSALPKTSRIDMLYRDLNLANIRTVRAIHCYWSYSVSLCFSLS---------------SSETFKVSFSGDTRPS--TRFIESG----**

**YliTrz1 (643) -------------------------------KMSALPEVPGLTSIKTCWAHHCEQSFCVEFGFQLD---------------DSETFNVAYSGDTRPI--EAFSEMA----**

**AmaTrz1 (729) -------------------SAAKSPFDKAVPAPSMEAEALSLQSMYTANVIHRSFSYGVRVQSAID-----------------PKLSIVFSGDTRPC--PKLVQLGQVSA**

**BdeTrz1 (584) -------------------------ANQSYATNNSVFLKAGLSKMTTVGVNHCPEAYAIVAETISG-------------------FKFGFSGDCRPS--HDFAIAG----**

**SpuTrz2 (625) -------------------TDGRDEDVEKTTTINTLKKTLHLKDIHTVGVHHCPWAYALVLDTEAA-------------------GKVVFSGDCRPS--EDLVNAG----**

**SpuTrz1 (688) -----------------------------STVLDRLKEDLGLSAIETVQVHHSHYSYAVSLAHQSG-------------------YKIVYSGDCRPN--LDLMTVG----**

**ScrTrz1 (626) ------------------------FTEVDQIKYQMLIQELDLAAFETVAAVHCPFSYCMQFTG-NE------------------GWKIAYSGDTRPS--EEFIDIG----**

**SocTrz1 (627) ------------------------LTDEDLIKYRLLLQELDLTSFETVAAVHCPFSYCMQFTG-NE------------------GWKIAYSGDTRPS--EEFIEIG----**

**SpoTrz1 (640) ------------------------PSALESSRLSSLFKEFDLVSFRTVPAIHCPYSYCMEITN-SS------------------GWKIAYSGDTRPS--EDFANIA----**

**SjaTrz1 (593) ------------------------ITEACSREISHLFNSLGLNSLLTVPAIHCAYSFCVAFTL-SN------------------GCKIAYSGDTRPC--LPFCNIG----**

**ScrTrz2 (527) ------------------------FVNASTVHAGNEYSLSQSLSLYTVPSIHIYDSHSVILSH-KS------------------NGNLVYSSDTRPN--MRLAKAG----**

**SocTrz2 (527) ------------------------FVDASTVGIGNEYCLSENLSLYTVPSIHIADSHSVVLSH-KS------------------SGKLVYSSDTRPN--MKLARAG----**

**SpoTrz2 (529) ------------------------FIIAKET-VTTPLQLTSDLSISSVPSIHINDSYSCIISH-TK------------------YGKLVYSGDTRPN--EKLVKAG----**

**SjaTrz2 (510) --------------------------FATPSTIPKTLETEPSWTWTTVQALHTKNSYSIVLSHRSL-------------------GKIVYSGDTRPN--SSLIKVG----**

**CneTrz1 (694) --------------WPFVPLHGFSDSKIQRHYLRQLFVDLGLTAIYVPSVPHRGRAYGLVLEGNPV----------GKDGVNRKGWKIVYSGDTKPS--QKLVEAG----**

**TmeTrz1 (681) DGS---EGLGRGWPFGLLYSPELASEQTLQTNLRALFDALSLEEIQIPLVEHRGRAWGLVLKHQTG-------------------WKVVYSGDTMPS--ENLIQAG----**

**AbiTrz1 (653) ------------------GNEPWLDIQLSKQNALDICRHLDLSSFETMDVEHRTKAFGVFFRHRDG-------------------WSIAFTGDTMPT--QNVVKVA----**

**CciTrz1 (757) --------EYASGYWAVNGTEPWLDPKRSVTLARSMCEALGLRSFSTVDMRHGTRCYGLVVKH-RD------------------GWSIAFSGDTEPT--DSIVHAG----**

**ScoTrz1 (698) --------YHINGRYTLGGSEEWTDINLTTKRYRQMCELLGLRSFFAVDMLHRTRCYGCVIKH-ND------------------GWSISFSADTMPT--DRLVWAG----**

**LbiTrz1 (687) --------YATGGMWQVGGNEPWVDREASRKHSSDMCRALGLESFKTVDVYHNARCYGCVVTH-VD------------------GWSIVFSGDTQPT--DNLVRAG----**

**PosTrz1 (699) --------YQATGMWQLGGNEPWTDIQRSQDAAQEMCSSLGLKSFRTVDVYHRTRCYGAVIDH-AD------------------GWSIVFSGDTQPT--QNLVWAG----**

**SlaTrz1 (705) ---------PNATRWMFESEE---EQRSMQLAVSDLCASLNLVSLTTVDVEHRARCHGIIIRH-SD------------------GWSIVYSGDTVPT--HRLVRAG----**

**HanTrz1 (697) ----------SP-FPGPK--DGWQDPLHSRLAAEMCCDALGLHSLETVDVKHRTRAYGAVMTS-RE------------------GWRVVYSGDTMPC--SSLVEAG----**

**PplTrz1 (722) ----------KH-WREHKDSEPYEDEERSQQAADDMCHALGLKSFTTVDVWHKTPCYGVVIKH-SD------------------GWSIVFSADTSPT--YNLVRAG----**

**PhaTrz1 (704) ----------RG-YGGMS-DMPWQDETLNHLTKSAMCDALGLSDFLTCFVDHRVRAYGCVIKH-NDRWSITYGFFLHHKDIVIANFGVRFSGDTRPT--DHLARAG----**

**AbiTrz2 (648) ----------------MLGKEPWLDIASSRQHNLDMCEKLNLASFETIDVYHSTRAFGTLIRHKAE-------------------WSVAYSGDTRPT--INLVKLG----**

**MglTrz1 (725) ---------------RLSNTKEDNAAAQTERHLATLKRLTNLTGVRTAAVSHRAGHCYGLILTHKC------------------GWKFVFSGDTMPC--NSLVQAG----**

**MlaTrz1 (643) ---------------------------NQDQKAKYEEFLPKGMEIETVRVIHWGKCFGLCLSNRIQ------------------GWKIVFSGDTKRC--PELIEAG----**

**PgrTrz1 (630) ---------------------------RLATIRRIQALISSGFEIETILVNHWGKCFGVCIEQKTE------------------RWKIVFSGDTRPC--QALIDAG----**

**HEAT HST motif V**

**AbeTrz1 (809) RGSTVLIHEATFDDNMLS--DAVAKRHSTVSEAMTVGLRMEAKVIVMTHFSQRYRKMPDIANAKEVH-----------------IQPPKGWNNKGNT-------------**

**TruTrz1 (807) RGSTVLIHEATFDDNMLS--DAVAKRHSTVSEAMTVGLKMEAKVIVMTHFSQRYRKMPDIANAKEVH-----------------IQPPKGWNNKGNT-------------**

**MgyTrz1 (807) RGSTVLIHEATFDDNMIS--DAVAKRHSTVSEAMTVGLRMEAKVIVMTHFSQRYRKMPDIANAKEVH-----------------IQPPKGWNNRGNN-------------**

**McaTrz1 (811) RGSTVLIHEATFDDNMIS--DAVAKRHSTVSEAMTVGLKMEAKVIVLTHFSQRYRKMPDIANAKVVH-----------------IQPPKAWNNRAN--------------**

**CimTrz1 (805) RDSTVLLHEATFEDDMFK--DALAKRHSTLSEALMVGKEMHAKVIVLTHFSQRYREMPNIEKAKKTG-----------------FVPKFRHRKSPG--------------**

**CpoTrz1 (805) RDSTVLLHEATFEDDMFK--DALAKRHSTLSEALMVGKEMHAKVIVLTHFSQRYREMPNIEKAKKTG-----------------FVPKFRHRKSPG--------------**

**UreTrz1 (808) RDSTVLLHEATFEDDMIS--DALSKRHSTLSEALMVGKEMRAKMVVLTHFSQRYREMPNIEKAKKTG-----------------FVPSFNQRRSN---------------**

**AcaTrz1 (848) ADSTVLIHEATFEDDMLQ--DAQAKRHSTCGEALRVAKQMRARNVILTHFSQRYTHKPTIPRLKIWA-----------------SSHLYSPPRSPS--------HPRARS**

**AdeTrz1 (854) ADSTVLIHEATFEDDMLR--DAKAKRHSTCGEALRVAERMRARNVILTHFSQRYAHKPTVPRLKIWD-----------------ASNCGSPSRSPSRSPSRSPSSPAARS**

**PbrTrz1 (833) KDSTVLIHEATFEDDMYQ--DARAKRHSTSGEALQIAKLMRAKNIVLTHFSQRYTHKPTIPRLQIWA-----------------SSSGSRSASRSPR---------SCSN**

**AclTrz1 (822) RDSTVLIHEATFQDDMAM--SAVLKKHSTTSEALEVGRLMDARTVVLTHFSQRYQKVAHVEQ----------------------GSGPASTATTAQ--------------**

**AfuTrz1 (817) RDSTVLIHEATFQDDMAV--SAIAKKHSTTSEALEVGRMMQARTVVLTHFSQRYQKVAHVDH----------------------GAGAGTAKPEPE--------------**

**NfiTrz1 (817) RDSTVLIHEATFQDDMAV--SAIAKKHSTTSEALEVGRMMQARTVVLTHFSQRYQKVAHVDH----------------------GAGAGTAKPDPE--------------**

**AcrTrz1 (820) RGSTVLIHEATFQDDMAV--SAIAKKHSTLSEALEVGRLMNARAILLTHFSQRYQKLARIEE----------------------GANSSSSTSRGE--------------**

**AspTrz1 (821) RNSTVLIHEATFQDDMAV--SAIAKKHSTLSEALEVGRLMNARAILLTHFSQRYQKLARVEE----------------------APSRTANKPAAE--------------**

**AflTrz1 (827) HGSTVLIHEATFQDDMGV--SAIAKKHSTTSEALEVGRRMEARAILLTHFSQRYQKIAHVEK----------------------NQVPTKR-------------------**

**AorTrz1 (827) HGSTVLIHEATFQDDMGV--SAIAKKHSTTSEALEVGRRMEARAILLTHFSQRYQKIAHVEK----------------------NQVPTKR-------------------**

**AteTrz1 (820) HGSTVLIHEATFQDNMQM--SAIAKKHSTVAEALEIGRRMEARSILLTHFSQRYQKVAHLDK----------------------QDASVAAKLDRQVV------------**

**PchTrz1 (815) QGSTVLIHEATFQPDMVG--SARAKRHSTSSEAMEVGRRMQARAVLLTHFSQRYQKVAFVEK----------------------RNSGKFPSKR----------------**

**AniTrz1 (812) QDSTVLIHEATFSDDMVG--SALAKKHSTAQEAIEVGRKMRARTILLTHFSQRYQKIAHFNQPKELVQDKSVTARDFRAHKSIRRKQETAAAEAASTD------------**

**PmaTrz1 (790) KDSTLLIHEATFQDDLHG--QAVAKKHSTISEAIAVGSWMNAKTVLLTHFSQRYAKISKLEDI------------------------GRRPQAQQGKA------------**

**TstTrz1 (795) RDSTLLIHEATFQDDLQG--QALAKKHSTTSEAMMVGRWMNAKLVLLTHFSQRYAKISKMEDS------------------------SRRPRAQHDNP------------**

**AbrTrz1 (786) TDTTVLIHEATFDDELQG--DAKAKKHSTTSEALGIGAQMNAKAVVLTHFSQRYQKIPVLQTVTDSE-----------------EEDPLLNPKEVAEDVPMEDE------**

**CheTrz1 (786) TDTTVLIHEATFDDALVG--DAKAKKHSTTSEALGIGAKMNAKAVVLTHFSQRYQKIPVLQTVTDGE-----------------QEDPLLDPSKTAEDPTLN--------**

**PtrTrz1 (786) ADTTVLIHEATFDDELIG--DAKAKKHSTTSEALGVGEKMNAKAVVLTHFSQRYQNIPVLQPVEDEQ-----------------ADN--LLPEDATEDVVDD--------**

**PnoTrz1 (786) RDTTVLIHEATFDDELLG--DARAKKHSTTSEALDIGSQMNAKAVVLTHFSQRYQKIPVLETVTEGE-----------------QEGPLLDPENTAEEADNEN-------**

**MfiTrz1 (813) KGSTVCIHEATFDDELQG--DAEAKNHSTTSEALNVAQKMGAKACVLTHFSQRYQKVPVLERADESE-----------------APA-----------------------**

**MycTrz1 (806) KDSTVCIHEATFDDELQG--DAEAKQHSTTSEALHVAQNMNAKACVLTHFSQRYQKVPVLERADAQD-----------------APA-----------------------**

**SscTrz1 (734) QGATLLIHEATFDDELQG--DAIAKKHSTTSEAMNIGKGMGARRILLTHFSQRYQKIPVMD-------------------------------------------------**

**CglTrz1 (844) RGAHLLLHECTFEDELGG--DALAKKHSTLSEALDVGRQMEARRILLTHFSQRYPKLPVVDEKALET-------------------------------------------**

**MthTrz1 (697) RGAHLLVHECTFEDELAG--DAAAKKHSTLSEALEVGRRMEARRILLTHFSQRYPKLPVVDEEALLR-------------------------------------------**

**TteTrz1 (737) RGAHLLLHECTFEDELGG--DALAKKHSTLSEALDVGRQMRARRILLTHFSQRYPKLPVINEAALGT-------------------------------------------**

**PanTrz1 (705) KGAHLLLHECTFEDGLKG--DAVAKKHSTISEALAVGRDMGARRILLTHFSQRYPKLPAPEGEGEKV-------------------------------------------**

**NcrTrz1 (977) KGSHLLIHECTFDDELIG--EAKAKKHSTAGEALDVGRKMGARRVLLTHFSQRYPKMQAPVLDELEG-------------------------------------------**

**NteTrz1 (977) KGSHLLIHECTFDDELIG--EAKAKKHSTASEALDVGRKMGARRVLLTHFSQRYPKMQAPVLDELEG-------------------------------------------**

**NdiTrz1 (977) KGSHLLIHECTFDDELIG--EAKAKKHSTASEALDVGRKMGARRVLLTHFSQRYPKMQAPVLDELEG-------------------------------------------**

**SmaTrz1 (990) KGSHLLIHECTFDDELIG--EARAKKHSTASEALDVGKKMGARRVLLTHFSQRYPKMQAPVLDELEG-------------------------------------------**

**MgrTrz1 (825) QGATLLIHEATFENEKLV--DAMAKKHSTMGEAMLVARRMKARRVLMTHFSQRYTSIPRDMAKRDS--------------------------------------------**

**FgrTrz1 (737) QGAHLLVHECTFDDDMIS--HAKKKMHSTMSEALGIAKQMKARKTLLTHFSQRYVKADSLKEQ-----------------------------------------------**

**FoxTrz1 (734) EGAHLLVHECTFDDDMLS--HAKKKGHSTMGEALEIARKMKARRTLLTHFSQRYVKADSLKRD-----------------------------------------------**

**FveTrz1 (737) EGAHLLIHECTFDDDMLA--HAKKKGHSTMGEALEVARKMKARRTLLTHFSQRYVKADSLKRD-----------------------------------------------**

**NhaTrz1 (734) EGAHLLVHECTFDDDMLS--HAKKKKHSTMGEALSVAHKMKARRTLLTHFSQRYVKSDSLKRE-----------------------------------------------**

**TatTrz1 (745) RGAHLLVHECTFGDDKQD--HAKAKKHSTMGEALGVAREMQARRTLLTHFSQRYSKSDSLKRDR----------------------------------------------**

**TviTrz1 (743) RGAHLLVHECTFGDDKQD--HAKAKKHSTMGEALWVAREMGVRRTLLTHFSQRYSKSDSLRRER----------------------------------------------**

**TreTrz1 (753) RGAHLLIHECTFGDDKQD--HAKAKKHSTMGEALGVAREMAARRTLLTHFSQRYSKSDSLRRER----------------------------------------------**

**AgoTrz1 (714) KHSDLLIHEATLDNELLA--VAKNKKHSTINEAIFVSIPWAPRKVILRHFSSCFLRHRKILA------------------------------------------------**

**CanTrz1 (716) KGSDLLIHEATLDNELIE--DAIKKRHSTINEAINVSNAMEAKKLILTHFSQRYPKLPSMDN------------------------------------------------**

**SceTrz1 (713) YNSDLLIHEATLENQLLE--DAVKKKHCTINEAIGVSNKMNARKLILTHFSQRYPKLPQLDN------------------------------------------------**

**ZroTrz1 (703) KNSDLLIHEATLDNELIE--DAIKKKHCTINEAIEVSNEMNAQKLILTHFSQRYPKAPQVND------------------------------------------------**

**VpoTrz1 (711) YKSDLLIHEATLDNELIE--DAILKRHCTIDEAIEVSNSMEANKLILTHFSQRYPKLPQIDN------------------------------------------------**

**LthTrz1 (705) RQSDLLIHEATLDNDLVE--DAIKKRHCTINEAIEVSNKMGARKLILTHFSQRYPKLPQIGN------------------------------------------------**

**KlaTrz1 (708) KNSDLLIHEATLENELKV--EAMKKRHSTINEAIKVSNTMNARKLLLTHFSQRYPKVPNTNR------------------------------------------------**

**PpaTrz1 (674) KNSDLLIHEATLEDELME--DALSKRHSTISEAIYVSMLMTARKLILTHFSQRYPKLPDMDSLKKVWTFIDG--------------------------------------**

**CalTrz1 (693) YGSDLLIHEASMDGNWIE--EAIAKKHSTMIEAVAVSKLMNCPKLILTHFSSRYGISNNCVPKNEL-------------------------QSCADNL------------**

**CduTrz1 (693) YGSDLLIHEASMDGNWIE--EAIAKKHSTMIEAVAVSKLMNCPKLILTHFSSRYGISNNCVPKSEL-------------------------QSCADNL------------**

**CtrTrz1 (692) YDSDLLIHEASMDGNWIE--EAIAKKHSTMIEAVAVSRNMNCPKLILTHFSSRYGISNNCVPKAEL-------------------------EECANEL------------**

**CpaTrz1 (690) HKSDLLIHEASLDSFWID--EAIAKKHTAMIEAVGVCQLMQCPKLLLTHFSTRYGMSNNCVPKSDL-------------------------SEEAKEL------------**

**CguTrz1 (690) YSSDLLIHESSLDHELIE--EALAKKHSTMIEAIEMSRLMNCGHVILTHFSTRYCGSANFVSDQASL------------------------ENLSEQL------------**

**PguTrz1 (690) YSSDLLIHESSLDHELIE--EALAKKHSTMIEAIEMSRLMNCGHVILTHFSTRYCGSANFVSDQASL------------------------ENLSEQL------------**

**DhaTrz1 (693) YGSDLLIHESSLDHELIE--EAISKKHSTMIEAITVSKLMNCAKVILTHFSTRYSNKANMLIENNEL------------------------IKLSTNL------------**

**PstTrz1 (692) RDSDLLIHESSLDNELIE--EAIAKKHSTMIEAINVARYMNCSKLILTHFSTRYSNKTNTVRNSEAL------------------------IALSQSL------------**

**CluTrz1 (692) SDSDLLIHEASLDNDLIE--EAIAKKHSTVVEAVRVAQLMGCPKVILTHFSARFSEKHSFIRDAEEY------------------------DRLCENL------------**

**YliTrz1 (701) RDCDLVIHEATLNNDLPE--EAILKKHCTFSEALGVCKDMEAKHVVLTHFSQRYPKLPELSALTLET-------------------------------------------**

**AmaTrz1 (801) DGTDVVLHEATFESDLQA--EARKKQHSTTAEAVDVFEKMGARKLLLTHFSQRYPKLPKIER------------------------------------------------**

**BdeTrz1 (644) KGAHFLLHEATFDDEKQQ--EAIDRRHCTINEAIQVGQEMQAKCLLLTHFSQRYPKLPNIDSPTNESVG-----------------------------------------**

**SpuTrz2 (691) RGASVVIHEATLEDDKVE--EALEKRHCTTGEAIQVAKSMEAQNLLLTHFSQRYPKIPILPNDVDDD-------------------------------------------**

**SpuTrz1 (744) GDADVLIHEATFEDDPTGSREAQDKRHSTTQEAVIVGGRMRAKWILLTHFSQRYPKMPSLNFSAWPAADQ----------------------------------------**

**ScrTrz1 (687) KDATVVIHEATLEDSMQE--IAVRKQHSTYSEALGVGQSMNAKNIILTHFSQRYPKLPDVKLTD----------------------------------------------**

**SocTrz1 (688) KDATVVIHEATLEDSMQE--IAVRKQHSTYSEALRVGQLMNARSIILTHFSQRYPKLPEVNLSD----------------------------------------------**

**SpoTrz1 (701) KDSTLLIHEATLEDSMHE--IAIKKQHSTYSEALEVAKKAGTKNVILTHFSQRYPKLPDIDIST----------------------------------------------**

**SjaTrz1 (654) FNADLLIHEATLEDTMQD--IAIKKQHCTHKEALDIAKQMQAKNVVLTHFSQRYPKLPDVHIES----------------------------------------------**

**ScrTrz2 (588) KNAAVLLHEATFEDDLHE--EAVNRFHSTVSEALMVAKRMQAQKLILTHFSTRSIDTSF---------------------------------------------------**

**SocTrz2 (588) KNATVLLHEATFEDDLRD--EAVNRYHSTISEALMIAKKMQAKQLILTHFSTRSTDNSS---------------------------------------------------**

**SpoTrz2 (589) IGASLLLHESTFEDDLKH--EAIQRQHSTASEALSVAQSMKAKALILTHFSQRSYDADF---------------------------------------------------**

**SjaTrz2 (569) RDAKLLIHEATLDDSLSQ--LAVEKRHCTFSEALLVAKKMKSQNTVLTHFSQRYKSDYVIRKLFP---------------------------------------------**

**CneTrz1 (774) KGATLLIHEATLEDDKPE--VAAVKGHSTFSQAINVGKEMGAKYILLNHFSQRYPKLPKLPMPTSVAPGVQAPSTSTAE-------------------------------**

**TmeTrz1 (763) KGATVLIHEATLEDDKPD--VAKEKGHSTFSQAVGVGRKMGASHILLNHFSQRYPKLPRLAPSSTFYP------------------------------------------**

**AbiTrz1 (720) SGATLLIHEASMADDDLE--MAQAKAHSTVGQAIQIARDAKAKNVLLTHFSARYPKIIPMTSKPQAVATE----------------------------------------**

**CciTrz1 (834) RNATLLIHEATMSDDQVE--LARQKKHSTFGQAIGIGRRMNARTILLTHFSARHPKIPMSVMDISSDPTTPTTTRQ----------------------------G----F**

**ScoTrz1 (775) KGSTVVIHEATMNDNERE--LAAQKAHSTVGQAIEIAEKMSADNVLLTHFSARYPRLPPAVLNRSGE-------------------------------------------**

**LbiTrz1 (764) RGATLLIHEATMADDQED--MAKRKGHSTFGQAITIGKRMKASNILLTHFSARYPKMPPSGLKPRAS-------------------------------------------**

**PosTrz1 (776) RGATLLIHEATMADNQEE--MAKKKAHSTFGQAVDIGRRMNAENILLTHFSARYPKLPPDALAPVSSTKTPTG-------------------------------------**

**SlaTrz1 (778) ANATLLIHEATMADDQVE--MARAKMHSTFGQAVNIGRSMNAQKILLTHFSARYPKLPPPAILSPPTSATSAS-------------------------------------**

**HanTrz1 (769) DGATLLIHEATMGDDQEA--MAHHKAHSTVSQAVGIGRQMNAKNVLLTHFSTRYPHMPAYLSRPGSPARGRKSS------------------------------------**

**PplTrz1 (796) KGATLLIHEASFADDQEE--MAEQKAHSTCGQAIDIGRRMNAQNILLTHFSARFPKMPPVKTGPSS--------------------------------------------**

**PhaTrz1 (795) MGSTVLIHEATMADEEKD--MAIEKRHSTISEAIGIGHKMRAQNIMLTHFSARYPNRAPQELNPEVLPSP---G------------------------------------**

**AbiTrz2 (717) YGTDLLIHEASLKKGAEE--LALKRGHSTITQAIHVGHDMRAKNVLLTHLPPYAVTLPSTHLAKQYEN------------------------------------------**

**MglTrz1 (796) KGATLLVHEATMQDDEAE--LAAAKGHSTIGQACRVARDMKAEHLLLTHFSQRYPKLARLDVGGSTRGAYG--------------------E------------------**

**MlaTrz1 (702) VDADLLIHEASLGVDQKD--LADFKGHCTIDQAIEVGLEMKAKNCILNHFSTRFPKIPRLPEQTN---------------------------------------------**

**PgrTrz1 (689) QHADVLIHEASLGPEETE--LADTKGHSTIDQAIQAALKMNAKNCVLNHFSGRYPKIPPSINKS----------------------------------------------**

**AbeTrz1 (887) ---------AEDKKPRQAPSVVRDIPTSEDVEEDY----------------TMAEAEES-GAAKVTRPRSRSRSKSPSSSTNKPPIVIAFDYMRLRVADAIQAEAHQPLL**

**TruTrz1 (885) ---------AGDNKPRQAPSVVRDIPTSEDVEEDY----------------TMAETDES-EAAKATRPRSRSRSKSPSTSTNNPPIVIAFDHMRLRVADAIQAEAHQPLL**

**MgyTrz1 (885) ---------AADKKPRQAPSVVRDIPTSEDVEEDY----------------TMAEG----AAGKTSRPASRSRPKSPSSSANNPPIVLAFDHMRLRVADAIQAEAHQPLL**

**McaTrz1 (888) ---------AADQKPRQAPSVVRDIPTSEDAEEDY----------------TLSEETAAQTNTRPTRSTSSHRAKSPS-STNNPPIVLAFDNMRLRVADAIHAEAHQPLL**

**CimTrz1 (882) -------------F--VMPNAVRDIPATEETPQ-------------------GPETSDTADDLSAKEDLSLQKAL------DDVPVILAFDYMRLRLGDALHAEAHMPAM**

**CpoTrz1 (882) -------------F--VMPNAVRDIPATEETQQ-------------------GLETSDIADDLSAKEDLSLQKALG-----DDVPVILAFDYMRLRLGDALHAEAHMPAM**

**UreTrz1 (884) -----------------PPTAVRDIPATGDEQQSA----------------ETPEAPDTTDDLASKGDACLQKSLG-----DDVPVILAFDYMRLRLGDALHAEAYMHAM**

**AcaTrz1 (931) ASGSPLFNKLPARIAAWRANNPPDVPVSENDAASNGSDNNGDIDGDDDGGNARGLDSEYRTPMYGAPTPDVMQLDGG----VPVPVVVAFDMLRVRVGDMLCAQRYVPVL**

**AdeTrz1 (945) ESGSPQPNRRPHDIGEWRANNPPDVPVSKNDLG-N------DNGSDYGNGDGRGSGSRWRDQIRSGPAPAMMQLDG-----VPVPVIVAFDLLRVRVGDMLCAQRYVPVH**

**PbrTrz1 (915) SPNRPTSPGAAMKRTSSRGYNTPDVPISRNEHDND--------------------DYESETSVRAPRAPNVMRLGD-----VPVPVVVAFDLMRLRIGDVFCAQRYVPVL**

**AclTrz1 (894) ------------HNPLPVR-ENLDVPDDEAEA----------------------------EAEAAPPAAFKPQPQSQ---TLKAPVTAAFDYMRIRVGDIPIAQAFAPAI**

**AfuTrz1 (889) ------------PKPAPMTRVDLDVPDDEPEP----------------------------APEPVPSTLTVTPAENENKPPLQAPVTAAFDYMRIRVGDIPIAQAFAPAI**

**NfiTrz1 (889) ------------PKPAPMAREDLDVPDDEP------------------------------VPESVPSTLTVTPAENENKPPLQAPVTAAFDYMRIRVGDIPIAQAFAPAI**

**AcrTrz1 (892) -------------PNNHVGQVGLDVPDDEPE-----------------------------PTASNNRRQPFGDIKVTSRPRLSVPIVAAFDYMRIRVGDMPIAQAHAPAV**

**AspTrz1 (893) -------------PSQHVGQVGLDVPDDEP------------------------------PQNNTTRNQMFGDIKVTSRPKITVPIVAAFDYMRIRVRDMPIAQAYAPAV**

**AflTrz1 (894) -------------QETVVQPEQPDIPDNEPEEASQ-------------------------APASNGVPSFFSTIKVEGKPQVKVPIVAAFDYMRIRVGDMPIAQAYAPAV**

**AorTrz1 (894) -------------QETVVQPEQPDIPDNEPEEASQ-------------------------APASNGVPSFFSTIKVEGKPQVKVPIVAAFDYMRIRVGDMPIAQAYAPAV**

**AteTrz1 (894) ------------TDQPPVSNDVPDIPDNEPG-----------------------------DAAPELQGPVDSAPDAEPQLPLKAPVVAAFDYMRVRVGDMPLAQAYAPAV**

**PchTrz1 (885) -------------DAAAKEPADADIPFDDPQDEPVG------------------------PNNTELSDDFNSVTSNQRFGVYTGPVAGAMDYMRIKVGDFALAQAYAPAL**

**AniTrz1 (908) ------------IPFKNDIDAQENIALDEPAALNPD------------------------ATESLELSEPSELPEPDAPPRPIVPIIAAFDHMRVRVRDMYTLEQYAPAV**

**PmaTrz1 (862) ------------REAFKKRKVALDIPDDDC-------------------------------ADNG-AEVEVSQASGEDFDKATMPVCMAFDYMKVKIRDIPIAQMFMPAF**

**TstTrz1 (867) ------------RATFKMQKGTLDIPDDDP-------------------------------PEDG-ADDEVTQALESDFDRPEMPVCMAFDYMGVKLRDIPIAQMFMPAF**

**AbrTrz1 (871) ---------EAEVDPTIDNADNMDMHPTIPSSTTS----------------APGPAKPIPSLSHETSSSLRENARVIKVRNKDMKVAIAFDYMRVKIGEICELEKFNDAL**

**CheTrz1 (869) ---------DDEVDPTLDNADNMDIHPTIARPPR-------------------DSAKPIPALQRQ-SSSLAKMEQVVKIRNKDLKVAIAFDYMRVRIGDICELEHFTDAL**

**PtrTrz1 (867) ---------EADADPTTENADNMDIHPAKPSLQH----------------------------------QPTTGERVIKIRNKDMKVAIAFDYMRVKIGDICEMEKFNDAL**

**PnoTrz1 (870) ---------DIEADPTLENTDNMDIHSTTQLSST--------------------TAPKVATLERHASSTVPELGRVIKVRNPEMKVAIAFDYMRVKIGDIVQLEKFNEAL**

**MfiTrz1 (881) --------------EED----QYDIPSKKQASRS--------------------------------FGQSGPPEAVKFKLQSDMKVCVAFDYMRVKVGDIWKMEHFTPAL**

**MycTrz1 (874) --------------AG-----RYELPSNP--SRF--------------------------------GSNGGPPEAVKFKLNSDMKVCVAFDYMRVRVGDIGHMEKFTPAL**

**SscTrz1 (793) ------------------------------------------------------------------------------SEVTDQVAIVAFDYMKVKISDFSKIAAFRPAL**

**CglTrz1 (909) --------------------DG----------------------------------------------------------RHDMDVLFAFDMMRVKLGEFKQAGLFLPAL**

**MthTrz1 (762) --------------------NSTDGGDGGNNGGSSG-----------------------------------------GQKKRDVEVLFAFDMMRVRLGEFKQAKQFLPAL**

**TteTrz1 (802) --------------------EEEK-------------------------------------------------------ESRDVDVLFAFDMMRLKLGEFKQAKAFLPAL**

**PanTrz1 (770) --------------------ELG----------------------------------------------------------KDTAVLYAFDHMRVKLGEFKQAEEFIPAI**

**NcrTrz1 (1042) --------------------QEQSVS---------------------------------------------------GEKKDRTVVLYAFDYMKIKLGEFKMAERFLPAL**

**NteTrz1 (1042) --------------------QEQSVS---------------------------------------------------GEKKDRTVVLYAFDYMKIKLGEFKMAERFLPAL**

**NdiTrz1 (1042) --------------------QEQSVG---------------------------------------------------GEEKDRTVVLYAFDYMKIKLGEFKMAERFLPAL**

**SmaTrz1 (1055) --------------------SEEKK----------------------------------------------------GLGGDRTVVLYAFDYMKIKLGEFKMAERFLPAL**

**MgrTrz1 (889) -------------------------------------------------------------------------------ADGGMIVLFAFDHMRAKLGEFTEAVEFLPAM**

**FgrTrz1 (798) ------------------------------------------------------------------------------KRDDSGDVLMAFDHMKVKLGDFRKAAAFQPVI**

**FoxTrz1 (795) ------------------------------------------------------------------------------ERGRAGEALMALDLMSVKLGDFKKAAAFQPAI**

**FveTrz1 (798) ------------------------------------------------------------------------------ERGQAGEALMALDLMSVKLGDFRKAAAFQPAI**

**NhaTrz1 (795) ------------------------------------------------------------------------------DAGEAGEVLMAFDHMRVRLGDFKKAAAFQPAI**

**TatTrz1 (807) ------------------------------------------------------------------------------VAGVEHDVLLAFDFMAVKLGDFQKAACYLPSV**

**TviTrz1 (805) ------------------------------------------------------------------------------VEGVEQDVLLAFDFMAVKLGDFQKAACYLPSV**

**TreTrz1 (815) ------------------------------------------------------------------------------VEGVEHDVLLAFDFMAVRLGDFQKAACYLPSV**

**AgoTrz1 (774) -----------------------ALK------------------------------------------------------LKAEACCYAFDGMIINYKSLGQQVTKLRYL**

**CanTrz1 (776) -----------------------NIK------------------------------------------------------VEANEFCFAFDGMIIPYNTLGKQKEVFPML**

**SceTrz1 (773) -----------------------NID------------------------------------------------------VMAREFCFAFDSMIVDYEKIGEQQRIFPLL**

**ZroTrz1 (763) -----------------------NIK------------------------------------------------------ILAKEYCYAFDGMIVDYETLGEQESVFDQL**

**VpoTrz1 (771) -----------------------NKL------------------------------------------------------ILAKEYCFAFDGMIVDYDRIGEQQKYFPIL**

**LthTrz1 (765) -----------------------SIE------------------------------------------------------IEAQEYCFAFDGMIVGFDEIGEQTPHLRTL**

**KlaTrz1 (768) -----------------------SIK------------------------------------------------------LNARELCFAFDGMIVSFNELGKQQDKIDLL**

**PpaTrz1 (744) -----------------------------------------------------NYDENLELFQKSYVFKKFQLDAQQRFNAKQMGILYAFDNMVINYNEIDQQLQQIDAF**

**CalTrz1 (764) -------------------DTYLNKSHCELNIFQSQS--------------------------------------PSDLSLEEIDLWFAYDLMGVCYGDMHKQEYVWPIL**

**CduTrz1 (764) -------------------DTYLNKSHCELNIFSSES--------------------------------------PSNLNLEEIDLWFAYDLMGVCYGDLHKQEQAWPLL**

**CtrTrz1 (763) -------------------NSYLSENTSDQNIFRAKS--------------------------------------NSNLEFKDIDIWFAYDLMSVRYGNMHTQEKVWPIL**

**CpaTrz1 (761) -------------------ESQLIEKKVFDIFRNVN---------------------------------------VD--QLANIDIVFAYDLMNIRYGDFNTQEEKWSLL**

**CguTrz1 (762) -------------------RNYLLSTGATPNIFEYGLR-----------------------------------SERPTKSFEDVVVCFAFDMMVVNLRSMAKQKDSIKEI**

**PguTrz1 (762) -------------------RNYLLSTGATPNIFEYGLR-----------------------------------SERPTKSFEDVVVCFAFDMMVVNLRSMAKQKDSIKEI**

**DhaTrz1 (765) -------------------RDYLVRYGSTSNIFVPERS-----------------------------------NKRPIKEFEDLQICFAFDMMNVRFNNLHLQKDKYKEI**

**PstTrz1 (764) -------------------NNYLTKYRVSPNIFALEG------------------------------------QAYPIKSYDELDICYGYDFMNYRFKDLHLQKQKMDLI**

**CluTrz1 (764) -------------------KAYIGRS--TTNVFTMNE---------------------------------------SKLGFDDIDICYASDFMTIRYNDLACQKPFYAKL**

**YliTrz1 (766) ------------------------------------------------------------------------------KDLQKVPVAIAFDMMRIRLGEIAEQADHFDAI**

**AmaTrz1 (861) -------------------------------------------------------------------------------AMRPETIAVAFDLMAVPFRQFGELAKHAGAI**

**BdeTrz1 (711) -------------------------------------------------------------------------------TATGPIVGAAFDLMRIPIQYFWKFPKMMPAL**

**SpuTrz2 (756) --------------------------------------------------------------------------------LTQVKVGIAYDLMRAKLSSFWRLPLLVPGL**

**SpuTrz1 (814) -------------------------------------------------------------------------------EQRKSSVGVAFDMMAIKIGQMRRMAKYFGPL**

**ScrTrz1 (749) ---------------------------------------------------------------------------------KHLNVALAFDGMCLKVYEIPKFRNYIQPL**

**SocTrz1 (750) ---------------------------------------------------------------------------------QQLNVALAFDGMCLKSYEIPKFKNYIEPL**

**SpoTrz1 (763) ---------------------------------------------------------------------------------EDLHIALAFDGMTLKISDISLFRYFGKPL**

**SjaTrz1 (716) ---------------------------------------------------------------------------------DSPNVALAFDGMAIRIHEISQFQHFVEPL**

**ScrTrz2 (645) -------------------------------------------------------------------------TAPNWSIYPKHKTIYARDGMRWTQSICQPNKNAHKS-**

**SocTrz2 (645) -------------------------------------------------------------------------NGPNWSIYPKHKTIHARDGMRWTQSTFRSDKKNS---**

**SpoTrz2 (646) -------------------------------------------------------------------------LPPDWTIYPKSKTIYANDGLQWQQFQSKQRETI----**

**SjaTrz2 (632) --------------------------------------------------------------------------------KLPKNVVLACDNLHLVF-------------**

**CneTrz1 (851) ------------------------------------------------------------PNGVVDEIATLSASSTSLEVSTEPIVSISFDFMSLRLGDMWKMPYYMEGL**

**TmeTrz1 (829) -----------------------------SYPTIDQTDQTIP---------------STSEAVASKGTDPSHTISSPLQPDPEPIISISFDLMSIRVGDMWKMSHYMEAM**

**AbiTrz1 (788) ---------------GEEIMAQQPENTSGREEPITQMQETTLEGGDVLVLPQEYTVEREIQTMQNIRQDTVVQPQENTFQGEEPVVALALDLAEMSLDKIWKMNMYLPAI**

**CciTrz1 (910) -------------------------------------------------------------------------------HKTEPTIALAFDQSCLRIGDMWKMKHYLPAL**

**ScoTrz1 (840) -------------------------------------------------------------------------------ATARPLVTMAFDHARMTIGTMWKVNTYLPAI**

**LbiTrz1 (829) -------------------------------------------------------------------------------GSTEPTVAVAFDHVNLTIGNMWKIGYYLPAL**

**PosTrz1 (847) -------------------------------------------------------------------------------GPKEPIIALAFDHANIKIGDMWKLNHYMDAI**

**SlaTrz1 (849) -------------------------------------------------------------------------------IAKEPILALAFDHASIKIGDMWKMNTYMGAI**

**HanTrz1 (841) -------------------------------------------------------------------------------GGEGPVVALALDHARVRVGDMWKLRAYLRAI**

**PplTrz1 (860) --------------------------------------------------------------------------------GRGRTLALALDHTRVKIGDLWKLDAYLPAI**

**PhaTrz1 (864) -------------------------------------------------------------------------------GTPAPVVTLAFDHARVRIGEFAKLRAYLPAI**

**AbiTrz2 (783) -----------------------------------------------------------------------------TDDWGGPVVAVANDFMEIDLDKFWKANLCNEAI**

**MglTrz1 (866) ------------------------------------------------------------NKGVDDCQHQDQERDDRMASQDVPPIGIAFDMMRMTPAQLRRMVAGHRAM**

**MlaTrz1 (765) -------------------------------------------------------------------------------IDRKMNIGISFDLMTCRIDEVWKLERYLPAF**

**PgrTrz1 (751) -------------------------------------------------------------------------------NDQEMNIVISFDFMSCKIGAIRELQKCIPAL**

**AbeTrz1 (971) TKFLETTSDE----------------------------------------------------------------------------------------------------**

**TruTrz1 (969) TKFLETTSDE----------------------------------------------------------------------------------------------------**

**MgyTrz1 (966) TKFLETTSDE----------------------------------------------------------------------------------------------------**

**McaTrz1 (972) TKFLETTPEEC---------------------------------------------------------------------------------------------------**

**CimTrz1 (952) KEHLSASEVEF---------------------------------------------------------------------------------------------------**

**CpoTrz1 (953) KEHLSASEVEF---------------------------------------------------------------------------------------------------**

**UreTrz1 (956) KEHLSTSGVEF---------------------------------------------------------------------------------------------------**

**AcaTrz1 (1037) NRYYDAKQVMAVEHEERR--DRARALAGKDGGASGLDVGKGKQ----------------------------------KQGKKGPKQEGGGKRDGQHGRRQSGSG-----A**

**AdeTrz1 (1043) NLYYDSRLARAAHYGGWRQRENRHTEADAGADASAGDGAKEKQ----------------------------------KPWKKGWMKEEEGNKDKRRGKRQSSSSS----T**

**PbrTrz1 (1000) NRYYKYKEAAELSNEGVNQ-KRSSSGTGAGGKEKRNDQGKRDM----------------------------------KS-KKDWRGGKGGKRDDQRGRLRDSGPGPNPGS**

**AclTrz1 (960) EKLFEILERAAAEESAKHRKVREAADALAREKKLKKRAKGG-----------------------AAPAPTPAAPAGQAMDLDADKPSVWSASESESGWTTSGDESVRSGR**

**AfuTrz1 (959) EKLFDILERASAVEANKQKKIREAADALAKEKKQKQKKKGAA-------------------GAEPLSSATPAAATGEAMDIDQGR-SVWSASESENGWGSNDDEWMDRFW**

**NfiTrz1 (957) EKLFDILERASAVEANKQKKIREAADALAKEKKQKQKKKGAP-------------------EPEPAPSATPAAG--EAMDIDQGR-SVWSASESESGWGSNDDEWMDRFW**

**AcrTrz1 (960) EKLFDLLERAGAEEAN------ERREAAKQIVENRQKAKKA---------------KQTKHKQISADAETPAEMEIDRPAAAAKTKSIWSASESESGWSTSGSDSEGERV**

**AspTrz1 (960) EKLFDLQERAGEEKAE------QRKEAAKQIAQNRQKAKKQ---------------KQEKHKQIPAAAVEEMEVEKSE-GAGAKTKSIWSASESESGWSTSGSDSEGERI**

**AflTrz1 (966) EKLYDILERASEEDSEKQRQQKEKQEAAKMQEKMRRKAKHE-----------KKSKAGASQADVEPTPAVPAEEMDLDKKSPVKRHSAWSASESESGWSTSGSDSEAEMR**

**AorTrz1 (966) EKLYDILERASEEDSEKQRQQKEKQEAAKMQEKMRRKAKHE-----------KKSKAGASQADVEPTPAVPAEEMDLDKKSPVKRHSAWSASESESGWSTSGSDSEAEMR**

**AteTrz1 (963) GKLIDIFERASAQETEKAKLELEKQMAVKAEAKKSKKKLKQEKLAAESAKAAKPAQPADSTKPAGSESAVPAEAPADANKEPSK--SVWSASESESGWDTSGSDSEDAAL**

**PchTrz1 (958) EKLIELLERAATEEAERVKQKRQEEENARKAKNNKKWAKQMAT-----------------------ATAAAVAAAATETSEAVATRSVWSASESEEGWETSDYEECCS--**

**AniTrz1 (982) ERLFDIIERASKLEQNQAREKRRREVEENEKRKEFRRENKFKTKQEQ-----MSQEQREMAKAEKEARVSDRSSSQSRPQSPDAKVSIWDAPESESGWSSDESGKDA---**

**PmaTrz1 (928) EKLIERLDRLAEDESAVAKEESRKALEKKMKAKLGKKK---------------------------------FQALPTRSFSTDARKSAWSASESESGWTDESDVEGEQLE**

**TstTrz1 (933) EKLIERLDRVAEEESAVTREKARMALEAKQARTGKKKSGVK---------------------------S--PQRLSSRSFSTEAKKSAWSASESESGWSDEGEGEPE--Q**

**AbrTrz1 (956) NELLVKDEDADSALEGATKGAN-GNGKRASEDDGESKEG---------------------------------------KGKKKQKKPKEKSKRNN---------------**

**CheTrz1 (950) NTLLLKEVEADGETVDGEASEGKGSAKRASESDGEGRKGNKKK----------------------------------VKTKQVQKGKKEKSVRNN---------------**

**PtrTrz1 (934) NALLVTEDEEVNAEGEADGAGANANGKRVSEDDGDGKTN----------------------------------------GKKKQKKQKEKNKRNN---------------**

**PnoTrz1 (951) SELLVKEEEVEAIGAEDGKINSNGKKTSGDEGGGGGKKQ-K-------------------------------------QGQGTPKVRKMKSSQRNN--------------**

**MfiTrz1 (941) LKLFAEEEKEVKESGQEGGSAKKDKKNKFGKSKRNN--------------------------------------------------------------------------**

**MycTrz1 (931) --------------------------------------------------------------------------------------------------------------**

**SscTrz1 (825) LKLYEEKE------------------------------------------------------------------------------------------------------**

**CglTrz1 (941) RDLLKEEERA-----------DAEAGGV----------------------------------------------------------------------------------**

**MthTrz1 (811) RELLKVEERMGGGDDGGDEVKEEEEEEKQKKQKKKKKECNHQQRQGRLPSHPDG--------------------------------------------------------**

**TteTrz1 (837) RELLQEGEKQ-----------EEEDEE-----------------------------------------------------------------------------------**

**PanTrz1 (802) KKLLEEEMKEKEGEDG--EEGEEGGEEAAKKAEKKKEQERKKAEKQKKKLEHAKAAMEQKKGKGKNRGKVAEMVKEGEGGEKPEGTEKPEEKPEEVTSSEGDAKSEEVKT**

**NcrTrz1 (1081) RELYGELENE-----------EEDGGGAKE--------------------------------------------------------------------------------**

**NteTrz1 (1081) RELYGELENE-----------EEDGGEAKE--------------------------------------------------------------------------------**

**NdiTrz1 (1081) RELYGELENE-----------EEDGGEVKA--------------------------------------------------------------------------------**

**SmaTrz1 (1093) RELYGELENE-----------EEDVEVKE---------------------------------------------------------------------------------**

**MgrTrz1 (920) QKMYDTVLAKEGDDSGTSVPDLAAGQVGEELAHEDVDVAV----------------------------------------------------------------------**

**FgrTrz1 (830) AQLLVKTNTSG---------------------------------------------------------------------------------------------------**

**FoxTrz1 (827) AMLMADAGDK----------------------------------------------------------------------------------------------------**

**FveTrz1 (830) AMLMADAGDK----------------------------------------------------------------------------------------------------**

**NhaTrz1 (827) AQMLADAGDK----------------------------------------------------------------------------------------------------**

**TatTrz1 (839) QRFMEKLAD-----------------------------------------------------------------------------------------------------**

**TviTrz1 (837) QRFMEKLAD-----------------------------------------------------------------------------------------------------**

**TreTrz1 (847) QRFMEKLAD-----------------------------------------------------------------------------------------------------**

**AgoTrz1 (807) RGLFAEEEDI-----------ESGQD------------------------------------------------------------------------------------**

**CanTrz1 (809) SKAFAEEQQE-----------EEEESNDD---------------------------------------------------------------------------------**

**SceTrz1 (806) NKAFVEEKEE-----------EEDVDDVESVQDLEVKLKKHKKN------------------------------------------------------------------**

**ZroTrz1 (796) NQVFMEEKQE-----------EEDESQS----------------------------------------------------------------------------------**

**VpoTrz1 (804) KKIFISEEEE-----------EKHEEKQEDVVDK----------------------------------------------------------------------------**

**LthTrz1 (798) NEVFIEEQTS-----------EANEEESV---------------------------------------------------------------------------------**

**KlaTrz1 (801) GSIFVEEEND-----------AKKEDVKGLGNI-----------------------------------------------------------------------------**

**PpaTrz1 (801) GRERLELLFS-----------SKEKEL-----------------------------------------------------------------------------------**

**CalTrz1 (817) SELFLPSAEV-----------DHEKINEK-KEVKRLERLAMMK-TKKKRKMSPK--------------------------------------------------------**

**CduTrz1 (817) TELFPPSAEI-----------DHEKINEK-KEAKRLERLAIMK-VKKKRKMSPK--------------------------------------------------------**

**CtrTrz1 (816) KETFKPNSEV-----------DVEKINEK-KEIKRIERLAFME-KKKKKKRRTSSM------------------------------------------------------**

**CpaTrz1 (811) QNMFEVNS-------------KKRKLNEEEKDDEIIDTL-----------------------------------------------------------------------**

**CguTrz1 (818) MEIFQSDEPEADGENQPKGAKEAKKKEEK-QELKRLQRLALSS--QKKRRVNDEEV------------------------------------------------------**

**PguTrz1 (818) MEIFQSDEPEADGENQPKGAKEAKKKEEK-QELKRLQRLALSS--QKKRRVNDEEV------------------------------------------------------**

**DhaTrz1 (821) LEIFQTDDDLNE-DLNDKREKEMKKQREK-REAKRIQRLSIKN-GKKKRRVSSDEEID----------------------------------------------------**

**PstTrz1 (819) YDTFEAEEDD-----DKK-ERETSKQREK-QEVRRMQRLQSKNSVHKKRKVSGSEDEEDQ--------------------------------------------------**

**CluTrz1 (814) SELSTSATSEAEVAKSQK---EQLKKSEK-REAKRLQRLSKKK----RR-LSNESV------------------------------------------------------**

**YliTrz1 (798) AKALEDGERGEADDVPAW--------------------------------------------------------------------------------------------**

**AmaTrz1 (892) RAVCSYQQQAVEQVDSAKNE------------------------------------------------------------------------------------------**

**BdeTrz1 (742) KLLFPPEE------------------------------------------------------------------------------------------------------**

**SpuTrz2 (786) RALFPQDDNDGVNR------------------------------------------------------------------------------------------------**

**SpuTrz1 (845) EKLYPAAQEESPDLEEGELERAADLSGGGGGGEGKQDGGSRRGGRGRGRGRGRGRGRGHRGGHGGHRGDFHRSRDR----------------------------------**

**ScrTrz1 (778) AYLFADDDTADN--------------------------------------------------------------------------------------------------**

**SocTrz1 (779) AHLFADDDCH----------------------------------------------------------------------------------------------------**

**SpoTrz1 (792) AYLFNEENLKEESDPLKF--------------------------------------------------------------------------------------------**

**SjaTrz1 (745) KTLFSEKPIES---------------------------------------------------------------------------------------------------**

**ScrTrz2 (678) --------------------------------------------------------------------------------------------------------------**

**SocTrz2 (680) --------------------------------------------------------------------------------------------------------------**

**SpoTrz2 (678) --------------------------------------------------------------------------------------------------------------**

**SjaTrz2 (649) --------------------------------------------------------------------------------------------------------------**

**CneTrz1 (901) SMLFAEPEDGEDVVEEAQATEGVNAGGKEKKEKGGKKDKGHGKESAIAKGSGGGKGNDTVVTEPALKSKRSLKKEAARAIKAKAEDERVAASMTGVAVGGVQKEAKEKRA**

**TmeTrz1 (895) SLLFASEPEESEGMVEGDVNSVGTRQKKRGSGEALLDEVVSAKEDAVGTESGGGAGKGSTTVRKEGQKTDMHDGKKKRRAEKAKRAGKGLSGGDGMISGSGVGTMEGNVL**

**AbiTrz1 (883) EQCYDDSKEDGDEDIPA---------------------------------------------------------------------------------------------**

**CciTrz1 (941) EQNEKDTVAIDGEDEGVEGAMDLDPSA-----------------------------------------------------------------------------------**

**ScoTrz1 (871) KQCMEDSADADDEGVDASVTVEADVVS-----------------------------------------------------------------------------------**

**LbiTrz1 (860) QQSFYDTIEEGDEEEAAA-IEAVDMDCDLT--------------------------------------------------------------------------------**

**PosTrz1 (878) QQCLRDSSDDGDEEEIT--LESTEVDIS----------------------------------------------------------------------------------**

**SlaTrz1 (880) EQCFIDTTEDGDEEENTDALGMTQVDIV----------------------------------------------------------------------------------**

**HanTrz1 (872) EQSFHDTFEEGDEEEEQQALKASAEQVELE--------------------------------------------------------------------------------**

**PplTrz1 (890) ECNFADTVAEEGDAIRLSMDQNDPPSPSGEPRDVAVNGGKEEGSFWAPLGSDEVRGGFALAASARALYAARGTRNARDGNALIVVKLYAIAENVGQFSIRPVCLTEDKRS**

**PhaTrz1 (895) EQSVNDVAAQDPADDDELELDPTKASCIIMLHGCDLVRDDADDADAE-------RRRGVIDCLPRSDHARRRDNRVRR-RGRITGGLWAGEACTAAAAVRNLDTHD----**

**AbiTrz2 (816) ADCHHIAFQEEKKSIAQLLWELLPEESLVCSPNIAYLFSSPPERKTGKARSQNRRVPEARSDVRGARKAWSEDRKAPEALSNVHGVPKTRIKDSRAPKAWSDFREVPKAR**

**MglTrz1 (916) SLLLETEPEAIDEPTGPALSSSSSSSLSALLAGAQAEPTAFSPPKTKRARNLYASQTRFNFHYLVLTFTSRNKALRPPSELSVSEGTRRALQSAFGSIGGAVAVDVMYAG**

**MlaTrz1 (796) EYLFKEPEKEEIETEVGLEDLKPVEKSKNKNKTKKDQKKDVASKGTEGNSKNKKNQKNQKNQKSIDLPSKDGKICVENGVKGEGISKDAEVVIDESKESTTEKVSTDEKC**

**PgrTrz1 (782) EQMVEG-LDIEDTTDAGPSDPSPG--SGKKTKSKKKAGDQTCTKGSRVRQLSKEDVESLAN-QVVPEPLNIDSIDME---------------------------------**

**AbeTrz1 (981) ------------------------------------------------------------------------------------**

**TruTrz1 (979) ------------------------------------------------------------------------------------**

**MgyTrz1 (976) ------------------------------------------------------------------------------------**

**McaTrz1 (983) ------------------------------------------------------------------------------------**

**CimTrz1 (963) ------------------------------------------------------------------------------------**

**CpoTrz1 (964) ------------------------------------------------------------------------------------**

**UreTrz1 (967) ------------------------------------------------------------------------------------**

**AcaTrz1 (1106) ADVPWLDQRGHDRQDEAQLSTGGMGG----------------------------------------------------------**

**AdeTrz1 (1115) SSATTRVDHGGHGEDAPQKVAGSMGD----------------------------------------------------------**

**PbrTrz1 (1074) GSGYASSFGYFGGHAGGQKAAGGMGG----------------------------------------------------------**

**AclTrz1 (1047) GRGHGRGRAGS-----SPRRLSPSIRTKSGDAGAGAGAA---------------------------------------------**

**AfuTrz1 (1049) RGKHSRSRSHSRSKSASGRRSSPSIRTKSGDAV---------------------------------------------------**

**NfiTrz1 (1045) RGKHSRSRSHSRSKSRGGRRSSPSMRTKSGDAV---------------------------------------------------**

**AcrTrz1 (1049) KLGRKRRAS--------GQRAK--------------------------------------------------------------**

**AspTrz1 (1048) KKEHYARKRND------GGAAKGAQ-----------------------------------------------------------**

**AflTrz1 (1065) ARRTSRSPSGT------VKRSS--------------------------------------------------------------**

**AorTrz1 (1065) ARRTSRSPSGT------VKRSS--------------------------------------------------------------**

**AteTrz1 (1071) SNEKKQ------------------------------------------------------------------------------**

**PchTrz1 (1043) ------------------------------------------------------------------------------------**

**AniTrz1 (1084) ------------------------------------------------------------------------------------**

**PmaTrz1 (1005) KDNKAL------------------------------------------------------------------------------**

**TstTrz1 (1012) KDTKA-------------------------------------------------------------------------------**

**AbrTrz1 (1011) ------------------------------------------------------------------------------------**

**CheTrz1 (1011) ------------------------------------------------------------------------------------**

**PtrTrz1 (989) ------------------------------------------------------------------------------------**

**PnoTrz1 (1009) ------------------------------------------------------------------------------------**

**MfiTrz1 (977) ------------------------------------------------------------------------------------**

**MycTrz1 (931) ------------------------------------------------------------------------------------**

**SscTrz1 (833) ------------------------------------------------------------------------------------**

**CglTrz1 (958) ------------------------------------------------------------------------------------**

**MthTrz1 (865) ------------------------------------------------------------------------------------**

**TteTrz1 (853) ------------------------------------------------------------------------------------**

**PanTrz1 (910) QEGVEKLEVKESEDVVMT------------------------------------------------------------------**

**NcrTrz1 (1100) ------------------------------------------------------------------------------------**

**NteTrz1 (1100) ------------------------------------------------------------------------------------**

**NdiTrz1 (1100) ------------------------------------------------------------------------------------**

**SmaTrz1 (1111) ------------------------------------------------------------------------------------**

**MgrTrz1 (960) ------------------------------------------------------------------------------------**

**FgrTrz1 (841) ------------------------------------------------------------------------------------**

**FoxTrz1 (837) ------------------------------------------------------------------------------------**

**FveTrz1 (840) ------------------------------------------------------------------------------------**

**NhaTrz1 (837) ------------------------------------------------------------------------------------**

**TatTrz1 (848) ------------------------------------------------------------------------------------**

**TviTrz1 (846) ------------------------------------------------------------------------------------**

**TreTrz1 (856) ------------------------------------------------------------------------------------**

**AgoTrz1 (822) ------------------------------------------------------------------------------------**

**CanTrz1 (827) ------------------------------------------------------------------------------------**

**SceTrz1 (839) ------------------------------------------------------------------------------------**

**ZroTrz1 (813) ------------------------------------------------------------------------------------**

**VpoTrz1 (827) ------------------------------------------------------------------------------------**

**LthTrz1 (816) ------------------------------------------------------------------------------------**

**KlaTrz1 (823) ------------------------------------------------------------------------------------**

**PpaTrz1 (817) ------------------------------------------------------------------------------------**

**CalTrz1 (858) ------------------------------------------------------------------------------------**

**CduTrz1 (858) ------------------------------------------------------------------------------------**

**CtrTrz1 (859) ------------------------------------------------------------------------------------**

**CpaTrz1 (837) ------------------------------------------------------------------------------------**

**CguTrz1 (871) ------------------------------------------------------------------------------------**

**PguTrz1 (871) ------------------------------------------------------------------------------------**

**DhaTrz1 (876) ------------------------------------------------------------------------------------**

**PstTrz1 (872) ------------------------------------------------------------------------------------**

**CluTrz1 (861) ------------------------------------------------------------------------------------**

**YliTrz1 (816) ------------------------------------------------------------------------------------**

**AmaTrz1 (912) ------------------------------------------------------------------------------------**

**BdeTrz1 (750) ------------------------------------------------------------------------------------**

**SpuTrz2 (800) ------------------------------------------------------------------------------------**

**SpuTrz1 (921) ------------------------------------------------------------------------------------**

**ScrTrz1 (790) ------------------------------------------------------------------------------------**

**SocTrz1 (789) ------------------------------------------------------------------------------------**

**SpoTrz1 (810) ------------------------------------------------------------------------------------**

**SjaTrz1 (756) ------------------------------------------------------------------------------------**

**ScrTrz2 (681) ------------------------------------------------------------------------------------**

**SocTrz2 (679) ------------------------------------------------------------------------------------**

**SpoTrz2 (679) ------------------------------------------------------------------------------------**

**SjaTrz2 (649) ------------------------------------------------------------------------------------**

**CneTrz1 (1011) GSPGLEGPDRKRRSTDVEESGVEEV-----------------------------------------------------------**

**TmeTrz1 (1005) GEKPEVGGQEVV------------------------------------------------------------------------**

**AbiTrz1 (900) ------------------------------------------------------------------------------------**

**CciTrz1 (968) ------------------------------------------------------------------------------------**

**ScoTrz1 (898) ------------------------------------------------------------------------------------**

**LbiTrz1 (889) ------------------------------------------------------------------------------------**

**PosTrz1 (904) ------------------------------------------------------------------------------------**

**SlaTrz1 (908) ------------------------------------------------------------------------------------**

**HanTrz1 (902) ------------------------------------------------------------------------------------**

**PplTrz1 (1000) RTSTGNGIAVRSSLSHPFHFFGAGHLSCFLTIPTSI------------------------------------------------**

**PhaTrz1 (993) ------------------------------------------------------------------------------------**

**AbiTrz2 (926) SEDNRAPKKISKKRVFSNNGIQ--------------------------------------------------------------**

**MglTrz1 (1026) LPLDPSVAPDGAVGEAVLRVASEHVDTLASALSSIQGQAHATMTGQQEEMRVSVRGTSHNVGALRSNTSRAWIQQQLQMHESKP**

**MlaTrz1 (906) IVEQS-------------------------------------------------------------------------------**

**PgrTrz1 (855) ------------------------------------------------------------------------------------**
