# Supplementary material for: Identification and analysis of candidate fungal tRNA 3'-end processing endonucleases tRNase Zs, homologs of the putative prostate cancer susceptibility protein ELAC2
Source: BMC Evol Biol. 2010 Sep 6;10:272. doi: 10.1186/1471-2148-10-272 (PMC2942849; doi:10.1186/1471-2148-10-272)
Supplement: Additional file 4 — Alignment of candidate fungal tRNase ZSs. Similar or identical amino acid residues are shaded as described in the legend to Figure 2. The conserved motifs are labeled according to references [30,31,44]. [file 1471-2148-10-272-S4.DOC]

Additional data file 4. Alignment of candidate fungal tRNase Z^S^s

**PxKxRN loop motif I**

**AbiTrz3 (1) ---------------------------------------MQLTFLGTASAQ-PSSTRNHSSLALRLGGDVWLF---------DCGEATQHQIQKSS-------TVKMGRI**

**CciTrz2 (1) --------------------------MAAVVQKRSLLQSLGITFLGTASAQ-PSSTRNHSALALRLNRDVWIF---------DCGEATQHQLQKST--------VKMGKI**

**PplTrz2 (1) -------------------------------MPPATLSSVNITILGSASAQ-PSSTRNHSAFALRLGGDVWLF---------DCGEATQHRVQKST--------VKMGRI**

**LbiTrz2 (1) ------MTCYRRLIQIANVFGRRRTMSSSSASRQSPIGSLSATFLGTASAQ-PSSTRNHSALALRVGGDVWLF---------DCGEATQHQIQKSN--------VKMGKI**

**HanTrz2 (1) -----------------------------------MISSINITFLGTASAQ-PSSTRNHSALALRLSGGVWLF---------DCGEATQHQIQRSQ--------VKMGKI**

**SlaTrz2 (1) -----------------------------MSAGRRVFSSLNITFLGTASAQ-PSSTRNHSSLALRLGGDVWLF---------DCGEATQHQIQKST--------VRMGKI**

**PhaTrz2 (1) -----------------------------------MVATISITFLGTASAQ-PSSTRNHSSLALRLDGDVWLF---------DCGEATQHQVQKSN--------VKMGKI**

**ScoTrz2 (1) ------------------------------MTSTKPLGGVNVTFLGTASAA-PSSTRNHSSLALRLDGDVWLF---------DCGEATQHRLQKSQ--------VKMGKI**

**PosTrz2 (1) -------------------------------MSRPPITSASITFLGTASAQ-PSSTRNHSSLALRLGGDVWLF---------DCGEATQHQLQRST--------VKLGKV**

**AbiTrz4 (1) -----------------------------------MAHPMSVSFLGTSSGGGPTENRNCSSLICDFLGGNNNL------WMVDCAEGTARQFALQP-NRQSATRLKMSQV**

**LbiTrz3 (1) MLFLPQRILRIRPINSCRRPPRRASSHIRYLDNRKMPQHMSVLFLGTSSGGGPSESRNCSSLVCEFLKDSSLWS----SAVVDCAEGTIRQFQFQP---SNTYQVRPNKI**

**CciTrz3 (1) ----------------------------------MFRQNLSLMFLGTASGGGPSETRNCSSLLCDFMDDLSLW-------LVDCAEGTLRQFQFQP---QEGFKYRANRV**

**PplTrz3 (1) ------------------------------------MHDITVTFLGTTSGGGPSETRNCSSLVVQPLGDARLW-------MVDCAEGTLRQFSQQPYH-DAARRLRIGTL**

**SpuTrz3 (1) ---------------------------------MPTIGNIDIVFLGTASAQ-PSPTRNHSSLALRFDGRIWLF---------DAGEGTQHQIIQSD--------LKAGKV**

**MglTrz2 (1) ---------------------------------MVHPPPMQVKFLGTCTSP--LLTRNYSRFVNVESASWFGYANAIIEVCFDCGEGTQRQLLLPHVN----SHTKLAQI**

**MlaTrz2 (1) -------------------------MGSKS----HPPDQLEVNFLGTSAGK-PTTFRNPSSLAVRMDGDMWMF---------DCGEATTHQMMRTT--------LKASNV**

**PgrTrz2 (1) --------------------MEKRIRSSDSNRSINPPNDFEITFLGTSAGK-PTIQRNPSSLALRMDGQIWMF---------DCGEATTHQIMRTN--------LKPSNV**

**TmeTrz2 (1) -----------------------------MSKEPKQPSNVSVHFLGTCAGAGPVVSRNCSSLAVDFGNEVWVF---------DAADGTLGRLHQSS--------LKMSNI**

**CneTrz2 (1) ----------------------------MAAPKIKPLPPVSVQFLGTSSGGGPIQSRNCSSLAVDFGNEIWLF---------DTADGTLNRLHQSS--------LKLANI**

**motif II**

**AbiTrz3 (55) RKIFITHMHGDHIFGIAPLLASCLNGAGGTAEG--------------YEDPRSQHSGMAPMVEIYGPLGIRAYVRTALSYTHTKLDGTYVVHELRSPSDPQH--------**

**CciTrz2 (67) EKVFITHTHGDHIFGLVPLLASCMNGAGGVVEEQ-----------GQGEDPRRVVDTSIPPLEIYGPMGTRAYIRSALACTYTLLGRPYVVHELRFPSDPEG--------**

**PplTrz2 (62) QKIFITHTHGDHIFGLLPLLASRLDGAGGVVDAD-----------DPR--TKAESRHAVPPLEIYGPLGTRAYIRSGLTYTHTLLGAPYVVHELRFISDPP---------**

**LbiTrz2 (87) EKIFVTHTHGDHIFGLIPLLASCLNGAGGAADG--------------AEDARAQADTTIPPLEIYGPAGTRGYVRSGLKYTHSLLGKRYVVHELRFPSDPQE--------**

**HanTrz2 (58) EKIFITHTHGDHIFGILPLLASRLNGAGGITEGE-----------DS----RAEEVLDQEPIEIYGPLGTRAYIRNGLSYSHTLLGGPYVVHELRFPTDPQD--------**

**SlaTrz2 (64) EKIFITHTHGDHIFGLLPLLASRLNGAGGVAEGV-----------DDP---RAQLSDFSNPFEIYGPLGTRAYVRNGLTYSHTLLGSPYVVHELRFPSDPPD--------**

**PhaTrz2 (58) QKIFITHTHGDHIFGLLPLLASRLNGAGGMADGA-----------DDPRIQN--LASNMATAEIYGPYGTRAYVRTGLKYTHTHLGSPYVVHELRMAHDPPPA-------**

**ScoTrz2 (63) QKIFITHTHGDHMFGVVPLLCSLLNGAGGTTEGA-----------DDPRTR---VDKSLPPIELYGPQGLRAYVRGGLTYSHSLIGSPYVVHELRFPSDP----------**

**PosTrz2 (62) EKIFITHTHGDHIFGLIPVLASRLNGAGGTVDDA-----------DDP---RHQVDHQMPPLEIYGPYGTRAYVRSGLLYTHTLLSAPYVVHELRTPSDPP---------**

**AbiTrz4 (69) SKLFITHMHADHIMGITTFLRNILGAPRIDSPPP---------------PSNAISHRRAPIIQIYGPSGLRSFLRQNLKMTFTRCENTYVVHELLCKDDPVIPCNPP---**

**LbiTrz3 (104) TKIFITHMHADHIMGIVPLLRNVLFPPPAGSQPSTSSHRALVSFTLSCNRRRILSISFQPKIEIYGPAGIRTFVRQIMKMTFTNTADNYTVHELLSENDPVTPCDH----**

**CciTrz3 (67) TKIFITHMHADHIMGIVPFLRCVLYPPEAGKKP---------SYTSNPP---------SPKVEIYGPAGLRTFVRQIMKMTLTYTADCYTVHELLTKDDPVTPCDLP---**

**PplTrz3 (67) SKVFITHMHADHIGGILTVADHIGGILTVLRNALG--------IPPAHQCSPAPPLPTIPTIEIYGPRGLRRFVRLQMRLTHSHTATRYAVHELLAPGETQSVLAG----**

**SpuTrz3 (60) EKIFVTHLHGDHCFGIPGLLCTLSQAAGGWCPG----------------------EDKDMKFEIFGPKGTREYIRNALKSTYSRLGCRYTVHELHFPDDQSTPPSE----**

**MglTrz2 (72) RTILITHLHPDHILGLTPLIFSMMGLSASVPKDG------------------------KPRLQLVGPLGLRAFLRATLSITYASLSSHFIVHELLWPSQPAYPHNPEGIS**

**MlaTrz2 (64) KKIFITHLHGDHVLGLISFLAHISDRIDADLMSE-----------N-----QHPFSNPSASIEIFGPSGIREFVRTNLRLTQTHAALKVKINELLRPSRDRVYGPS----**

**PgrTrz2 (73) TKIFITHLHGDHVLGLISFLSHIGDRADADLTSK-----------N-----QHPFCDPSEVVEIFGPSGIREFVRSTLRLTKTHSLLKIKVNELLRYSKDQVYTPS----**

**TmeTrz2 (65) TRIFITHMHADHVLGLVPILTTIMSGVGVTDDDL-----------EELR--Q-KGTSKKPTVNIYGPAGLRKLVRTTINLTSLILRGAYAVHELFTD--TP---------**

**CneTrz2 (66) SRIFITHLHVDHVLGLVPVLTTVMTGTMATKEAT-----------QKIK--E-LGLKKQPTFHLYGPSGLRNLVRTILNITQANLSGVFAIHELLQDGEEP---------**

**motif III**

**AbiTrz3 (143) ------------------GDYTTVLQLPLEKE-GTNFIQVNG--IWNDIYKDARL---------SVSAAPILHS-----------------VPCVGYVLHEFPIPGKIDP**

**CciTrz2 (158) ------------------DIAHGFTPPPAESA-GRNIAMVNG--VWPDIYRDEVV---------SVSAAPILHS-----------------VPCVGYVVTEAPVPGKIDA**

**PplTrz2 (150) ------------------LDPTALPPHSFELP-GRDLQQVDG--VWPEIYRDDLV---------TVSAAPIMHS-----------------VPCVGYVVTELPVPGKMDP**

**LbiTrz2 (175) -------------------EEVFPLGNAESPH-GQDIPQVDG--CWRNIFTDSTI---------SVSAAPIKHS-----------------VSCVGFVVTEAPIPGKIDP**

**HanTrz2 (145) ------------------GDFTSLPRLPFESPTGRNISQANG--IWTDIFKNDLI---------SVSAAPILHS-----------------VPCVGYVVTEAPVPGKIDP**

**SlaTrz2 (152) ------------------GDFTSLPLHASESPHGRNISQSDG--VWPSVYEDDAV---------SVSAAPILHS-----------------VPCVGYVVTESPVPGKMDP**

**PhaTrz2 (148) ------------------EDSSTLPLDPAELPTGRNIVQESDG-TWRDIFASDAL---------TVSAAPIYHS-----------------VPCVGYVVQERPVPGKINP**

**ScoTrz2 (149) ------------------DVESYLPLHPAELE-GRNITQTADG-IWHNIFADDRV---------SVSAAPILHS-----------------CPCVGYVVQEAPVPGKMDP**

**PosTrz2 (149) ------------------GDNISLPLHSAELPSGRDILQSEDQPLWTDIYKDAVV---------SVSAAPIFHS-----------------VPCVGYVVTEAPVPGKIDP**

**AbiTrz4 (161) ----VDEHDPMSG-----SDFKDWDVLHCSEVPGRDIRADDQG-LWRDIARETNPRR---ATQIQIHASPILHRSKLMHITRAFVLLTLYLEPCIGYVFDEISYP-----**

**LbiTrz3 (210) ---------ADPLHAVSRFAIAEQNILHVNEVEGKDIRASADG-SWKNITGDLG--------DIVVNAGPIEHR-----------------DPCIGYVFTETQAP-----**

**CciTrz3 (156) ----PLDESAAAAESGFDHNIANPNVMHVSEVRGSDIRADKKG-FWKSFVSARGRVQ-----DIEVDAGSIQHR-----------------DPCIGFVFRETGPP-----**

**PplTrz3 (165) ----------AG---TRTEDGDGDDIPLENEALGQDVLCGPDG-FWRGIASDAVAGGLKSGGRVVVDAGPIQHR-----------------DPCIGYIFREVPHLPFHLR**

**SpuTrz3 (144) -----------------TLHNDEIAGRDIQLTSGSNAAHPNESHWSVLHDPETDM---------TVTAAPIAHT-----------------VPCIGYVIQEPPHPGNINI**

**MglTrz2 (158) TFSYTEQDPYLPEGVRGQVRTLPLLPPHENELPGRDIRMNESTCTWPSILQLSN---------VTISAAPITHR-----------------CPTVGYVFQEGPTASRSVS**

**MlaTrz2 (154) -----------------QSKPDG--RLWHTEILGEDIWADEDG-VWRDIVGMDEAGV-------SVGAAPIAH------------------VDCVGYMLVEANRREKFDM**

**PgrTrz2 (163) -----------------LSNPTGSGRLWHNEILGEDIWSDQNG-LWKDVIPINQCGV-------SVSAAPIQHT-----------------IDCVGYLLTEANRREKFDM**

**TmeTrz2 (150) ------------------SVGCSEEELHVNEAVGMDLSADENG-VCENILLEGNGKQGK---GWAVSAGPIDHRGTKSSSD---------IVPSLGYVLQEPVPRLPLDT**

**CneTrz2 (153) ------------------SAGCREDEIHPNEAVGMDMRADEDG-VWKVVLQEGNGKNGK---GWKVSAGPIHHR-----------------VPSLGYVLQEPTPRLPLDT**

**flexible arm**

**GP motif Walker A-like motif motif IV**

**AbiTrz3 (206) KEYIPHIKR-----------TKTSMSVMQQLQQGSS---VKLTDGTVLQG----PPRREGRKVVILGDTYDPSPIISLACDADL------------------------LI**

**CciTrz2 (221) SKYKPHIVR-----------TKTPMSVMRQLQLGER---VVLNDGTILEG----PPRRPGRKVVILGDTYDPSPIRSLAMDADL------------------------LV**

**PplTrz2 (213) KLYIPHLKR-----------TKMPMSLMSRLQQGES---VELSDGTVLRG----PPRRPGRKLVILGDTYDPSPIADLAADADI------------------------LV**

**LbiTrz2 (237) KKYIPDIKR-----------TNTPISVMRQLQQGES---VQLGDGTILHG----PPRRKGRKVVILGDTYDPSPIIPLAEDADL------------------------LI**

**HanTrz2 (209) RQYIPHIKR-----------TGAPMTVMRRVQQGES---VELPDGTVLRG----PDRRQGRKLAILGDTYDPSPIADLAAGVDI------------------------LI**

**SlaTrz2 (216) KKYIPAIKR-----------TKTPMSVMSRLQQGES---VELADGTVLRG----PPRKPGRKLVILGDTYDPSPIAGLAYRPDL------------------------LV**

**PhaTrz2 (213) AMYVPHLKR-----------TNTPMSVLRQLQQGET---VQLADGTVLHG----PERRPGRKIVILGDTFDPSPIAALAQDADV------------------------LV**

**ScoTrz2 (213) KLYVPHIKR-----------TGAPMSLLASLQRGES---VTLPDGTTLAG----PARRPGRKIAILGDTYDPSPLAPIAADVDL------------------------LV**

**PosTrz2 (215) KQYAPELKR-----------TGAPMTLMRQLQQGES---VQLPDGTILHG----PPRRPGRKIVILGDTYDASPIIPLAKDADI------------------------LI**

**AbiTrz4 (253) -----------------------------------------------------------KRKVVILGDTCDPSPIIPLCSNPRP----------------------SLLI**

**LbiTrz3 (280) -----------------------------------------------------------YRKIVILGDTHDPSAIAPLCINPSP----------------------SLLI**

**CciTrz3 (234) -----------------------------------------------------------GRKIVILGDTYDPSQMTPLCLNPPP----------------------TLLV**

**PplTrz3 (244) AP--------------------------------------------------------QPRTLVILGDTYDPSAIVPLIESPDVLSSQPTDMVIDTNVPAAAPPTVSLLV**

**SpuTrz3 (211) STLQPHLERN--RKALAEQGIKNPSILIGKLMSQQS---LTLPDGTNLLLKEHLTPPTPGRKITILGDTNDPSPIIHLAIDSDL------------------------LI**

**MglTrz2 (242) QEELAIIDSN-KDALFELYNIRSPRSLLSRVMRDRET--ITLPDGHVLSPP---PLDRPGRKLCILGDTSDATAGLVGRGMAYLAR------------------DADLLV**

**MlaTrz2 (219) SKLNPIIREH--ADEIKQMGFRTPQAILSKLEQDRKP--ITFSSGVTLDPP---KLSIQGRKVVILGDTSDPTPILALTSKDPD-QK------------------IDLLV**

**PgrTrz2 (231) VKLKPILEQH--ADEIKQMGFKVLPAILSQLEKTRKP--ISFSTGATLQPP---RLSIRGRRVMILGDTCDPSAMVSLVDQDPEFQS------------------IDLLV**

**TmeTrz2 (229) SVLVPLLQAHADALAALDPPVRHPLSLLSHLTSLPTPKPYTLPSGEVLYPPE--PSGIPARKLVIFGDCTGGTENARFLEICSDPS---------------------LLI**

**CneTrz2 (224) ATLIPLLQSNSEALAALDPPVKHPLSLLSHLTSLPAPPPFTLPSGDIISPPE--ASGILPRKIIIFGDCSGGTKNHIFRSMCEDAS---------------------LLV**

**HEAT HST motif V**

**AbiTrz3 (274) HEATNAHLPGID-------------PTTKTTDTDESVEERAKSRGHSTPLMAGAFAKRVRAKKLVLNHFSSRYPGDD---------------------SEEAHKIMAAIG**

**CciTrz2 (289) HEATNAHLPGLD-------------PNTKADDTHESVEARTKSRGHSTPQMAGAFAKSIGARRLALNHFSARYPGDN---------------------SPESLKIMEGIA**

**PplTrz2 (281) HEATNSHLPGVD-------------PETKAEDTHASVEARAKSRGHSTPQMAGRFARRIRARKLLLNHFSARYAGNDDV-------------------DEQARSIMEAIK**

**LbiTrz2 (305) HEATNAHLPGID-------------PNTKEIDTYASVEERAKSRGHSTPQMAGAFAKRVRARKLVLNHFSARYPGDE---------------------TEEANKIMDAIV**

**HanTrz2 (277) HEATNAYLRGVV-------------AETKESDTVESVEARTKLRGHSTPQMAGAFAQRIAAKKLVLNHFSARYAGDDHK-------------------DPLARTTMNAIK**

**SlaTrz2 (284) HEATNSHLPELD-------------VNTKAEDTFEIVEARAKSRGHSTPQKAGAFAARIGAKKLVLNHFSSRYAGNDDV-------------------NAEAKTIMTAIA**

**PhaTrz2 (281) HEATNAHLPGID-------------PDTKPTDTYEIVEERAKSRGHSTPQMAGAFAKRIGAMRLILNHFSARYAGNDDV-------------------SEEAKKVMHAIR**

**ScoTrz2 (281) HEATNAHLPGVD-------------PATKAEDTYETVEARAKSRGHSTPQMAGAFAKRIGAKRLVLNHFSSRYPG--DE-------------------SEETSRIMGAIG**

**PosTrz2 (283) HEATNAHLPDVD-------------SHTKATDTYESVEERAKSRGHSTPQVAGAFATRTRARKLLLNHFSARYPGDDDC-------------------NEWSKKVMDAIK**

**AbiTrz4 (282) HEATDSTISPETDD-----------AGRLSKRQLPDVMKTTLARGHSTTSMAGEFAKLVNAQMLVLNHIGTRFPAPRINDNG---------------QISFARRVLEDLE**

**LbiTrz3 (309) HEATDAHIPAHADA-----------TGRLAKRRPEEVREKALLRGHSVPEMAGSFAKRVGAAKLVLNHIGGRFPAPRHS-------------------RDGRAMVIREIE**

**CciTrz3 (263) HESTDAPIPESADQ-----------EGKLSKRDPEEVLQKVLLRGHSVPGMAGAFAKQVKAAHLVLNHIGSRFPAPRNP-------------------WDARATVMQEFE**

**PplTrz3 (298) HEATDSYIPSSIDP----------QGRTGRNRSEASVFKKTLERGHSTPEMAGAFARRIGALRVVLNHIGARFPAPDHSGSY---------------ADKFRRATMREIE**

**SpuTrz3 (292) HEATNACLK----------------ADLASGQTPSSVQESTISHGHSTPEMAGAFARLVGARKLVLNHFSSRYKGDLS---------------------EESLQIMEEIR**

**MglTrz2 (328) HECTYASMNAEDLEMARIESEEHAQMLQKSLLGVQEAETRALSRGHSVPRIVGSFSGEIRARRVVLNHFSARLPAPVVSTEAPLTSTHQLKQGTPFKNSVKQFYVMREIE**

**MlaTrz2 (303) HESTGTSVVDLNPS-----------IISASDTNEASVAQKMRDRGHSTSFMAGQFARQINAKRMVMNHVGGKFPSPLGC-------------------VCPS----PELA**

**PgrTrz2 (316) HESTGTVVPDAHER-----------KQEDCQS-ESQVASKMRERGHSTSFMAGQFAHRVRATRLVLNHLGGKFPAPQAA-------------------LCPSKAFPPFPS**

**TmeTrz2 (316) HECTNAAIPELVCQTEKK------EREERETKHREVTRMKAFKNGHSTPLEVGTFAKTIRARRVAVNHFSAMFPAPR---------------------YPSSDPYPSILS**

**CneTrz2 (311) HECTNAAIPQAIQREEKG------QRARTRDLEPSEVKKKAQSRGHSTPDEVGEFARVIRARRVVVNHFSAMFPSPR---------------------YPTAAALPSILS**

**AbiTrz3 (350) SLAAGEYG---------------------HDVICARDFMSFDIVHS-------------HV-------------------------------------------------**

**CciTrz2 (365) KLAEHEFG---------------------REVICATDLLSIDVQLK-------------E--------------------------------------------------**

**PplTrz2 (359) ALAVSEFD---------------------GEVVCARDFMSFDVELR-------------QEG------------------------------------------------**

**LbiTrz2 (381) ELAAKEFG---------------------SSVTCAKDFMSVDVVSN-------------D--------------------------------------------------**

**HanTrz2 (355) ALAQENYN---------------------GPVVCARDFMSFDVEFS-------------SHS------------------------------------------------**

**SlaTrz2 (362) GLAGKEYQ---------------------GEIVCARDFMSFDVESV-------------PDVSPNNT-------------------------------------------**

**PhaTrz2 (359) ALAEQPYGDG-------------------KDVICARDLMVVDIEPR-------------R--------------------------------------------------**

**ScoTrz2 (357) RLAEGEYG---------------------KPVTCARDLMSLDVGLA-------------E--------------------------------------------------**

**PosTrz2 (361) SLAATEYE---------------------GEIVCARDFMTIDCKLP-------------ILD------------------------------------------------**

**AbiTrz4 (366) RHATEAWDPPLG-----------------HRAIVALDFMRVQVPLP-------------MRETNISTSNLVYSSTSVSAAGPSSVATSTLSLAQSSTMESDTLNSVSG--**

**LbiTrz3 (389) RQATQAWDSGER-------------------AMAAWDFMRVSIPVA------------EDMGYGRGIDS--------GEGE-----------IKTEVLEVDFDYRSVSAS**

**CciTrz3 (343) RQATEAWGVNSKKNASSRGHGRYGHGRGQQSAVAAQDFLRINIPMQGPAMFLFPPSQSKSSGYGDGQAHRRLRARIIGQGSRITREAQGMACQEPKEMRTGVMERIEAVV**

**PplTrz3 (383) RQATEAWQPPQ-----------------DVYAQAAFDFMRVVLPPHFR------------KIAHVSESHSSYADVAIAEVETTWQQRTESGGEVKKAQGGMSMEEVEDRV**

**SpuTrz3 (365) ALAVGTFMN--------------------EDVICARDFLSVEIVRR-------------RADGG----------------------------------------------**

**MglTrz2 (438) RQVTKYWHASLPEEFREFVKN--------DNAVAAFDGLAIDIAPHS-----------APTEQNTA--------------------------------------------**

**MlaTrz2 (379) KISEFPRV----------------------DRGERESLTIYDTCLK-------------STASGLWKTYEEMDDERQGKVFQEVKWIESVAADAVNGWKAGCGKEK----**

**PgrTrz2 (395) SSSSSPSS----------------------SVCAQSSIIEYQNAVK-------------MVHAEVWKKFEEIEPGRRAKIVDELGWLKAVENDALQGWRTAARNRTPPQP**

**TmeTrz2 (399) PMSPLPYPAP-------------------WPLRYSIDPPMSPVPLT-------------AAELHLRLILQSVTDQIDEVWDGPAFPLSLSTYPSID------PYTQVLSP**

**CneTrz2 (394) SICSFPYPVP-------------------RPLPHTSLPPPLPVPLNSK----------YPSELHVRVIMQSLADQILDKCASFSLSDEDLDKP----------------P**

**AbiTrz3 (377) -----------------------------------------------------------------------------------------------------**

**CciTrz2 (391) -----------------------------------------------------------------------------------------------------**

**PplTrz2 (387) -----------------------------------------------------------------------------------------------------**

**LbiTrz2 (407) -----------------------------------------------------------------------------------------------------**

**HanTrz2 (383) -----------------------------------------------------------------------------------------------------**

**SlaTrz2 (395) -----------------------------------------------------------------------------------------------------**

**PhaTrz2 (387) -----------------------------------------------------------------------------------------------------**

**ScoTrz2 (383) -----------------------------------------------------------------------------------------------------**

**PosTrz2 (389) -----------------------------------------------------------------------------------------------------**

**AbiTrz4 (444) --NSRSVEVTGHGVQGVFKPTKPKRKIG-------------------------------------------------------------------------**

**LbiTrz3 (449) GSAAWSTTTTTINTERHWEGNPRKKRRP-------------------------------------------------------------------------**

**CciTrz3 (453) AMEEVATDIPLLDLLRLVDP-PRRHTLVVTANPNTLMSLIVTLVTIMGTAVIPLMGDLLTMVIELAPPLLVIAMGIMDLESVGAKPSSQDHTD--------**

**PplTrz3 (464) VLGIGATHDRGESMIMEGDL---------------------------------------------------------------------------------**

**SpuTrz3 (396) -----------------------------------------------------------------------------------------------------**

**MglTrz2 (485) -----------------------------------------------------------------------------------------------------**

**MlaTrz2 (450) ----DEVDLEVVVAHDFLQVKVPRSDLI-------------------------------------------------------------------------**

**PgrTrz2 (470) RASNLPAQPRVSVAYDFLQFKVPRPDPHS------------------------------------------------------------------------**

**TmeTrz2 (471) NSQRSSSGRKSIATRDFMILPVPSHELSESEMSTIALVEGEGREVMTEWRERGGVWMGQGTERRWVGVEKE------------------------------**

**CneTrz2 (459) ---RGKTG-MVIPARDFMHLPIPSHELNALEIEDVRSCKELADKVTEEWKKHGGIWIENGEEE-WIGVEKEVKVTGWRFKKVAKESDEWVVDRVKEDSNGN**
