# Supplementary material for: Identification and analysis of candidate fungal tRNA 3'-end processing endonucleases tRNase Zs, homologs of the putative prostate cancer susceptibility protein ELAC2
Source: BMC Evol Biol. 2010 Sep 6;10:272. doi: 10.1186/1471-2148-10-272 (PMC2942849; doi:10.1186/1471-2148-10-272)
Supplement: Additional file 5 — Pairwise sequence comparisons of tRNase ZLs from Schizosaccharomyces species. The accession numbers for proteins are listed in Additional file 1. The pairwise percent identity (I) and percent similarity (S) between tRNase ZLs from Schizosaccharomyces species were calculated using the Clustal W program [49]. [file 1471-2148-10-272-S5.DOC]

Additional data file 5. Pairwise sequence comparisons of tRNase Z^L^s from *Schizosaccharomyces* species

| Protein | ScrTrz1 | SocTrz1 | SpoTrz1 | SjaTrz2 | ScrTrz2 | SocTrz2 | SpoTrz2 |
| --- | --- | --- | --- | --- | --- | --- | --- |
|  | I S | I S | I S | I S | I S | I S | I S |
| SjaTrz1 | 42 56 | 43 56 | 45 57 | 23 33 | 23 35 | 23 34 | 21 33 |
| ScrTrz1 |  | 82 89 | 57 70 | 22 32 | 21 33 | 22 33 | 20 31 |
| SocTrz1 |  |  | 56 68 | 21 33 | 21 31 | 22 33 | 21 32 |
| SpoTrz1 |  |  |  | 21 32 | 22 33 | 22 34 | 20 31 |
| SjaTrz2 |  |  |  |  | 30 42 | 29 40 | 28 43 |
| ScrTrz2 |  |  |  |  |  | 75 84 | 46 59 |
| SocTrz2 |  |  |  |  |  |  | 46 59 |
